# Supplementary material for: Robotic versus laparoscopic distal gastrectomy for resectable gastric cancer: a randomized phase 2 trial
Source: Nat Commun. 2024 May 31;15:4668. doi: 10.1038/s41467-024-49013-6 (PMC11143299; doi:10.1038/s41467-024-49013-6)
Supplement: Supplementary file 1 — Supplementary Information [file 41467_2024_49013_MOESM1_ESM.pdf]

Supplementary Information

Supplementary Table 1. Checklist for the determining the success of D2 lymphadenectomy

Scoring Method for D2 Lymph Node Dissection	Complete<br>10	Incomplete<br>5	None<br>0
1. Properly full omentectomy	□	□	□
2. Ligation of left gastroepiploic artery at origin	□	□	□
3. Ligation of right gastroepiploic artery at origin	□	□	□
4. Full exposure of common hepatic artery	□	□	□
5. Ligation of right gastric artery at origin	□	□	□
6. Exposure of portal vein	□	□	□
7. Exposure of splenic artery to branch of posterior gastric artery	□	□	□
8. Identification of splenic vein	□	□	□
9. Ligation of left gastric artery at origin	□	□	□
10. Exposure of gastroesophageal junction	□	□	□

1. Properly full omentectomy
   a. Omentectomy was performed close to transverse colon
   b. Omentectomy was performed from hepatic flexure to splenic flexure
   c. Anterior layer of transverse colonic mesentery and pancreatic anterior peritoneum was dissected.
2. Ligation of left gastroepiploic artery at origin
3. Ligation of right gastroepiploic artery at origin
4. Full exposure of common hepatic artery
   a. More than half of anterior part in the common hepatic artery were exposed.
5. Ligation of right gastric artery at origin
6. Exposure of portal vein

7. Exposure of splenic artery to branch of posterior gastric artery
   a. More than half of anterior part in splenic artery was exposed.
   b. Splenic artery was exposed from celiac trunk to posterior gastric artery
8. Identification of splenic vein
9. Ligation of left gastric artery at origin
10. Exposure of gastroesophageal junction
   a. Anterior and right side of the abdominal esophagus were exposed.

- D2 lymphadenectomy was accepted if all randomly assigned three investigators rated 85 points and more regarding checklists in unedited video review.

Supplementary Information

Supplementary Table 1. Checklist for the determining the success of D2 lymphadenectomy

Scoring Method for D2 Lymph Node Dissection	Complete<br>10	Incomplete<br>5	None<br>0
1. Properly full omentectomy	□	□	□
2. Ligation of left gastroepiploic artery at origin	□	□	□
3. Ligation of right gastroepiploic artery at origin	□	□	□
4. Full exposure of common hepatic artery	□	□	□
5. Ligation of right gastric artery at origin	□	□	□
6. Exposure of portal vein	□	□	□
7. Exposure of splenic artery to branch of posterior gastric artery	□	□	□
8. Identification of splenic vein	□	□	□
9. Ligation of left gastric artery at origin	□	□	□
10. Exposure of gastroesophageal junction	□	□	□

1. Properly full omentectomy
   a. Omentectomy was performed close to transverse colon
   b. Omentectomy was performed from hepatic flexure to splenic flexure
   c. Anterior layer of transverse colonic mesentery and pancreatic anterior peritoneum was dissected.
2. Ligation of left gastroepiploic artery at origin
3. Ligation of right gastroepiploic artery at origin
4. Full exposure of common hepatic artery
   a. More than half of anterior part in the common hepatic artery were exposed.
5. Ligation of right gastric artery at origin
6. Exposure of portal vein

7. Exposure of splenic artery to branch of posterior gastric artery
   a. More than half of anterior part in splenic artery was exposed.
   b. Splenic artery was exposed from celiac trunk to posterior gastric artery
8. Identification of splenic vein
9. Ligation of left gastric artery at origin
10. Exposure of gastroesophageal junction
   a. Anterior and right side of the abdominal esophagus were exposed.

- D2 lymphadenectomy was accepted if all randomly assigned three investigators rated 85 points and more regarding checklists in unedited video review.

Supplementary Table 2 Test of proportional hazards assumption among variables for disease-free survival and overall survival.

	Disease-free Survival			Overall Survival		
	**chi-square**	**df**	***p* value**	**chi-square**	**df**	***p* value**
Age, ≥65	3.458	1	0.063	0.731	1	0.392
Tumor size, mm	0.325	1	0.569	0.139	1	0.709
Female	0.049	1	0.826	0.875	1	0.350
ECOG PS, score 1	0.054	1	0.816	0.205	1	0.650
Surgical approach, robot vs lap	2.458	1	0.117	2.875	1	0.090
Lymphovascular invasion, yes vs no	0.362	1	0.547	0.763	1	0.382
Histology, undifferentiated	0.336	1	0.562	0.139	1	0.710
pT stage, T2-4 vs T1	0.372	1	0.542	0.372	1	0.542
pN stage, N+ vs N0	0.095	1	0.758	0.019	1	0.891
Adjuvant chemotherapy, yes vs no	1.813	1	0.178	2.439	1	0.118
Global	8.789	10	0.552	8.408	10	0.589

Abbreviations: df, degree of freedom; ECOG PS, Eastern Cooperative Oncology performance status; lap, laparoscopy

Supplementary Table 3 Multivariable Cox Regression Analyses of Risk Factors for Overall Survival

	Multivariate Analysis		
	**HR**	**95%CI**	***p* value**
Age, ≥65	-	-	0.621
Tumor size, mm	-	-	0.109
Female	-	-	0.629
ECOG PS, score 1	-	-	0.278
Surgical approach, robot	0.542	0.296-0.994	0.048
Lymphovascular invasion, yes	-	-	0.552
Histology, undifferentiated	-	-	0.640
pT stage			
T1	1.000		
T2-4	5.752	1.330-24.873	0.019
pN stage			
N0	1.000		
N+	4.423	1.535-12.745	0.006
Adjuvant chemotherapy, yes	-	-	0.234

Abbreviations: ECOG PS, Eastern Cooperative Oncology performance status

Supplementary Table 4 Comparison of Lymph Node Dissection of Patients Who Underwent Robotic or Laparoscopic Surgery

Characteristic	RDG (n=141)	LDG (n=142)	
	**Mean ± SD / N (%)**	**Mean ± SD / N (%)**	***p* value**
**Total retrieved LNs**	40.9 ± 11.2	39.9 ± 12.2	0.452
≥ 30	121 (85.8%)	111 (78.2%)	0.094
Perigastric regions	23.3 ± 8.6	24.0 ± 9.0	0.500
Extraperigastric regions	17.6 ± 5.8	15.8 ± 6.6	0.018
**LN compliance**			0.006
Compliant	106 (75.2%)	85 (59.9%)	
Noncompliant	35 (24.8%)	57 (40.1%)	

P values were calculated by chi-square test. Statistical tests were twosided without adjustment for multiple comparisons.

Abbreviations: RDG, robotic distal gastrectomy; LDG, laparoscopic distal gastrectomy; SD, Standard deviation; LN, lymph node.

Supplementary Table 5 Adjuvant Chemotherapy Characteristics of Stage II/III patients by group.

	RDG (n=86)	LDG (n=99)	
	**Median (IQR) / N (%)**	**Median (IQR) / N (%)**	***p*-value**
**Adjuvant chemotherapy**			0.768
Absent	18 (20.9%)	19 (19.2%)	
Present [a]	68 (79.1%)	80 (80.8%)	
**Chemotherapy regimens [b]**			0.749
Platinum based	7 (10.3%)	7 (8.75%)	
Docetaxel based	61 (89.7%)	73 (91.25%)	
**Surgical procedure–adjuvant chemotherapy interval, (days)**	28 (24-32)	32 (26-42)	0.003
**No. of cycles completed, median**	6 (3-6)	6 (3-6)	0.795
**Cycles of completed b**			
Cycle 3	55 (80.9%)	63 (78.8%)	0.748
Cycle 4	46 (67.6%)	55 (68.8%)	0.886
Cycle 5	43 (63.2%)	49 (61.3%)	0.804
Cycle 6 or more	41 (60.3%)	45 (56.3%)	0.619
**Adverse events**			
Grade 1-2	33 (48.5%)	41 (51.3%)	0.741
Grade 3-4	13 (19.1%)	14 (17.5%)	0.800

[a] 5-fluorouracil (5-FU) in combination with either platinum-based drugs or docetaxel.

[b] For patients with adjuvant chemotherapy.

P values were calculated by chi-square test or the Mann-Whitney U test. Statistical tests were two sided without adjustment for multiple comparisons.

Abbreviations: LDG, laparoscopic distal gastrectomy; RDG, robotic distal gastrectomy; IQR, interquartile range.

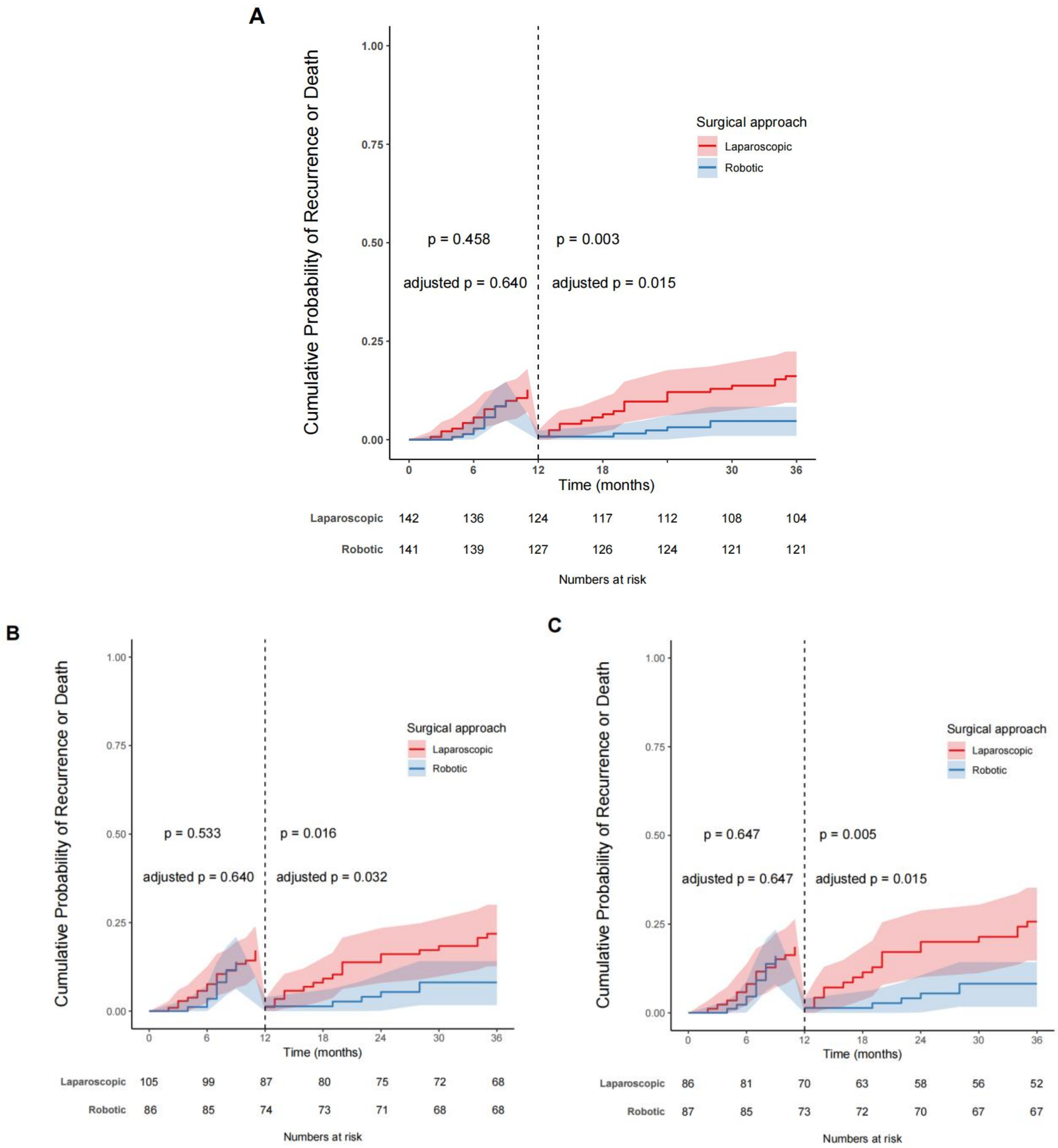

Supplementary Figure 1: Landmark analysis discriminating between events occurring before and after 1 year of follow-up. (A) whole population; (B) patients with pT2-4 stage; (C) patients with pN+ stage. p-values for all survival analyses have been calculated using the log-rank test. The shadows on either side of the survival curves indicate 95% confidence intervals. Adjusted p value was calculated using Benjamini – Hochberg method.

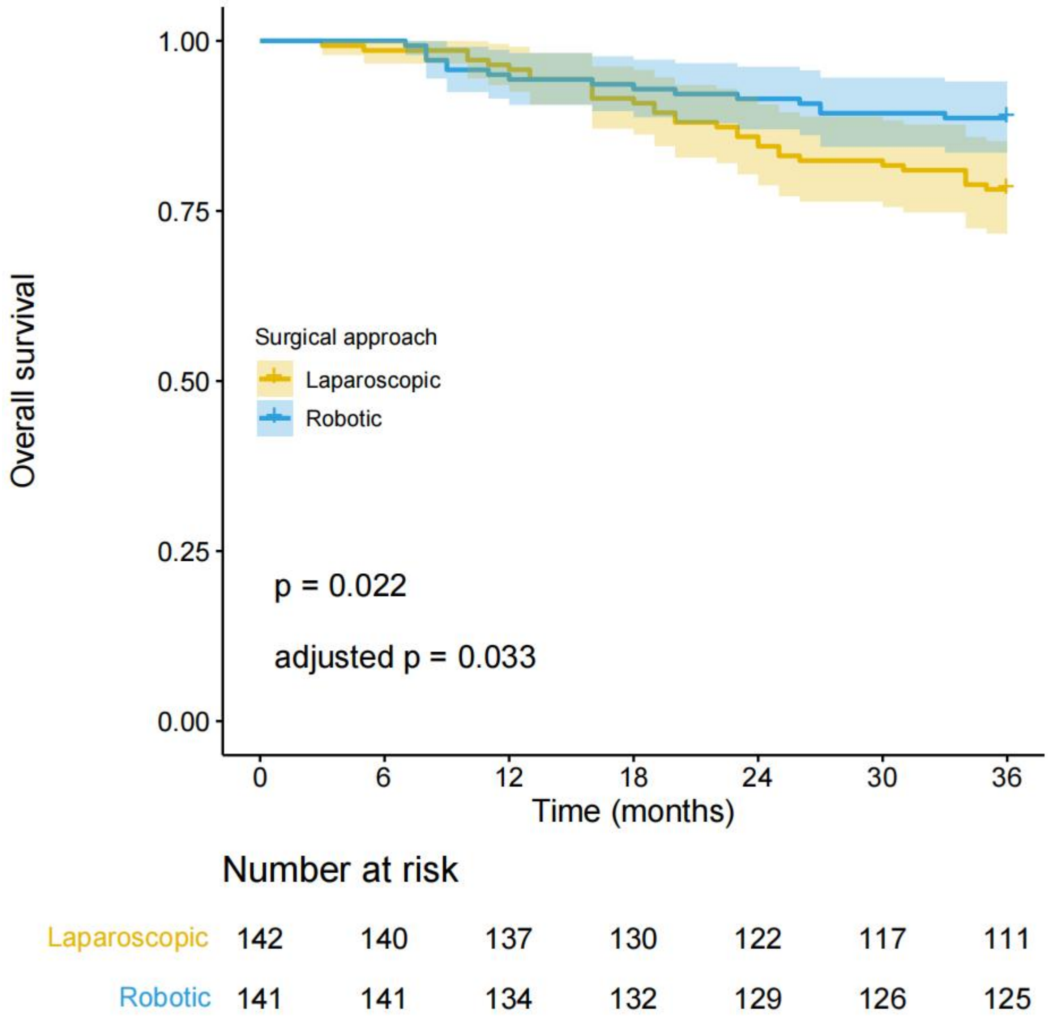

Supplementary Figure 2: Kaplan-Meier curves of overall survival for robotic and laparoscopic distal gastrectomies within 3 years after surgery. The shadows on either side of the survival curves indicate 95% confidence intervals. p-values for all survival analyses have been calculated using the log-rank test. Adjusted p value was calculated using Benjamini－Hochberg method.

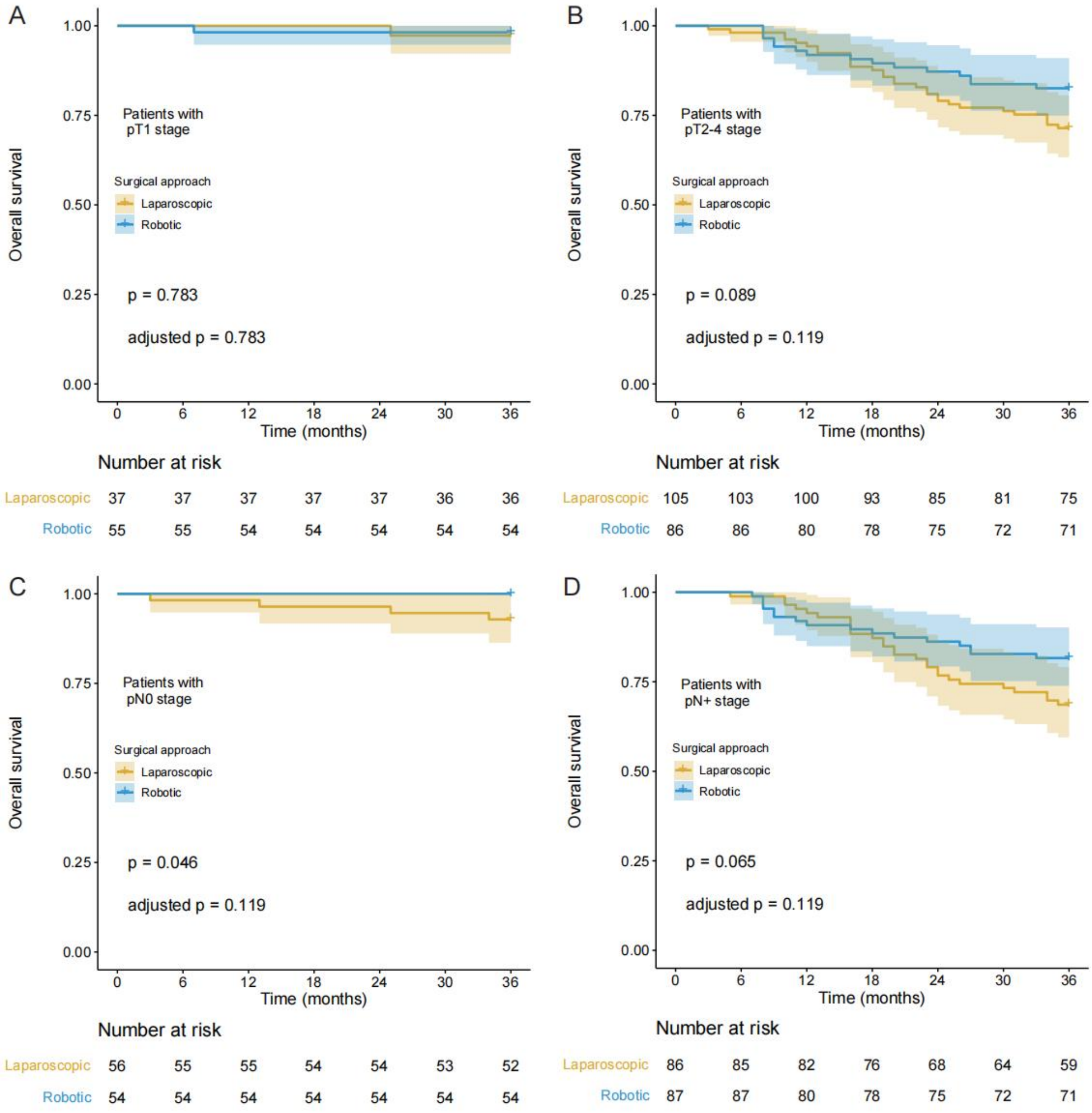

Supplementary Figure 3: Kaplan-Meier curves of overall survival for robotic and laparoscopic distal gastrectomies within 3 years after surgery by different pathologic T stage and N stage. (A) patients with pT1 stage; (B) patients with pT2-4 stage; (C) patients with pN0 stage; (D) patients with pN+ stage. The shadows on either side of the survival curves indicate 95% confidence intervals. p-values for all survival analyses have been calculated using the log-rank test. Adjusted p value was calculated using Benjamini–Hochberg method.

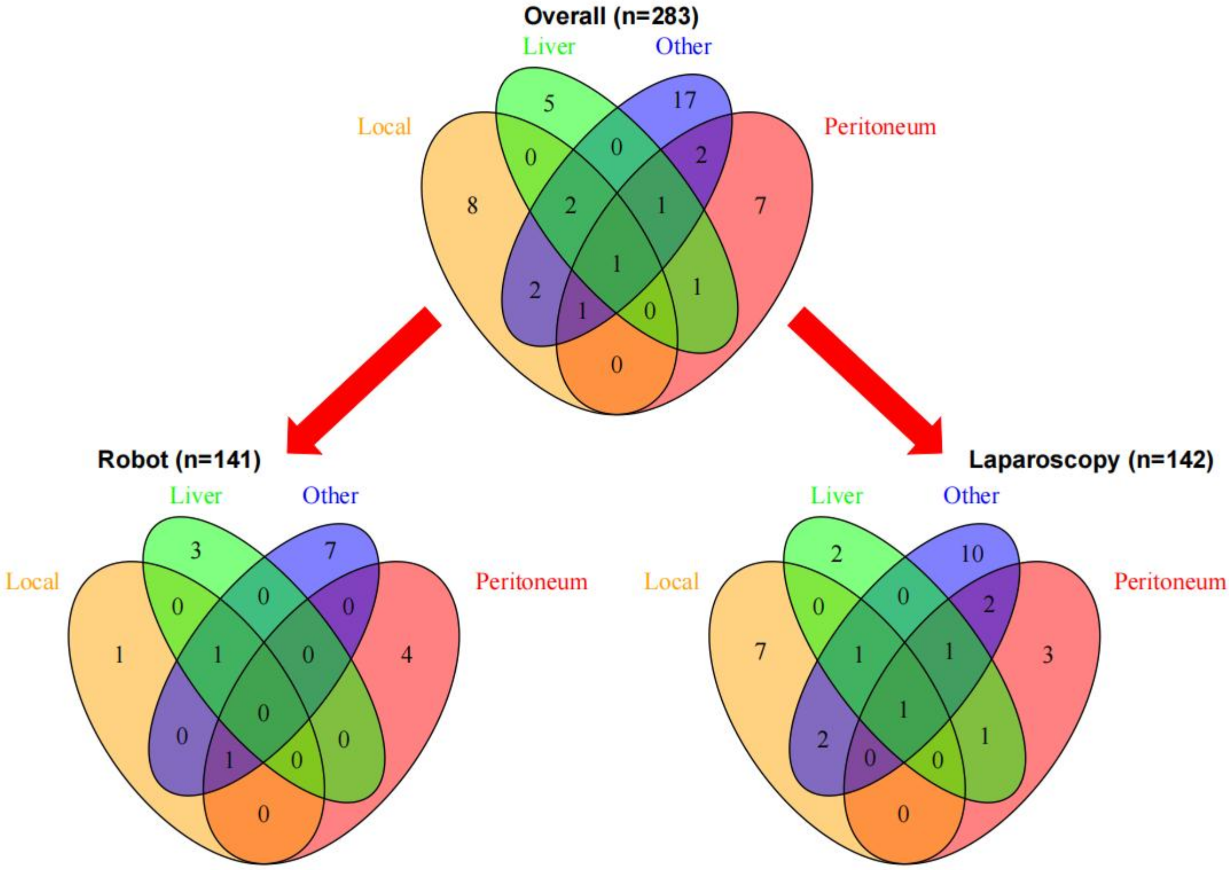

Supplementary Figure 4: Recurrence patterns

Supplementary Note 1

Data Management and Sharing Plan of Fujian Medical University Union Hospital

1. Purpose

This policy aims to ensure that the hospital's data management and sharing practices comply with relevant regulations, protect patient privacy, and promote effective data management and sharing.

2. Scope

This policy applies to all departments and personnel within the hospital and encompasses all data related to the hospital.

3. Data Classification

The hospital's data will be categorized based on sensitivity and shareability into the following types:Sensitive Patient Data: Includes patient diagnoses, medical records, identity information, etc. Medical Research Data: Involves data related to medical research and clinical trials. Administrative Data: Covers data related to hospital operations, finances, and human resources. Public Data: Non-sensitive data that can be made publicly accessible.

4. Data Collection and Storage

The hospital will take appropriate measures to ensure secure data collection, storage, and backup. This includes encryption, access controls, and regular reviews.

5. Data Sharing

Data sharing must adhere to applicable regulations and legal requirements. When sharing data, patient or research subject consent (if required) must be obtained, and

data must be transmitted in a secure manner.

6. Data Protection and Privacy

The hospital will implement measures to ensure the privacy and security of patient data. This includes data access controls, staff training, and an incident response plan.

7. Data Management Team

The hospital will establish a data management team responsible for developing and implementing data management and sharing plans. This team will conduct regular policy reviews and updates.

8. Review and Updates

This policy will undergo periodic reviews to ensure alignment with regulations and actual needs and will be updated as necessary.

9. Compliance and Oversight

The hospital will maintain compliance and oversight of data management and sharing practices to ensure policy adherence and implementation.

10. Education and Training

The hospital will provide training on data management and sharing policy to ensure all staff members are aware of and comply with the policy. Please note that this is just a sample template, and specific policy content and requirements may vary based on the hospital's specific circumstances and regulatory requirements. When creating policies, it is advisable to consult legal counsel and data protection experts to ensure policy legality and practicality.

Supplementary Note 2

Protocol and Statistical Analysis Plan (SAP)

Table of Contents

	page
**Original protocol (ver. 1.1)**	**14**
**Final protocol (ver. 2.0)**	**83**
**Summary of changes to the protocol**	**153**
**Original statistical analysis plan**	**154**
**Final statistical analysis plan**	**159**
**Summary of changes to the statistical analysis plan**	**172**

Randomized Controlled Trials on Clinical Outcomes of Robotic versus Laparoscopic Distal Gastrectomy for Gastric Cancer (FUGES-011)

Study protocol

Bidding party: Fujian Medical University Union Hospital

Principle Investigator:

Prof. Chang-Ming Huang, M.D.

Department of Gastric Surgery, Fujian Medical University Union Hospital,

Address: No. 29 Xinquan Road, Fuzhou 350001 Fujian Province, China.

Telephone: +86-591-83363366, Fax: +86-591-83363366

No. of edition: V1.1

The date of the edition: 2017.09.02

Summary

Scenario Title	**Randomized Controlled Trials on Clinical Outcomes of Robotic versus Laparoscopic Distal Gastrectomy for Gastric Cancer (FUGES-011)**
Scenario Version	V1.1
Sponsor	Chang-Ming Huang
Research Center	Fujian Medical University Union Hospital
Indications	Patients with potentially resectable gastric adenocarcinoma (cT1-4a, N0/+, M0) located in the middle and lower third of the stomach expected to undergo distal gastrectomy.
Purpose of research	To investigate the safety, feasibility and long-term outcome of robotic distal gastrectomy versus laparoscopic distal gastrectomy for gastric cancer
Research design	Single center, prospective, open-label, randomized controlled, non-inferior test
Case grouping	Group A (Study Group): Robotic distal gastrectomy Group (RDG group)<br>Group B (Control Group): Laparoscopic distal gastrectomy Group (LDG group)
The basis for determining the sample size	This study is a non-inferior test (bilateral), whose primary outcome measure is 3-year disease free survival. According to the previous study results and related literature reports, the 3-year DFS rate for the LDG group was 82.3%. According to an α of 0.025, a power of 90%, and a margin delta of 16%, we determined that at least 120 patients should be included each group. Considering an expected dropout rate of 20%, it was determined that each group needed at least 150 patients, for a total of 300 cases.

Inclusion criteria	● Age from 18 to 75 years (not including 18 and 75 years old)<br>● Primary gastric adenocarcinoma (papillary, tubular, mucinous, signet ring cell, or poorly differentiated) confirmed pathologically by endoscopic biopsy<br>● Clinical stage tumor T1-4a (cT1-4a), N-/+, M0 at preoperative evaluation according to the American Joint Committee on Cancer (AJCC) Cancer Staging Manual Eighth Edition<br>● Expected to undergo distal gastrectomy with D1+/D2 lymph node dissction to obtain R0 resection sugicall results<br>● Performance status of 0 or 1 on Eastern Cooperative Oncology Group scale (ECOG)<br>● American Society of Anesthesiology score (ASA) class I, II, or III<br>● Written informed consent
Exclusion criteria	● Women during pregnancy or breast-feeding<br>● Severe mental disorder<br>● History of previous upper abdominal surgery (except laparoscopic cholecystectomy)<br>● History of previous gastrectomy, endoscopic mucosal resection or endoscopic submucosal dissection<br>● Multiple primary gastric cancer<br>● Enlarged or bulky regional lymph node diameter over 3cm by preoperative imaging<br>● History of other malignant disease within past five years<br>● History of previous neoadjuvant chemotherapy or radiotherapy<br>● History of unstable angina or myocardial infarction within past six months<br>● History of cerebrovascular accident within past six months<br>● History of continuous systematic administration of corticosteroids within one month<br>● Requirement of simultaneous surgery for other disease<br>● Emergency surgery due to complication (bleeding, obstruction or

	perforation) caused by gastric cancer<br>● FEV1 < 50% of predicted values
Rejection criteria	● M1 tumor confirmed intraoperatively or postoperatively: distant metastasis only found by intraoperative exploration or postoperative pathological biopsy or a positive postoperative peritoneal lavage cytology examination<br>● Patients intraoperatively/postoperatively confirmed as T4b<br>● Patients intraoperatively confirmed as unable to complete D2 lymph node dissection/R0 resection due to tumor: unable to complete R0 resection due to regional lymph node integration into a mass or surrounded with important blood vessels, which cannot be resected;<br>● Patients converted to total gastrectomy intraoperatively;<br>● Patients requiring simultaneous surgical treatment of other diseases;<br>● Sudden severe complications during the perioperative period (intolerable surgery or anesthesia), which renders it unsuitable or unfeasible to implement the study treatment protocol as scheduled;<br>● Patients confirmed to require emergency surgery by attending physicians due to changes in the patient's condition after inclusion in this study;<br>● Patients who voluntarily quit or discontinue treatment for personal reasons at any stage after inclusion in this study;<br>● Treatment implemented is proven to violate study protocol.
Intervention	● Implement robotic (group A) or laparoscopic (group B) distal gastrectomy with D1+/D2 lymphadenectomy according to the Japanese gastric caner treatment guidelines 2014 (4th Edition)
Outcome Measures	**Primary Outcome Measures**：<br>● 3-year disease free survival rate<br>**Secondary Outcome Measures**：<br>● 3-year overall survival rate<br>● 3-year recurrence pattern

	● Overall postoperative morbidity rates<br>● Intraoperative morbidity rates<br>● Overall postoperative serious morbidity rates<br>● Number of retrieved lymph nodes<br>● Noncompliance rate of lymphadenectomy<br>● Time to first ambulation<br>● Time to first flatus<br>● Time to first liquid diet<br>● Time to first soft diet<br>● Duration of postoperative hospital stay<br>● The variation of weight<br>● The variation of cholesterol<br>● The variation of album<br>● The variation of white blood cell count<br>● The variation of hemoglobin<br>● Hospitalization expenses<br>● Operation time
Statistical consideratio ns	All data analyses will be performed using the SAS statistical package (version 9.2, SAS Institute, Cary, North Carolina, USA).<br>The noninferiority analysis for the primary endpoint of 3-year disease-free survival will be conducted, while the test method of difference for other outcomes. All the statistical tests were tested by two sides. A p-value <0.05 is considered statistically significant. The confidence interval of the parameters is estimated with a 95% confidence interval. Baseline data and validity analyses will be conducted on a modified intent-to-treat (MITT) basis, and the primary endpoint will also be analyzed on a per-protocol (PP) basis, with the MITT analysis results prevailing. SAP analysis is used for safety assessment, and this study does not fill in missing values. Normally distributed continuous variables will be presented as mean and standard deviation and compared using the t-test if normally distributed, or as median and interquartile range and compared using the Wilcoxon rank-sum test if non-normally distributed; while categorical data will be

	presented as number and percentages and compared using the Pearson χ2 test or the Fisher exact test, as appropriate. Survival data will be analyzed using the Kaplan-Meier method and Cox's proportional hazards model. Sensitivity analysis is used for extreme outlier data. The central effect analysis and subgroup analysis are conducted according to the specific situation. Interim analysis will not be conducted in this study.

1. Research background

In the worldwide, the incidence of gastric cancer is the fourth most common malignant tumor, and the second leading cause of cancer-related death. Although the incidence of gastric cancer has a downward trend in western countries, it still maintains a high level in East Asia. Radical gastrectomy is the only way to cure gastric cancer. In China, Japan, Korea and other East Asian countries, the primary lesions of gastric cancer are mostly located in the middle and lower third of the stomach [1]. Previous studies have shown that if the proximal resection margin is far enough, the long-term oncological effect of total gastrectomy and distal gastrectomy is equivalent. However, the quality of life of patients after distal gastrectomy is higher than those after total gastrectomy [2,3]. Therefore, distal gastrectomy is the most widely used surgical approach of gastrectomy.

Since the first laparoscopic gastrectomy was reported by Kitano et al [4] in 1994, it has been widely recognized internationally during the recent 20 years. A mount of randomize controlled trials have confirmed that laparoscopic gastrectomy has the advantages of fast recovery and less complications when compare with open gastrectomy. Moreover, the long-term survival of laparoscopic radical gastrectomy was comparable with laparotomy [11-13]. In early gastric cancer, laparoscopic distal gastrectomy has become a standard surgical approach. In addition, it has also been reported that laparoscopic radical gastrectomy is also feasible in advanced gastric cancer [14]. Development has taken place in the field of laparoscopic gastrectomy these decades, however, the traditional laparoscopic surgery has some limitations in fine steps, visual field and so on.

Because of the limited motion of laparoscopic instruments, poor visual field and, two-dimensional plane without spatial sense, it may cause vascular bleeding when perforoming lymph node dissection, due to the complex anatomical structure and compact proximity of blood vessels around the stomach. Additionally, as reported, both operation time and the learning curve are long. Especially in the patients with obesity, large anterior and posterior diameter and small costal arch angle, the difficulty will be more protruding and the laparoscopic operation will be seriously affected in the deep and narrow abdominal space.

In order to overcome the limitations of laparoscopic maneuvers, Da Vinci robotic system emerged. As an advanced laparoscopic system, robot solves many shortcomings of conventional laparoscopy with its unique advantages which are mainly reflected as following: (1) High-definition three-dimensional magnification imaging can better display small anatomical structures. It is easier to expose perigastric vessels and reduce the difficulty of lymph node dissection and the amount of intraoperative blood loss. (2) The simulated "wrist" with 7 degrees of freedom greatly improves the flexibility, especially in the difficult suture operation.

In 1997, Cadiere successfully completed the robot-assisted cholecystectomy firstly [15]. Nowadays, robotic surgery system has been widely used in the fields of urology, hepatobiliary and cardiovascular surgery and gynecology [16-19]. In the field of gastrectomy, Hashizume et al. [20] reported robotic gastrectomy for the first time in 2002. Since then, more and more reports about the safety and feasibility of robotic surgery system in the treatment of gastric cancer, especially in Asia. Liu et al. [21] conducted a meta analysis combined the results of 16 studies showing that compared with laparoscopic surgery, robotic surgery can achieve radical resection of gastric cancer, and has the advantages of less blood loss and more lymph nodes retrieved. Most studies, however, are still retrospective, and no prospective randomized controlled trial on robotic gastrectomy was reported so far. In addition, due to the high cost of the robotic surgery system, robotic gastrectomy can only be performed in high-volume hospitals, and the benefit for patients is still controversial. Kim et al. [22] reported a prospective non-randomized controlled study in 11 centers in 2015. The results showed that although robotic gastrectomy reduce the intraoperative blood loss, it takes longer operation time with higher cost and the short-term outcomes of robotic gastrectomy are not superior to traditional laparoscopic gastrectomy. In addition, that study is a non-randomized controlled trial, and there is a deviation in the baseline of patients in both arms. For example, patients in the laparoscopic group were less likely to undergo D2 lymph node dissection with earlier tumor stage. And most of the participating surgeons were experts in laparoscopic gastrectomy but were less experienced in robotic gastrectomy (the median number of robotic gastrectomy performed by the participating surgeons annually was only 5).

Therefore, based on the mature technology of traditional laparoscopic and

robotic gastrectomy, this prospective randomized controlled trial conducted at a simultaneous, large-scale center focused on patients with potentially resectable gastric adenocarcinoma (cT1-4a, N0/+, M0) located in the middle and lower third of the stomach to evaluate the short- and long-term effect of robotic distal gastrectomy.

2. Objective

The purpose of the randomized controlled trial is to investigate the safety, feasibility and long-term outcome of robotic distal gastrectomy versus laparoscopic distal gastrectomy for gastric cancer.

3. Research design

Single center, prospective, open-label, parallel assignment, randomized controlled.

3.1 Single center

Department of gastric surgery in Fujian Medical University Union Hospital

3.2 Case group

Group A (Study Group): Robotic distal gastrectomy Group (RDG group)

Group B (Control Group): Laparoscopic distal gastrectomy Group (LDG group)

3.3 Estimate Sample Size

This study is a non-inferior test (bilateral), whose primary outcome measure is 3-year disease free survival. According to the previous study results and related literature reports, the projected 3-year DFS rate for the LDG group was 82.3%. Based on an α of 0.025, a power of 90%, and a margin delta of 16%, we determined that at least 120 patients should be included each group. Considering an expected dropout rate of 20%, a total of 300 patients were needed.

3.4 Blind method： This research adopts an open design

3.5 Research cycle

Estimated enrollment cycle: complete enrollment within 2 years

Follow-up period: begin at the enrollment of the first case and end 3 years after the enrollment of the last case.

Estimated time: 2017.09-2019.09 （to complete enrollment） - 2022.09 （to

complete follow-up)

3.6 Randomization

SAS 9.2 program was used to generate serial numbers from 001 to 300 that corresponds to the intervention assignment. Before the surgery, the data manager extracted the numbers and then randomly assigned patients in a 1:1 ratio to either the LDG group or the RDG group. Written informed consent was obtained from patients.

4. Study objects

All patients who meet the inclusion criteria and not conform to the exclusion criteria are qualified for this study.

4.1 Inclusion criteria

(1) Age from 18 to 75 years (not including 18 and 75 years old)

(2) Primary gastric adenocarcinoma (papillary, tubular, mucinous, signet ring cell, or poorly differentiated) confirmed pathologically by endoscopic biopsy

(3) Clinical stage tumor T1-4a (cT1-4a), N-/+, M0 at preoperative evaluation according to the American Joint Committee on Cancer (AJCC) Cancer Staging Manual Eighth Edition

(4) Expected to undergo distal gastrectomy and D1+/D2 lymph node dissection to obtain R0 surgical results.

(5) Performance status of 0 or 1 on the ECOG (Eastern Cooperative Oncology Group) scale

(6) ASA class I to III

(7) Written informed consent

4.2Exclusion criteria

(1) Women during pregnancy or breast-feeding

(2) Severe mental disorder

(3) History of previous upper abdominal surgery (except for laparoscopic cholecystectomy)

(4) History of previous gastric surgery (including ESD/EMR for gastric cancer)

(5) Multiple primary gastric cancer

(6) Enlarged or bulky regional lymph node diameter over 3cm by preoperative imaging

(7) History of other malignant disease within past five years

(8) History of previous neoadjuvant chemotherapy or radiotherapy

(9) History of unstable angina or myocardial infarction within the past six months

(10) History of cerebrovascular accident within past six months

(11) History of continuous systematic administration of corticosteroids within one month

(12) Requirement of simultaneous surgery for another disease

(13) Emergency surgery due to complications (bleeding, obstruction or perforation) caused by gastric cancer

(14) FEV1 < 50% of the predicted values

4.3 Rejection criteria

(1) M1 tumor confirmed intraoperatively or postoperatively: distant metastasis only found by intraoperative exploration or postoperative pathological biopsy or a positive postoperative peritoneal lavage cytology examination

(2) Patients intraoperatively/postoperatively confirmed as T4b

(3) Patients intraoperatively confirmed as unable to complete D2 lymph node dissection/R0 resection due to tumor: unable to complete R0 resection due to regional lymph node integration into a mass or surrounded with important blood vessels, which cannot be resected;

(4) Patients converted to total gastrectomy intraoperatively;

(5) Patients requiring simultaneous surgical treatment of other diseases;

(6) Sudden severe complications during the perioperative period (intolerable surgery or anesthesia), which renders it unsuitable or unfeasible to implement the study treatment protocol as scheduled;

(7) Patients confirmed to require emergency surgery by attending physicians due to changes in the patient's condition after inclusion in this study;

(8) Patients who voluntarily quit or discontinue treatment for personal reasons at any stage after inclusion in this study;

(9) Treatment implemented is proven to violate study protocol.

4.4 Case screening

(1) When Patients admitted to hospital should meet the following criteria: age between 18 and 75 years old; performance status of 0 or 1 on the ECOG scale;

none-pregnant or no lactating women; not suffering from a severe mental disorder; no history of previous upper abdominal surgery (except for laparoscopic cholecystectomy); no history of previous gastric surgery (including ESD/EMR for gastric cancer); no history of other malignant disease within the past five years; no history of unstable angina or myocardial infarction within the past six months; no history of continuous systematic administration of corticosteroids within one month; no requirement of simultaneous surgery for another disease; FEV1≥50% of the predicted values; no history of a cerebrovascular accident within the past six months.

(2) Endoscopic examination of the primary lesion in the patient (recommended endoscopic ultrasound endoscopy, EUS) and histopathological biopsy showed gastric adenocarcinoma (papillary adenocarcinoma [pap], tubular adenocarcinoma [tub], mucinous adenocarcinoma [muc], signet ring cell carcinoma [sig], and poorly differentiated adenocarcinoma [por]). Total abdominal CT was performed on the patient, and no enlarged lymph nodes (maximum diameter ≥ 3 cm) were found in the periplasmic area, including significant enlargement or merging of the No. 10 lymph nodes into a group or local invasion/distance metastasis. No obvious tumor infiltration was found in the spleen and spleen vessels.

(3) Patient is explicitly diagnosed with middle and/or lower third gastric cancer, has a preoperative staging assessment of T1-4a, N0-3, M0 and is expected to undergo distal gastrectomy with D1+/D2 lymph node dissection to obtain R0 surgical results.

(4) Patients do not require neoadjuvant chemoradiotherapy or chemotherapy and the attending doctor does not recommend that they receive neoadjuvant chemoradiotherapy or chemotherapy.

(5) ASA class I to III.

(6) No requirement for emergency surgery.

(7) At this point the patient becomes a potential selected case and enters the 9.1 case selection procedure.

5. Outcome Measures

5.1 Primary Outcome Measures

- 3-year disease free survival rate

5.2 Secondary Outcome Measures

- 3-year overall survival rate
- 3-year recurrence pattern
- overall postoperative morbidity rates
- intraoperative morbidity rates
- overall postoperative serious morbidity rates
- number of retrieved lymph nodes
- the noncompliance rate of lymphadenectomy
- time to first ambulation
- time to first flatus
- time to first liquid diet
- time to first soft diet
- duration of postoperative hospital stay
- the variation of weight
- the variation of cholesterol
- the variation of album
- the variation of white blood cell count
- the variation of hemoglobin
- hospitalization expenses
- operation time

6. Diagnostic criteria for this study

(1) The AJCC-8th TNM tumor staging system will be used for this study.

(2) Diagnostic criteria and classification of gastric cancer: According to the histopathological international diagnostic criteria, classification will be divided into papillary adenocarcinoma (pap), tubular adenocarcinoma (tub), mucinous adenocarcinoma (muc), signet ring cell carcinoma (sig), and poorly differentiated adenocarcinoma (por).

- The definition of middle and lower third gastric cancer:

Acoording to Japanese classification of gastric carcinoma (4rd English edition), the stomach is anatomically divided into three portions, the upper (U), middle (M), and lower (L) parts, by the lines connecting the trisected points on the lesser and greater curvatures (Fig. 1). Middle and lower third gastric cancer is described as the center of tumor located in the middle and lower third part of stomach, including M, L, ML.

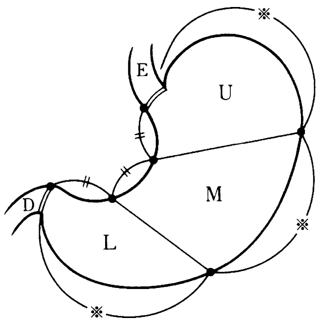

Fig. 1. The three portions of the stomach. *U* upper third, *M* middle third, *L* lower third, *E* esophagus, *D* duodenum

7. Qualifications of the participated Surgeons

7.1 Basic principle

All candidate surgeons in our study met the following criteria:

Performed at least 300 laparoscopic radical gastrectomies and at least 50 robotic radical gastrectomies.

Pass the blind surgical video examination.

7.2 Checklist for determination of success about D2 lymphadenectomy

Scoring Method for D2 Lymph Node Dissection	Complete	Incomplete	None
	10	5	0

1. Properly full omentectomy	□	□	□
2. Ligation of left gastroepiploic artery at origin	□	□	□
3. Ligation of right gastroepiploic artery at origin	□	□	□
4. Full exposure of common hepatic artery	□	□	□
5. Ligation of right gastric artery at origin	□	□	□
6. Exposure of portal vein	□	□	□
7. Exposure of splenic artery to branch of posterior gastric artery	□	□	□
8. Identification of splenic vein	□	□	□
9. Ligation of left gastric artery at origin	□	□	□
10. Exposure of gastroesophageal junction	□	□	□

1. Properly full omentectomy

    a. Omentectomy was performed close to transverse colon

    b. Omentectomy was performed from hepatic flexure to splenic flexure

    c. Anterior layer of transverse colonic mesentery and pancreatic anterior peritoneum was dissected.

2. Ligation of left gastroepiploic artery at origin

3. Ligation of right gastroepiploic artery at origin

4. Full exposure of common hepatic artery

    a. More than half of anterior part in the common hepatic artery were exposed.

5. Ligation of right gastric artery at origin

6. Exposure of portal vein

7. Exposure of splenic artery to branch of posterior gastric artery

a. More than half of anterior part in splenic artery was exposed.

b. Splenic artery was exposed from celiac trunk to posterior gastric artery

8. Identification of splenic vein

9. Ligation of left gastric artery at origin

10. Exposure of gastroesophageal junction

a. Anterior and right side of the abdominal esophagus were exposed.

- D2 lymphadenectomy was accepted if all randomly assigned three investigators rated 85 points and more regarding checklists in unedited video review.

8. End point and definition of related result determination

8.1 Disease-free survival

Disease-free survival is calculated from the day of surgery to the day of recurrence or death (when the specific date of recurrence of the tumor is unknown, the ending point is the date of death due to tumor causes）. In the event that neither death nor recurrence of the tumor are observed, the end point is the final date that a patient is confirmed as relapse-free.（The final date of DFS: the last date of the outpatient visit day or the date of acceptance of the examination）.（Follow-up cycle and required examinations are shown in the follow-up process 9.5.3）

8.2 Overall survival time

The overall survival is calculated from the day of surgery until death or until the final follow-up date, whichever occurs first. For survival cases, the end point is the last date that survival was confirmed. If loss to follow-up occurred, the end point is the final date that survival could be confirmed.

8.3 Definition of recurrence and recurrence date

The following situations are regarded as "recurrence" and should be recorded as

the evidence of "recurrence" in the CRF.

(1) Recurrence identified by any one image examination (X-ray, ultrasound, CT, MRI, PET-CT, endoscope, etc.) and, if there are a variety of imaging examinations, results without contradiction determined "recurrence". The earliest date that the recurrence is found is defined as the "recurrence date".

(2) For cases that lack the use of imaging or a pathological diagnosis, the date we diagnose the occurrence of clinical recurrence based on clinical history and physical examination is defined as the “recurrence date”.

(3) For cases without imaging or clinical diagnosis but with a cytology or tissue biopsy pathological diagnosis of recurrence, the earliest date confirmed by cytology or biopsy pathology is considered the "recurrence date".

(4) A rise in CEA or other associated tumor markers alone could not be diagnosed as a relapse.

8.4 Incidence of surgical complications

8.4.1 Incidence of intraoperative complications

The number of all patients treated with surgery as the denominator and the number of the patients with any intraoperative complications as the numerator are used to calculate the proportions. The criteria for the intraoperative complications refer to the descriptions of intraoperative complications in the observation project (in 9.3.3).

8.4.2 Incidence of postoperative complications

The number of all patients treated with surgery as the denominator and the number of the patients with any postoperative complications as the numerator are used to calculate the proportions.

Incidence of overall postoperative complications: The postoperative complication criteria refer to short-term complications after surgery in the postoperative observation project (see 9.4.5). The time is defined as within 30th after surgery, or the first discharge time if the days of hospital stay more than 30 days.

Incidence of postoperative major complications: The standard for postoperative major complications refers to the short-term complications in the postoperative observation project (see 9.4.5). According to the Clavien–dindo grade, IIIA level and above for serious complications, and when multiple complications occur

simultaneously, the highest ranked complication is the subject.

8.4.3 Mortality

- The number of all the patients receiving surgery as the denominator and the number of the patients in any of the following situations as the numerator are used to calculate proportions. This proportion indicated the operative mortality ratio.
- Situations: patients whose death was identified according to documented intraoperative observation items, including patients who die within 30 days after the surgery (including 30 days) regardless of the causality between the death and the surgery, and patients who die more than 30 days after the surgery (whose death is proved to have a direct causal relationship with the first operation).

8.5 Number of lymph node dissection

The sum of retrieved lymph nodes in each station.

8.6 Determination of surgical outcomes

8.6.1 Operative time: from skin incision to the skin being sutured

8.6.2 Postoperative recovery indexes

8.6.2.1 Time to ambulation, flatus, recovery of liquid diet and semi-liquid diet.

- During the day of surgery to the first discharge, the initial time to ambulation, flatus, liquid diet and semi-liquid diet during the postoperative hospitalization is recorded by hour.
- Flatus on the operation day should be excluded.
- If flatus or resumption of liquid and semi-liquid diet does not occur before hospital discharge, the discharge time should be recorded as the corresponding time.
- The initial time to ambulation, flatus, liquid diet and semi-liquid diet should be recorded according to patients' reports.

8.6.2.2 The maximum temperature

The highest value of body temperature measured at least 3 times a day from the first day to the eighth day after operation is documented.

8.6.3 Laparoscopic / Robotic surgery completion ratio

The number of all patients treated with laparoscopic/robotic surgery as the denominator and the number of the patients without conversion to laparotomy as the numerator are used to calculate the ratio.

8.6.4 Percentage of conversion to laparotomy

Among all the patients who underwent surgery, the number of patients planning to receive a laparoscopic surgery per protocol is used as the denominator, while the number of the patients who receive a conversion to open surgery is considered the numerator. The proportion calculated is regarded as the rate of transfer laparotomies. In this study, if the length of the auxiliary incision is more than 10 cm, it is considered a conversion to open surgery.

9. Standard operating procedures (SOP)

9.1 Case selection

9.1.1 Selection assessment items

Clinical examination data of patients conducted from hospital admission to enrollment into this study (time period is usually 2 weeks) will be considered baseline data, and must include:

(1) Systemic status: ECOG score, height, weight

(2) Peripheral venous blood: Hb、RBC、WBC、LYM、NEU、NEU%、PLT、MONO

(3) Blood biochemistry: albumin, prealbumin, total bilirubin, indirect bilirubin, direct bilirubin, AST, ALT, creatinine, urea nitrogen, Total cholesterol, triglycerides, fasting glucose, potassium, sodium, chlorine, calcium

(4) Serum tumor markers: CEA、CA19-9、CA72-4、CA12-5、AFP

(5) Full abdominal (slice thickness of 10mm or less, in case of allergy to the contrast agent, CT horizontal scanning is allowed only)

(6) Upper gastrointestinal endoscopic ultrasonography (EUS) and biopsy, if no EUS, select ordinary upper gastrointestinal endoscopy and biopsy instead

(7) Chest X-ray (AP and lateral views): cardiopulmonary conditions

(8) Resting 12-lead ECG

(9) Respiratory function tests: FEV1, FVC

9.1.2 Selection application

For cases that meet all inclusion criteria and none of the exclusion criteria, talk to patients and their families and sign informed consent. Application and confirmation of eligibility should be completed preoperatively; postoperative applications will not be

accepted.

9.2 Preoperative management

After the eligibility is obtained, surgery should be performed within two weeks (including the 14th day)

- In case of any deterioration of the clinical conditions from the selection time to the expected day of surgery, whether to undergo an elective surgery as planned should be decided in accordance with the judgment of the doctor in charge; if an emergency surgery is required, the case should be withdrawn from PP set according to 4.3 Withdrawal Criteria;
- For patients with nutritional risks, preoperative enteral/parenteral nutritional support is allowed
- For elderly, smokers, high-risk patients with diabetes, obesity and chronic cardiovascular/cerebrovascular or thromboembolic past history, among others, perioperative low-molecular-weight heparin prophylaxis, lower-limb antithrombotic massage, active lower limb massage, training in respiratory function and other preventive measures are recommended. For other potentially high-risk complications not specified in this study protocol, the doctor in charge of each research participating center can decide on the most appropriate approach according to clinical practice and specific needs of each center and should record it in the CRF.
- For the operative approach of the surgeries in this study is distal gastrectomy and D1+/D2 lymphadenectomy according to the Japanese gastric cancer treatment guidelines 2014 (4th Edition), while reconstruction method should be selected by the doctor in charge according to his/her experience and the specific intraoperative circumstances.
- Preoperative fasting and water deprivation and other before-anesthesia requirements on patients should follow the conventional anesthesia program of each research participating center, which is not specified in this study.
- For prophylactic antibiotics, the first intravenous infusion should begin 30 minutes prior to surgery. It is recommended to select a second-generation cephalosporin (there are no provisions on specific brands in this study); the preparation, concentration and infusion rate should comply with routine

practice; and prophylaxis should not exceed postoperative three days at a frequency of one infusion every 12 hours. If patient is allergic to cephalosporins (including history of allergy or allergy after cephalosporin administration), other types of antibiotics are allowed according to the specific clinical situation and when used over the same time period mentioned.

- Patient data to be collected during the preoperative period also includes CRP

9.3 Standardization of surgical practice

9.3.1 Handling practices followed by both groups

9.3.1.1 Anesthesia

The operation is to be carried out with endotracheal intubation under general anesthesia; whether epidural assisted anesthesia is applied or not is left at the discretion of the anesthetist and is not specified in this study protocol.

9.3.1.2 Regulations on obtaining sample of the peritoneal lavage

After entering the abdominal cavity, take peritoneal lavage cytology specimens for postoperative examination immediately. More specifically, if ascites is found, sampling the ascites directly. When there is no ascites, 100ml of physiological saline is slowly injected into the abdominal cavity, and then collect samples from Douglas fossa for inspection.

9.3.1.3 Intraoperative exploration

Explore the abdominal cavity for any hepatic, peritoneal, mesenteric, or pelvic metastases and gastric serosal invasion

9.3.1.4 Regulations on the extent of the gastrectomy

Distal gastrectomy was performed on the premise that oncological principles first can be satisfied.

9.3.1.5 Regulations on digestive tract reconstruction

The digestive tract reconstruction method is to be determined by the surgeon according to his/her own experience and the intraoperative situation. If instrumental anastomosis is used, whether the manual reinforced stitching is to be performed or not on anastomotic stoma is determined by the surgeon and not specified in this study protocol.

9.3.1.6 Regulations on lymph node dissection

Performing D1+/D2 lymphadenectomy according to Japanese gastric cancer guidelines 2014(4th Edition).

9.3.1.7 Regulations on Omentum resection

According to surgeon's experience and actual needs and are not specified in this study protocol

9.3.1.8 Regulations on surgery-related equipment and instruments

Energy equipment, vascular ligation method, digestive tract cutting closure, and digestive tract reconstruction instruments are determined by the surgeon in charge of the operation according to his/her own experience and actual needs and are not specified in this study protocol.

9.3.1.9 Regulations on gastric canal and peritoneal drainage tube

Whether an indwelling gastric canal or peritoneal drainage tube is left or not after operation is determined by the surgeon in charge of the research participating center according to his/her own experience and actual needs and are not specified in this study protocol.

9.3.1.10 Regulations on simultaneous surgery for other disease

If any other system/organ disease is found during surgery, the responsible surgeon and the consultants of relevant departments should jointly determine performance of a concurrent operation if there is such necessity. The priority of operations is determined according to clinical routine; the patients meeting Exclusion Criteria will be excluded from the PP Set.

9.3.1.11 Regulations on handling of excluded patients as identified intraoperatively

If the surgeon in charge judges and determines that the patient undergoing surgery belongs to the exclusion case group, then the research approach is suspended and the surgeon will follow routine clinical practice of the research participating center to decide subsequent treatment (therapeutic decisions as to whether to excise gastric primary focus and metastases are made by the surgeon in charge); The excluded cases still need to complete data collection and follow-up and included in the analysis study (ITTP population).

9.3.1.12Regulations on imagery/photographing

A digital camera (8 million pixels at least) will be used to take pictures which shall contain the following contents (see the example below):

(1) Field of lymph node dissection (5 pictures)

Inferior pylorus region (1 picture); the right gastroepiploic arteriovenous cut site should be included.

Right-side area of the superior margin of the pancreas (1 picture); the front top of the entire common hepatic artery, the half front of the inferior proper hepatic artery and the cut site of the right gastric artery should be included.

Left-side region of the superior margin of the pancreas (1 picture); the left gastric arteriovenous cut position, celiac arterial trunk and proximal splenic artery should be included.

Right side of the cardia and lesser gastric curvature side (1 picture).

Left gastroepiploic vessel dividing position (1 picture); the cut site of the left gastroepiploic artery and vein should be included.

(2) After the skin incision is closed (1 picture, measuring scale serving as a reference object).

(3) Postoperative fresh specimens (4 pictures, measuring scale serving as a reference object); 1 picture before and 3 pictures after dissection (mark focus size; 1 picture each of distal and proximal incisional margins). After the specimen is cut open along the greater gastric curvature, a measuring scale is placed as a reference object before taking pictures to record the following items: the distance between the tumor edge and the proximal incisional margin (1 picture), the distance between the tumor edge and the distal incisional margin (1 picture), and the focus size and appearance of the mucosal face after the specimen is unfolded (1 picture).

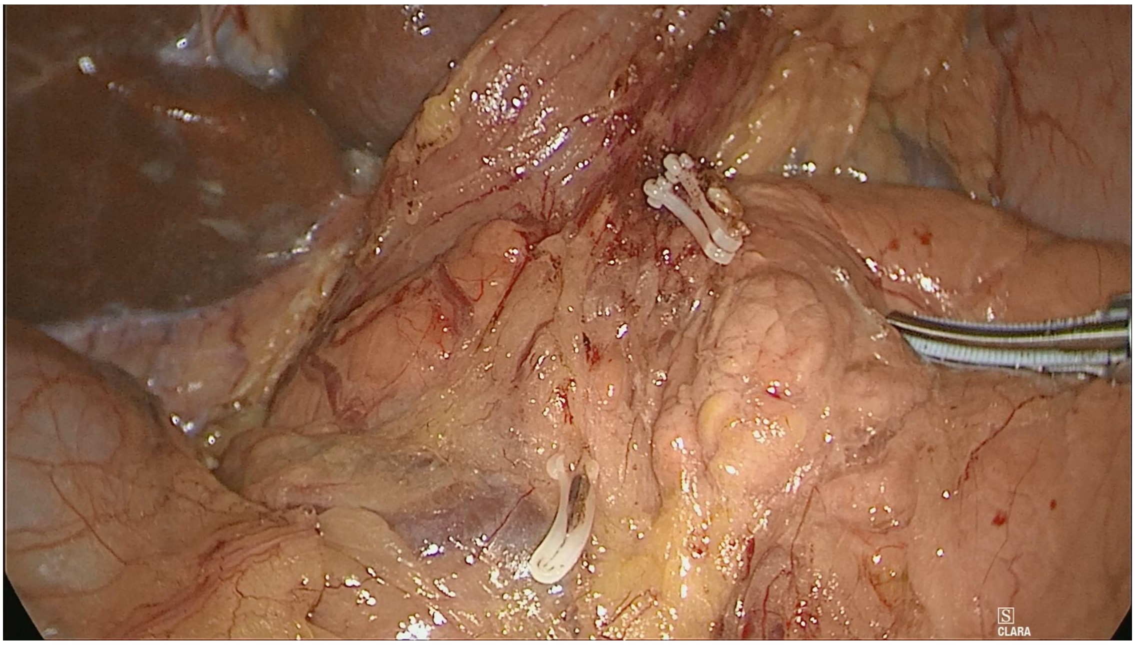

Fig. 2-1A Inferior pylorus area for laparoscopic surgery (no. 6 lymph nodes)

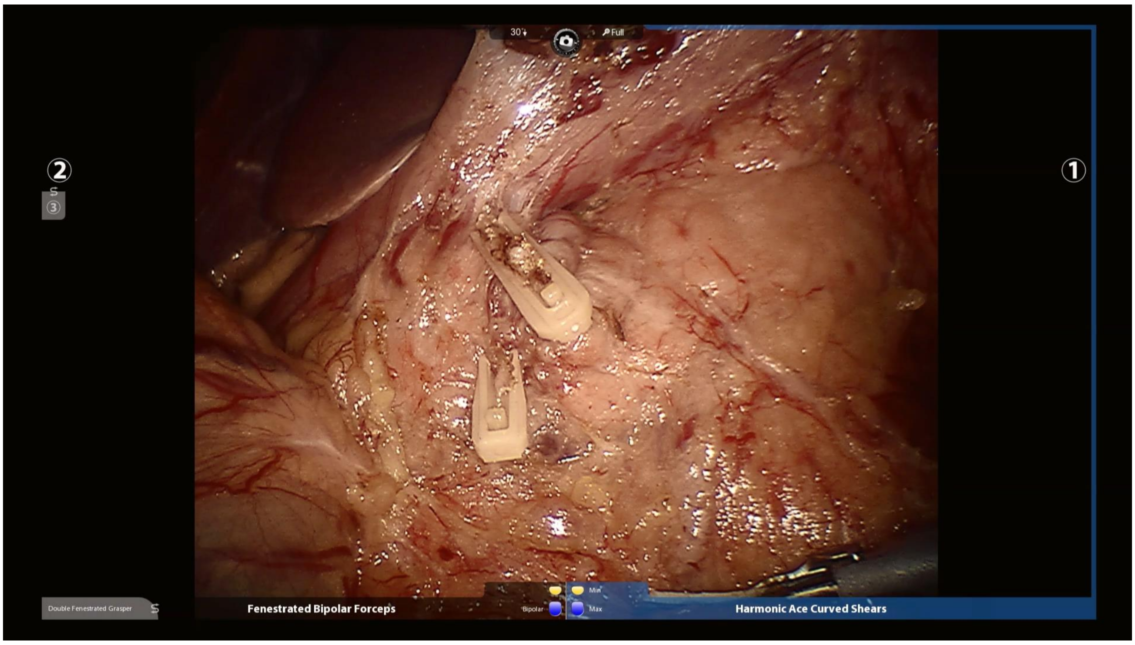

Fig. 2-1B Inferior pylorus area for robotic surgery (no. 6 lymph nodes)

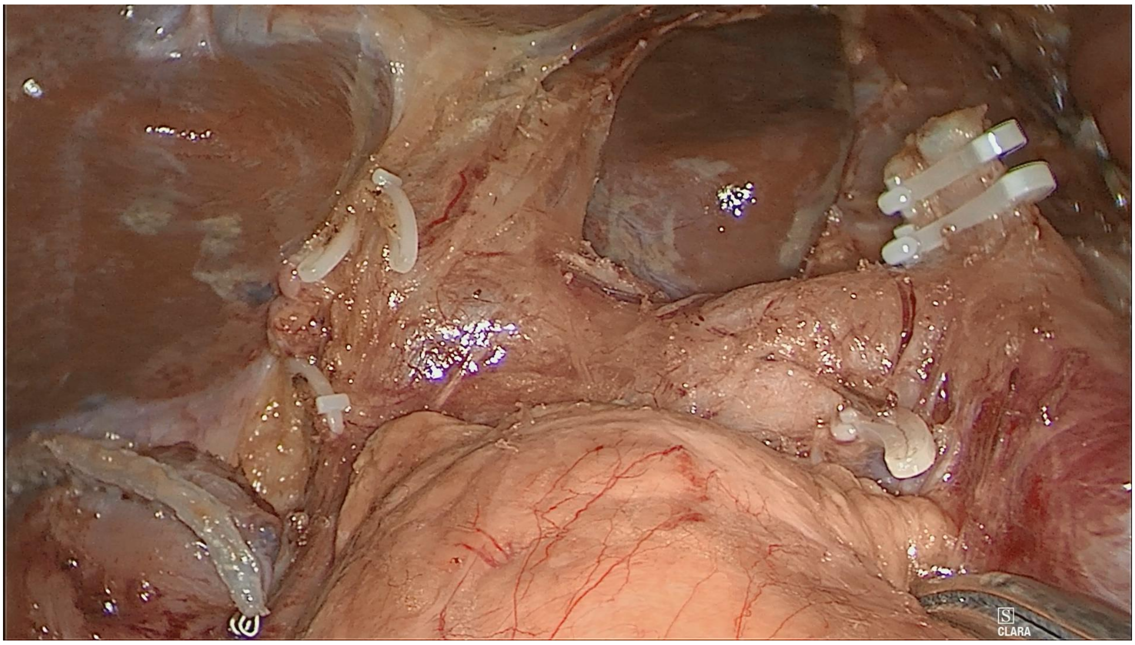

Fig. 2-2A Right-side area of the superior margin of the pancreas for laparoscopic surgery (no. 5, no. 8a and no. 12a lymph nodes)

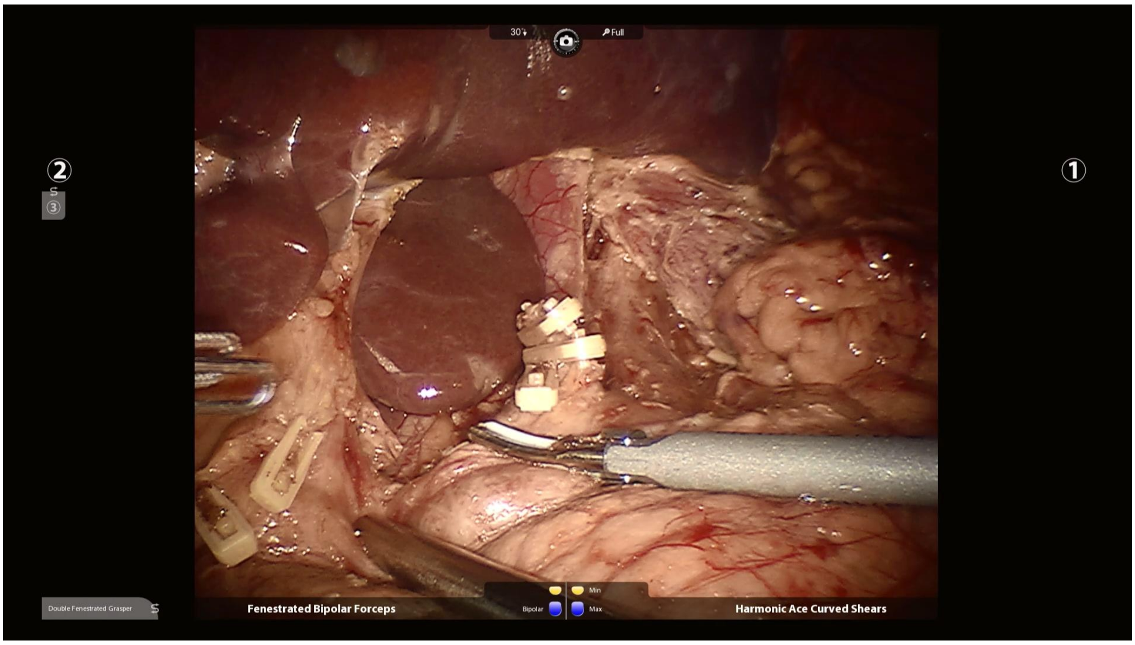

Fig. 2-2B Right-side area of the superior margin of the pancreas for robotic surgery (no. 5, no. 8a and no. 12a lymph nodes)

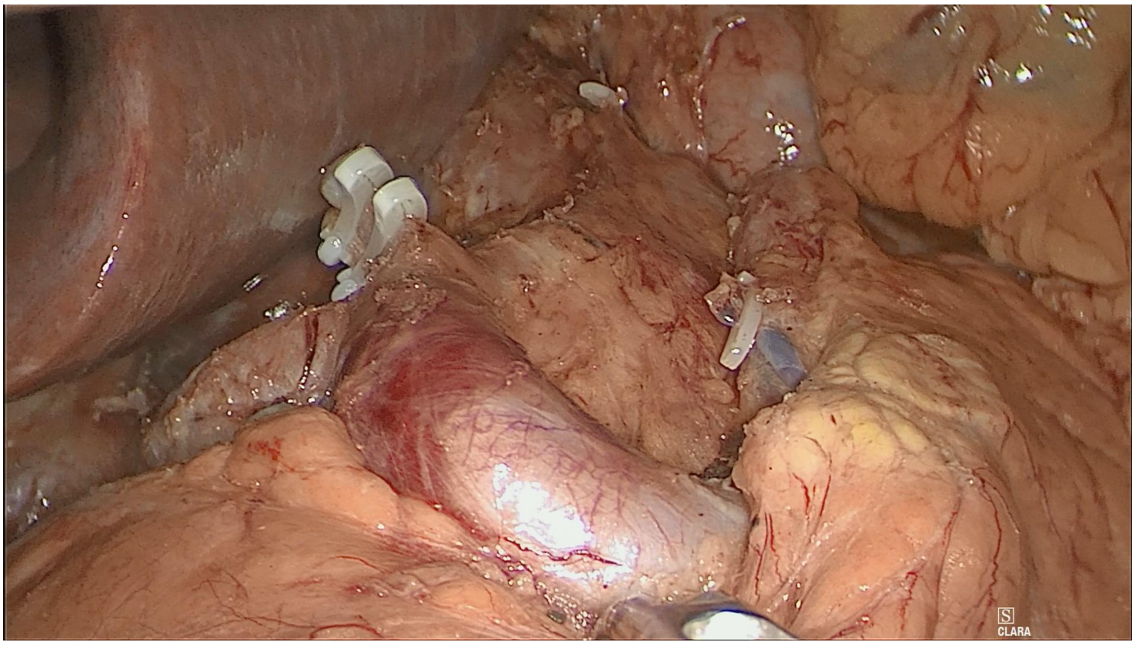

Fig. 2-3A Left-side area of the superior margin of the pancreas for laparoscopic surgery (no. 7, no. 9 and no. 11p lymph nodes)

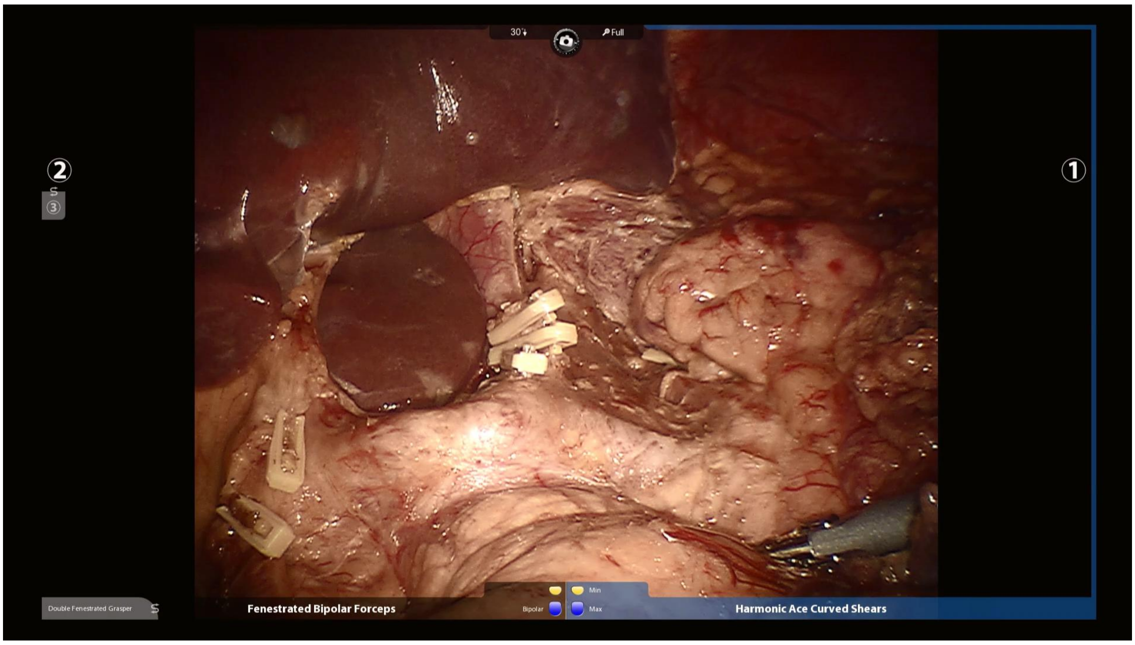

Fig. 2-3B Left-side area of the superior margin of the pancreas for robotic surgery (no. 7, no. 9 and no. 11p lymph nodes)

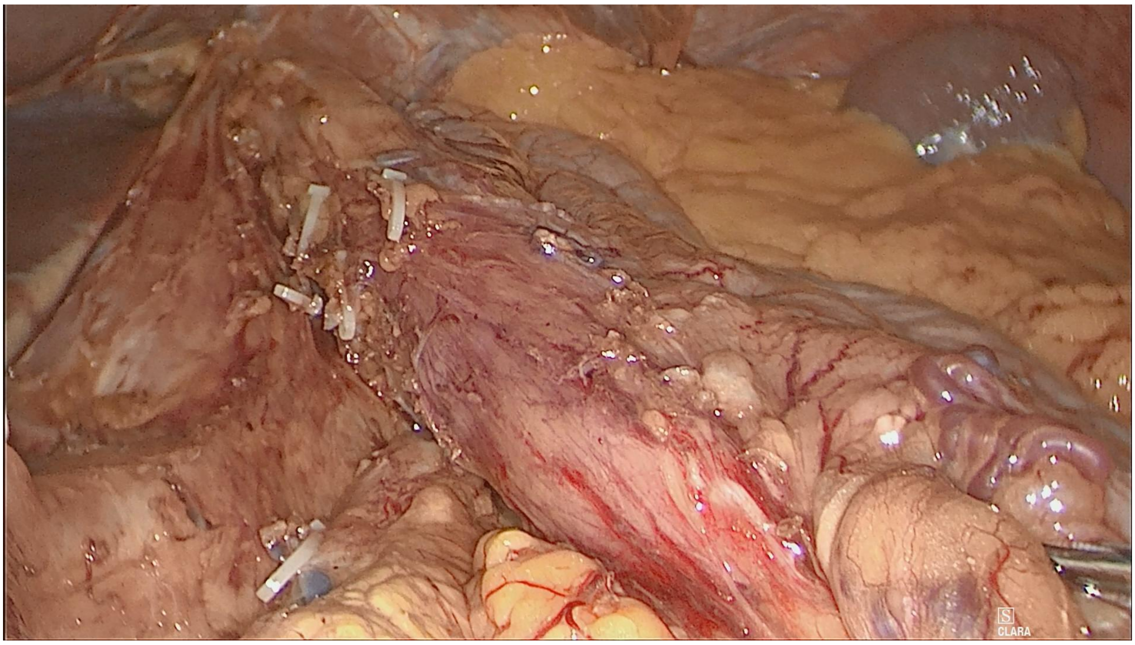

Fig. 2-4A Right side of the cardia and lesser gastric curvature side for laparoscopic surgery (the no. 1 and no. 3 lymph nodes)

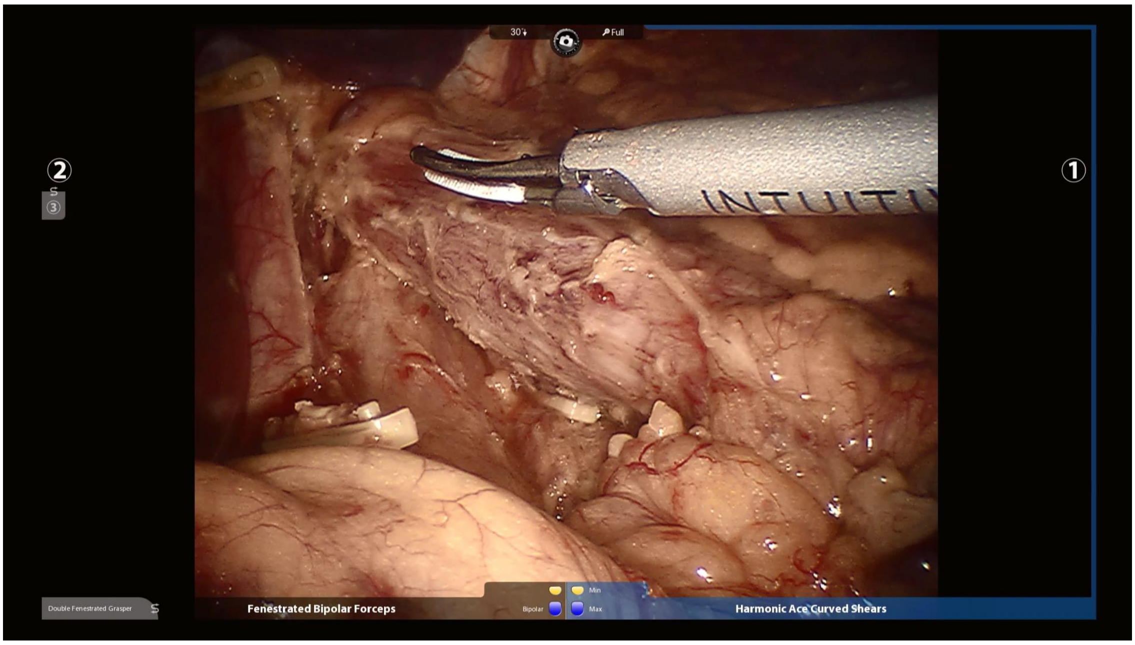

Fig. 2-4B Right side of the cardia and lesser gastric curvature side for robotic surgery (the no. 1 and no. 3 lymph nodes)

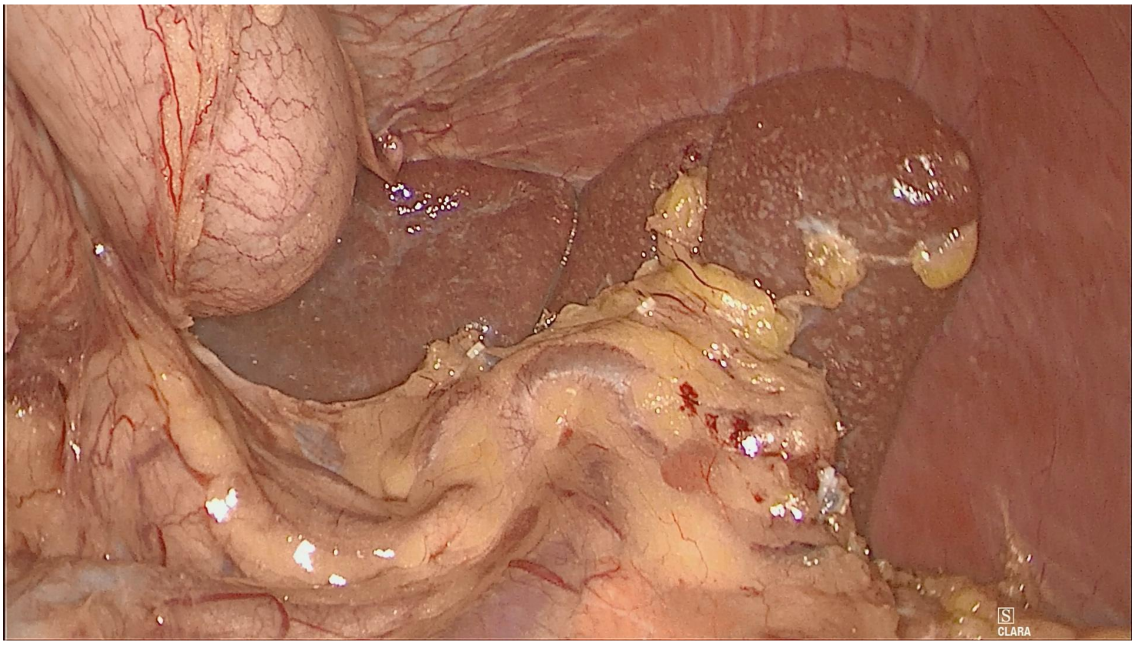

Fig. 2-5A Cut site of the left gastroepiploic vessel for laparoscopic surgery (no. 4 sb lymph nodes)

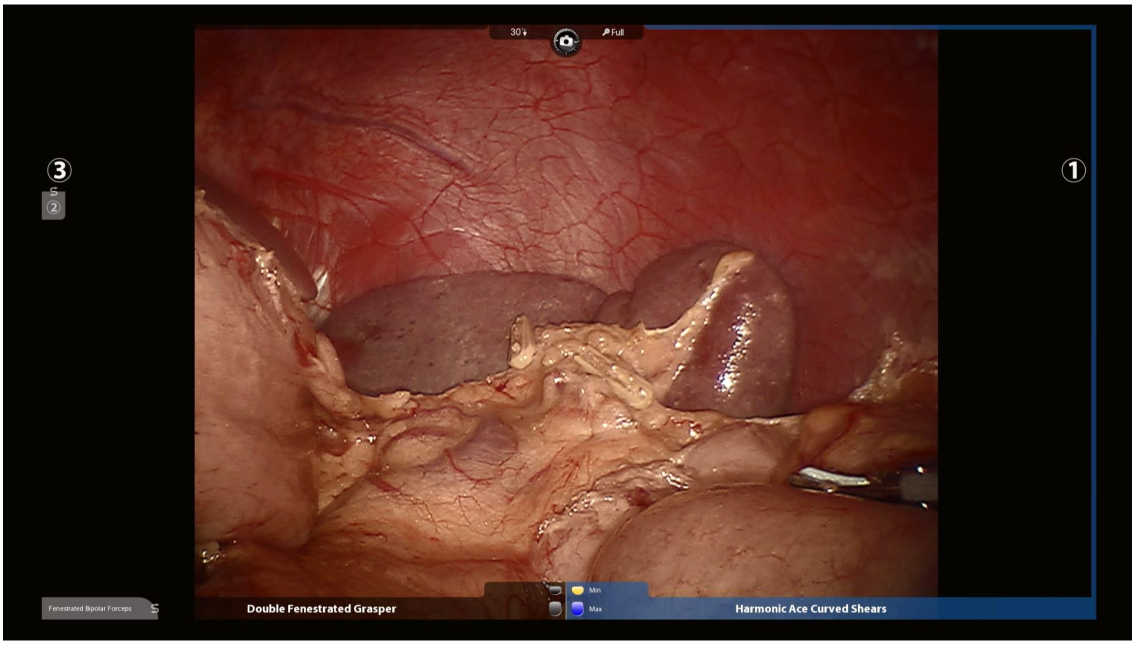

Fig. 2-5B Cut site of the left gastroepiploic vessel for laparoscopic surgery (no. 4 sb lymph nodes)

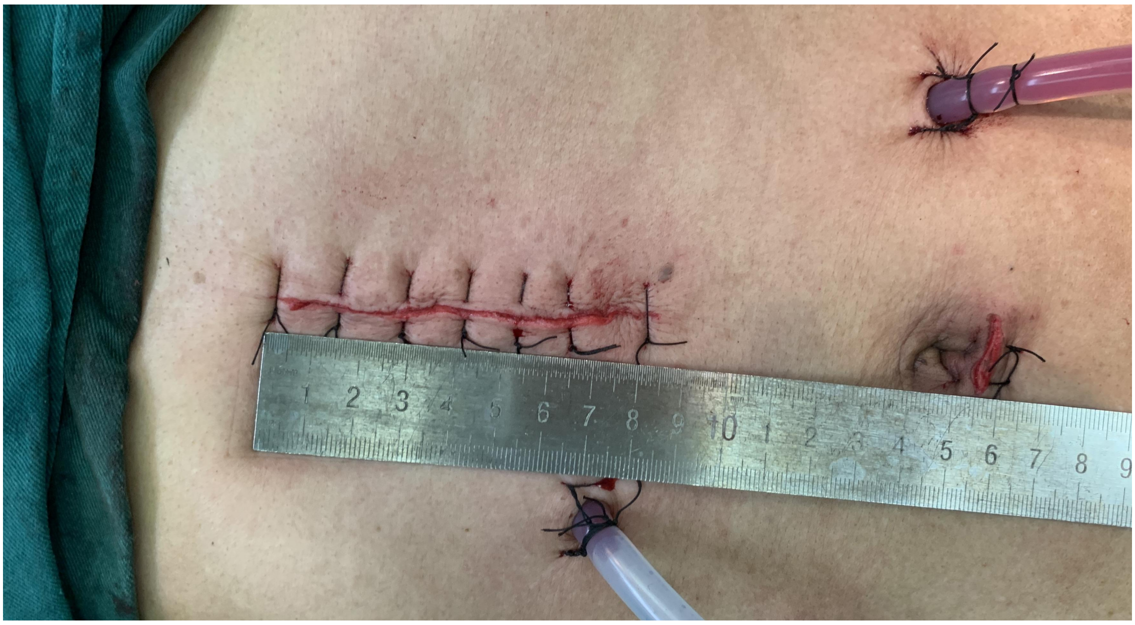

Fig. 2-6 Incision appearance (mark the incision length)

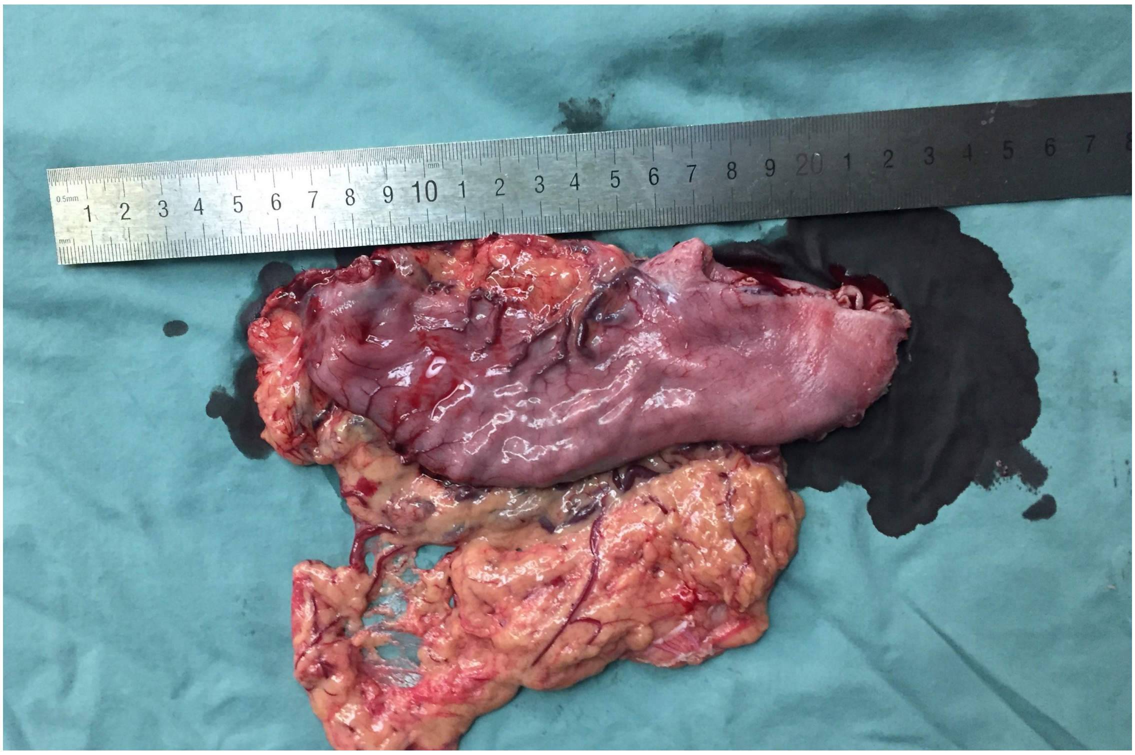

Fig. 2-7 Specimen observation (before dissection)

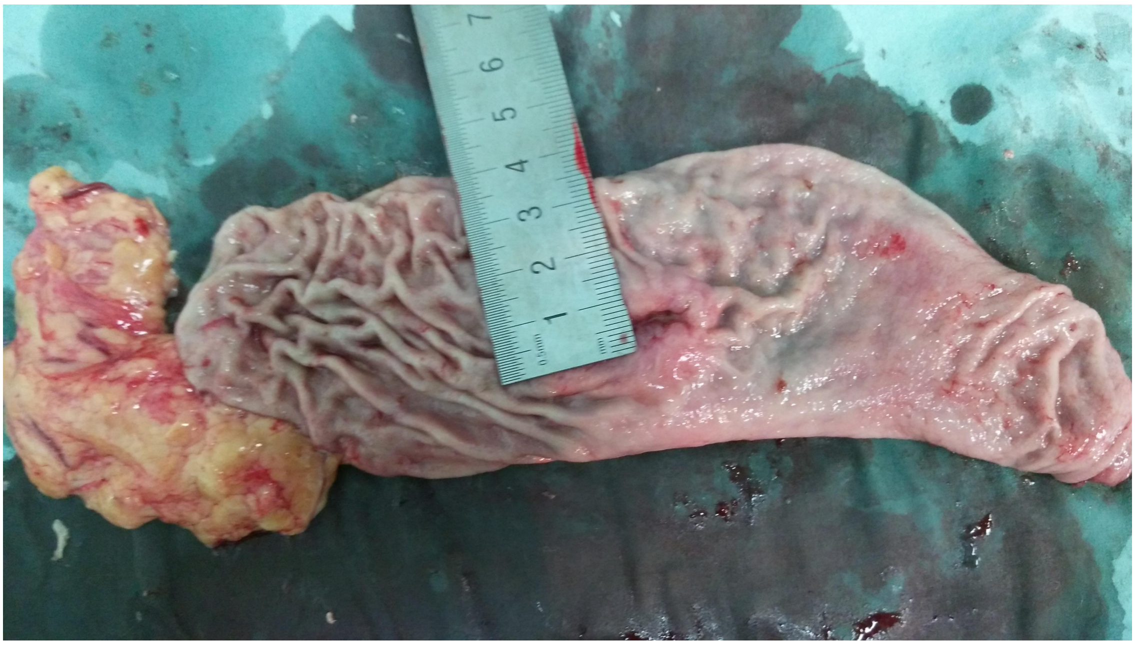

Fig. 2-8 Specimen observation (focus size; the dissection is made along the greater gastric curvature, and the focus and incisional margin on the mucosal face are observed; if the tumor is located at the greater gastric curvature, then the dissection is made along the lesser curvature)

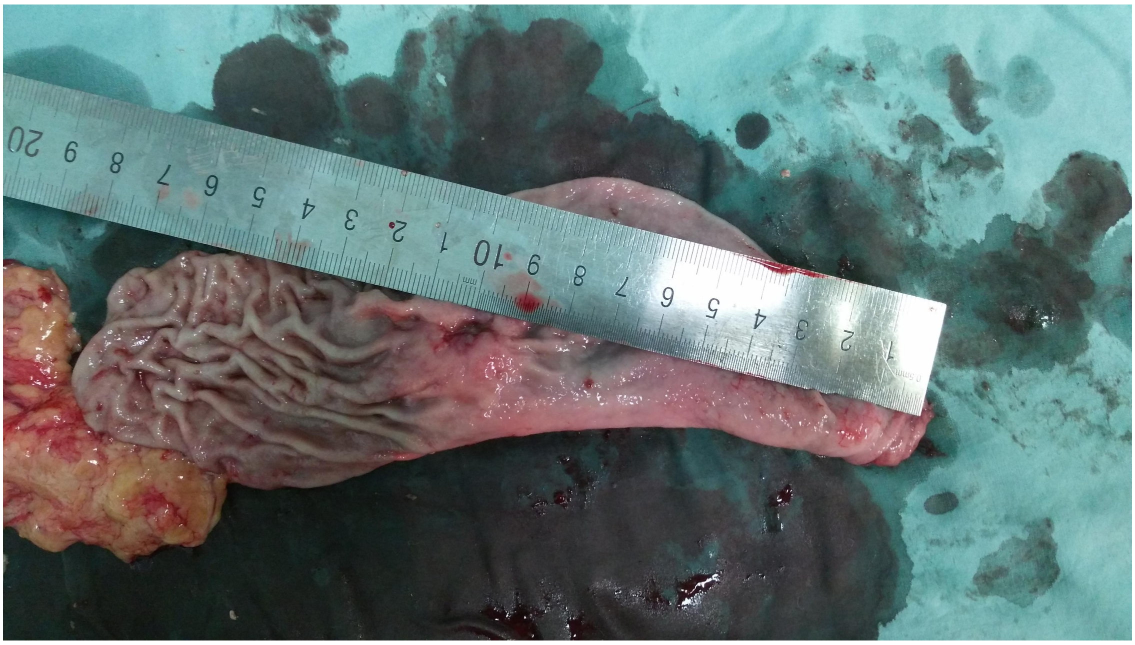

Fig. 2-9 Specimen observation (the distance between the tumor edge and the proximal incisional margin)

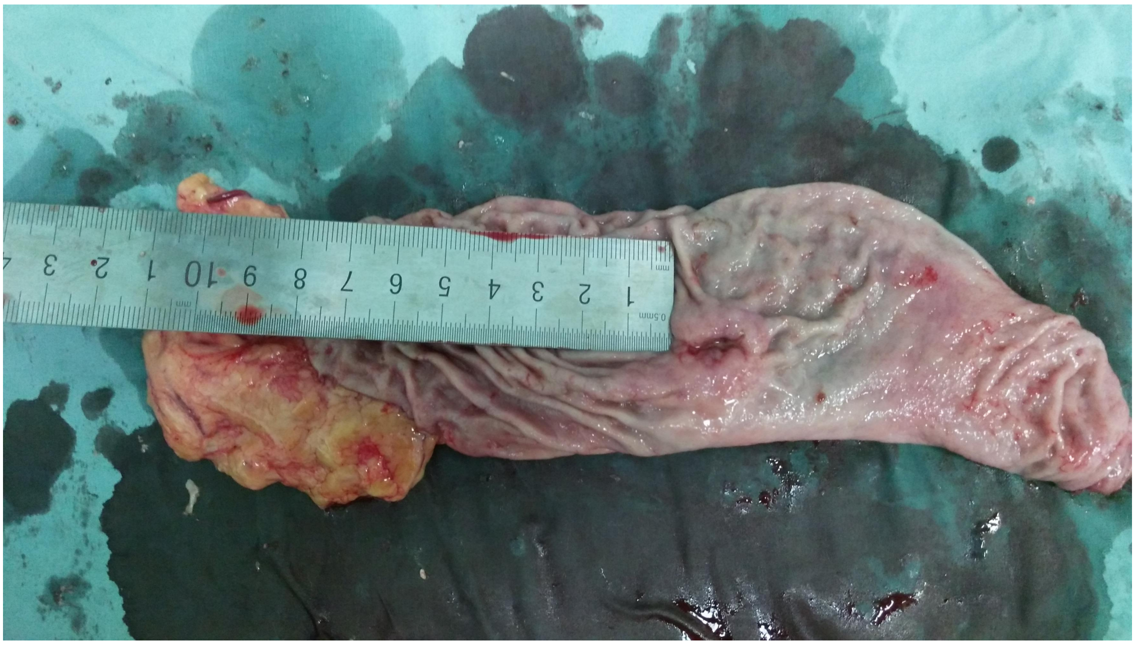

Fig. 2-10 Specimen observation (the distance between the tumor edge and the distal incisional margin)

9.3.1.13 Regulations on the photo/ image privacy protection and naming

No image data shall disclose the personal information of patients.

When the photos/images are viewed or reviewed, the personal information must be processed with mosaics or be covered.

The photographed parts should be marked with unified Chinese name: inferior pylorus area; left gastroepiploic vessel cut site; right-side area of superior margin of the pancreas; left-side area of superior margin of the pancreas; right side of the cardia and lesser gastric curvature side; incision appearance; specimen observation (before dissection); specimen observation (focus size); specimen observation (the distance between the tumor edge and the proximal incisional margin); and specimen observation (the distance between the tumor edge and the distal incisional margin).

For example:

Photo Name: [Robot-subject's random number - Inferior pylorus area]/ [Lap-ICG-subject's random number - Inferior pylorus area]

Folder name: [Robot-subject's random number]/ [LAP-ICG-subject's random number]

9.3.1.14 Criteria for confirming operation quality

To confirm the appropriateness of the surgical procedure, surgery quality, (auxiliary) incision length and specimen integrity will be assessed in the photographs saved (as stated above) The whole laparoscopic surgery procedure will be videotaped, and the unclipped image files will be saved.

9.3.1.15 Saving of imaging data

All photographs and data will be saved in the hard disk or portable digital carrier in digital form, and the surgical video required a specific hard drive to be saved for at least 3 years.

If failure to provide the complete photo according to “Regulations on imagery/photographing” is confirmed, the Research Committee will judge and record the surgery quality as unqualified; however, the case will remain in the PP set data of this study.

9.3.2Regulations on laparoscopic/robotic surgery

9.3.2.1 Regulations on pneumoperitoneum

Carbon dioxide pneumoperitoneum will be used to maintain the pressure at 12-13 mmHg.

9.3.2.2 Regulations on punctures and auxiliary incision

The positions of punctures and auxiliary small incision are not specified; the number of punctures should not exceed 5. There should be only one auxiliary small incision whose length shall not exceed the maximum tumor diameter and necessarily will be less than 10 cm in normal cases. If the auxiliary small incision needs to be longer than 10 cm, the surgeon in charge should make a decision and record the reasons in the CRF.

9.3.2.3 Definition of laparoscopic/robotic approach

The operations within the abdominal cavity must be performed using laparoscopic/robotic instruments with the support of a camera/Da Vinci system. Perigastric disassociation, greater omentum excision, omental bursa excision, lymph node dissection, and blood vessel handling are completed under laparoscopic/robotic guidance. For gastrectomy and digestive tract reconstruction use of auxiliary small incisions is allowed and can be completed with an opened abdomen.

9.3.2.4 Regulations on conversion to laparotomy

When intra-abdominal hemorrhage, organ damage and other serious/life-threatening complications which are difficult to control occur during laparoscopic surgery, it is necessary to actively convert to laparotomy. If the anesthesiologist and surgeon consider that intraoperative complications caused by carbon dioxide pneumoperitoneum may threaten the patient's life, it is necessary to actively convert to open. The surgeon in charge can decide to convert to laparotomy driven by other technical or equipment reasons and will record said reasons. The reasons for the conversion to open must be clearly recorded in the CRF. The incision length of > 10 cm is defined as a case of conversion to open surgery in this study.

9.3.2.5 Subsequent treatment of excluded patients from the laparoscopic group

Whether the patients continue to undergo surgery under laparoscopy or converted to open surgery is at surgeon's discretion according to clinical experience.

9.3.3 Operative parameters (same for both groups)

Completed by the research assistant on the day of the operation. specific projects include:

(1) Name of responsible surgeons

(2) Operation time (min)

(3) Type of operation, digestive tract reconstruction, intraoperative damage and whether the tumor was ruptured during surgery (intact rupture of the capsule)

(4) Length of incision (cm)

(5) Conversion to open surgery or not and the reasons for this decision

(6) Intraoperative estimated blood loss (ml; from skin cutting to stitching, intraoperative blood loss = (postoperative gauze weight, grams - preoperative gauze weight, grams) *1ml/g+ suction fluid, ml)

(7) Blood transfusion (ml): in this study, the blood transfusion event is defined as transfusion of red cell suspension (ml) or whole blood (ml)

(8) Tumor location

(9) Tumor size (maximum tumor diameter, mm)

(10) Distant metastasis (location)

(11) Proximal resected margin (mm), distal resected margin (mm), radicality（R0/R1/R2）

(12) Intraoperative complications (occurring from skin incision to skin closure)

including:

surgery-related complications: intraoperative hemorrhage and injury: A. Vascular injury: A vascular injury is defined as a blood vessel with either a blood vessel clamp or a titanium clamp closure and an intra-cavity suture or any other method to control the bleeding. B. Organ damage: maybe including diaphragmatic injury, esophageal injury, duodenal injury, colon injury, small intestine injury, spleen injury (excluding <1/3 spleen ischemia), liver injury, pancreatic injury, gallbladder injury, kidney damage etc.C. Tumor rupture: tumor envelope Integrity damage air abdominal-related complications: high-blood carbonate, mediastinal emphysema, subcutaneous emphysema, air embolism, respiratory circulation instability caused by abdominal pressure.

Anesthesia-related complications: Allergic reactions.

(13) Intraoperative death (occurring during the time period from skin cutting to skin stitching completion) regardless of reason.

9.4 Postoperative management (same for both groups**)**

9.4.1 The use of prophylactic analgesics

Continuous postoperative prophylactic intravenous analgesia is allowable but not mandatory within postoperative 48 hours; its dose, type and rate of infusion should be determined by the anesthesiologist according to clinical practices and specific patient conditions. The repeated use of prophylactic analgesics is not allowed beyond 48 hours after the end of surgery, unless it is judged necessary

9.4.2 Fluid replacement and nutritional support

Postoperative fluid infusion (including glucose, insulin, electrolytes, vitamins, etc.) or nutritional support (enteral/parenteral) will be performed based on doctor’s experience and routine clinical practices and is not specified in this study. After oral feeding, it is allowable to stop or gradually reduce fluid infusion/nutritional support.

9.4.3 Post-operative rehabilitation management

Management methods of incision, stomach and abdominal drainage tube: Follow regular diagnosis and treatment approaches. Eating recovery time, diet transition strategies: Follow regular diagnosis and treatment approaches.

9.4.4 Discharge standard

Patients needed to meet the following criteria for discharge: 1) satisfactory intake

of a soft diet. 2) move around of their bed. and 3) absence of complications by routine clinical examinations. This information will be recorded in the CRF.

9.4.5 Postoperative observation items

Definition of “postoperative day n”: One day from 0:00 to up to 24:00. Up to 24:00 on the day of surgery is “postoperative day 0;” the next day from 0:00 to up to 24:00 is “postoperative day 1;” and so on. From the first postoperative day until hospital discharge, the research assistant should timely fill in the following items and specific observation items including:

(1) Pathologic results：

Original lesion tissue typing, Distant metastasis, and parts, NIH Hazard grading, Radical surgery degree (R0/R1/R2)

(2) Postoperative complications：

Postoperative complications are divided into and short-term complications after surgery and long-term complications after surgery. Short-term is defined as within 30 days of surgery or the first discharge if the hospital days > 30 days. Long-term is defined as the period from 30 days or more after the operation, or the first discharge (the hospital days after surgery >30 days) to 3 years after the operation.

Classification and name of complication	Diagnostic criteria
Abdominal bleeding	Intra-abdominal hemorrhage requires blood transfusion, emergency endoscopy or surgical intervention to eliminate anastomotic bleeding
Anastomotic bleeding	The postoperative gastrointestinal decompression tube continued to have fresh red blood outflow; the hemoglobin drops more than 1g/dL
Gastrointestinal anastomotic stoma Fistula	Using gastrointestinal angiography to see contrast agent leak out from the anastomosis, or the blue drainage outflow through tube after oral Methylene blue to eliminate the possibility duodenal stump fistula and intestinal fistula
Duodenal Stump Fistula	Using gastrointestinal angiography to see contrast agent leak out from the duodenal stump to eliminate the anastomotic fistula or intestinal fistula
Intestinal fistula	Using gastrointestinal angiography to see the blue drainage outflow

	through tube after oral Methylene blue to eliminate anastomotic fistula and duodenal stump fistula
Stenosis of Anastomosis	Endoscopic examination with a 9.2-mm endoscopy not passing through the anastomosis to eliminate recurrence of tumors
Input jejunal loop obstruction	Abdominal pain, abdominal distension, vomiting and other symptoms. Abdominal flat to see the right upper abdomen expansion of the intestinal loop, and there is a liquid plane, or a visible input loop jejunum giant expansion by barium meal examination.
Intestinal obstruction after operation	Abdominal X-ray shows a plurality of liquid planes and the phenomenon of intestinal effusion with visible isolated, fixed, swelling of the intestinal loop. Total Abdominal CT showed edema, thickening, adhesion of intestinal wall, accumulation of gas in intestinal cavity, uniform expansion of bowel and intra-abdominal exudation.
Early dumping syndrome	Combined the symptoms of sweating, heat, weakness, dizziness, palpitations, heart swelling feeling, vomiting, abdominal colic or diarrhea with the signs of tachycardia, blood pressure micro-rise, breathing a little faster sign after meal 15-30 minutes, and solid phase radionuclide gastric emptying scanning tips stomach quickly emptying.
Late dumping syndrome	Feeling hungry, flustered, out of sweating 2-3 hours after the meal . Blood sugar is less than 2.9mmol/L, excluding other diseases that cause hypoglycemia
Intestinal ischemia and necrosis	Under the digestive endoscopy, the intestinal mucosa congestion, edema, bruising, mucosal hemorrhage, the mucous membrane being dark red, the vascular network disappearing, can have part mucosal necrosis, following with mucosal shedding, ulcer formation with annular, longitudinal, snake and scattered in the ulcer erosion.
Internal hernia	Postoperative CT findings of cystic or cystic and solid mass, and intestinal aggregation, stretching, translocation, abnormal mesenteric movement, and thickening of the blood vessel.
Alkaline reflux esophagitis	1. Endoscopic examination and biopsy of the upper gastrointestinal tract showed evidence of inflammation of the mucous membranes and gastrointestinal metaplasia; 2. CT scan and gastrointestinal barium meal examination showed no expansion or obstruction of the input loop.

Incision splitting	Including partial dehiscence of the incision and full-layer dehiscence
Incisional hernia of abdominal wall	The swelling tumor showing in the surgical scar area or abdominal wall swelling when standing or force. CT shows ventral wall continuity interruption and hernia content extravasation
Incision infection	Thickening of the soft tissue at the incision, in or below the incision of gas, exudation, swelling of the incision or pus from the incision extrusion, or secretion culture of pathogenic bacteria.
Lymphatic leakage	A chyle test when abdominal drainage fluid exceeded 300 ml/day for 5 consecutive days after postoperative day 3.
Pneumonia	Complies with one of the following two diagnostic Criteria: 1. Auscultation/percussion voiced + one of the following: fresh sputum or sputum character changes; blood culture (+); bronchoalveolar lavage fluid, anti-pollution sample brush, biopsy specimens cultured pathogenic bacteria. 2. Chest film hints of new or progressive infiltration + one of the following: fresh sputum or sputum character changes, blood culture (+), bronchoalveolar lavage fluid, anti-pollution sample brush, biopsy specimens cultured pathogenic bacteria; isolate virus or detect IgM, IgG (+) of respiratory viral
Acute pancreatitis	Irritability, abdominal pain, anti-jumping pain, fever, leukocyte increase and blood amylase increased occuring and diagnosed by ultrasound or CT within 3 days after surgery.
Acute cholecystitis	Serum bilirubin exceeding 85μmol/l and ultrsound examination shows gallbladder enlargement, wall thickness, signal and sound shadow of gallbladder stone, bile internal sediment, gallbladder contraction bad etc.
Pleural effusion/infection	CT scan showed the localized fluid low density area of thoracic cavity, which could accompany with gas, and culture pathogenic bacteria in thoracic endocrine.
Abdominal infection	There is at least one of the following evidences in abdominal cavity within 30 days after operation: 1. discharge of pus, with/without microbiological examination; 2. bacterial culture positive; 3. diagnosed by detection, pathology, imaging findings.
Pelvic infection	Symptoms of systemic infection or rectal irritation, combined with a rectal finger examination and touching tenderness, or a married

	woman with a posterior vault to extract pus-based fluid
Sepsis	The following two conditions are available: 1. There is evidence of active bacterial infection, but the blood culture does not necessarily appear pathogenic bacteria; 2. meeting two of the following four items at the same time: (1). body temperature >39. 0℃ or < 35.5 ℃ for 3 consecutive days, (2). heart rate > 120 times/min; (3). total white blood cells >12. 0*10^9/L or <4.0*10^9/l, wherein neutrophils >0. 80, or naïve granular cells >0. 10; (4).Respiratory frequency > 28 times/min
Urinary system infection	Symptoms of urine frequency, urgency and urine pain etc. and urine bacteria culture colony count 1000~10 million/ml in the absence of antibiotics; No symptoms of urine frequency, urgency and urine pain etc, urine bacterial culture colony count ≥ 100,000/ml
Pancreatic fistula	The level of amylase in the drainage fluid is three times than normal level.
Bile fistula	Symptoms of abdominal distension, Abdominal pain, tenderness, anti-jumping pain, muscle tension, abdominal puncture or drainage fluid for bile
Celiac fistula	The drainage fluid is milky white, and more than 200ml/d and and does not decrease for 48 hour, the celiac qualitative test is positive, and the level of triglyceride >110 mg/dL at the same time.
Nutritional disorder after gastrectomy	In the presence of weight loss, anemia, malnutrition bone disease, vitamin A deficiency and other symptoms, laboratory tests suggest that the intestinal absorption function test is abnormal, excluding other causes of nutritional disorders
Bone disease after gastrectomy	Lumbar back pain, length shortening, kyphosis, bone fractures and other symptoms. Bone density decreased combining with elevated alkaline phosphatase and serum calcium reduction, the concentration of serum 25-(O1) D3 and 1,25-(O1) 2D3 increasing and the serum parathyroid hormone increasing. Exclusion of bone disease caused by other causes.
Subcutaneous emphysema	visible the irregular speckle shadow under the skin in the horizontal flat sheet.
Mediastinal emphysema	In the posterior and anterior flat fame, a long narrow gas shadow rises

	to the neck soft tissue along the mediastinal side, forming a thin-line dense shadow. In the lateral flat there was a visible and clear band between the heart and the sternum. The CT examination, if necessary, shows gas density line-like shadow around the mediastinal and mediastinal pleura closing to the direction of the lung field.
Postoperative hemorrhage	An amount of hemorrhage exceeding 300 ml.
Postoperative cardiac dysfunction	The symptom of snus tachycardia, sinus bradycardia, supraventricular tachycardia, ventricular tachycardia, and other arrhythmias, or heart failure preoperatively none-existing and postoperatively appearing,.and other causes of the above-mentioned manifestations are excluded.
Hepatic dysfunction	Bilirubin increasing and the levels of AST and ALT >5 times after operation and these symptoms no existing before sugery,
Kidney function failure	Postoperative continuing renal function insufficiency, blood creatinine rising 2mg/dl, or acute renal failure needing dialysis treatment.
Cerebral embolism	Acute onset, hemiplegia, aphasia and other focal neurological function deficits. Embolism site has low-density infarction, of which border is not clear and no obstructional performance within 24-48 hours after the onset.
Pulmonary embolism	Characteristics of dyspnea, chest pain, syncope, shortness of breath, right ventricular insufficiency and hypotension, pulmonary angiography revealed a filling defect.
Venous thrombosis of lower extremities	Local tenderness, swelling, purple skin color, combined with intravenous angiography to show the filling defect
Mesenteric arterial embolization	Patients with acute abdominal pain, vomiting, diarrhea, abdominal x-ray of intestinal tract filling with gas or existing liquid level, abdominal angiography revealed a filling defect.
DIC	1.There are basic diseases easily leading to DIC, 2. There are more than two clinical performances: (1) severe or multiple bleeding tendencies; (2) Microcirculation disorder or shock cannot be explained by the original disease. (3) Extensive skin mucosal embolism, focal ischemic necrosis, shedding and ulcer formation, or unexplained lung, kidney, brain and another organ failure. (4) anticoagulant treatment.is effective. 3. The laboratory meets the following

	conditions: (1) there are 3 or more experimental abnormalities: platelet count, prothrombin time, activated partial coagulation enzyme time, thrombin time, fibrinogen level, D-two poly, and (2) difficult or special cases for special examination.
Other	Complications other than the above complications, which do not exist before surgery but appear after surgery

Severity of complication is graded according to Clavien–dindo complication scoring system, [31]
IIIA level and above are serious complication

Ⅰ: Any deviation from the normal postoperative course without the need for pharmacologic treatment or surgical, endoscopic, and radiologic interventions. Allowed therapeutic regimens are drugs as antiemetics, antipyretics, analgesics, and diuretics, and electrolytes and physiotherapy. This grade also includes wound infections opened at the bedside.

Ⅱ： Requiring pharmacologic treatment with drugs other than such allowed for

grade I complications. Blood transfusions and total parenteral nutrition are also included.

Ⅲ：Requiring surgical, endoscopic, or radiologic intervention

Ⅲa：Intervention not under general anesthesia

Ⅲb：Intervention under general anesthesia

Ⅳ： Life-threatening complication (including CNS complications) requiring IC(intermediate care)/ICU(intensive care unit)
management

Ⅳa：Single organ dysfunction (including dialysis)

Ⅳb：Multiple organ dysfunction

Ⅴ： Death as a result of complications

(3) Blood test items (At postoperative day 1, 5)

Peripheral blood routine assessment: Hb, RBC, WBC, LYM, NEU, NEU%, and PLT、MONO；

Blood biochemistry: Albumin, prealbumin, total bilirubin, AST, ALT, creatinine, urea nitrogen, fasting blood glucose, potassium, sodium, chlorine, calcium and CRP.

(4) Postoperative rehabilitation evaluation：

Time to first ambulation (hours), time to first flatus (hour), time to liquid diet, time to semi-liquid diet (hour), daily body temperature maximum from surgery to out-patient (℃), time to removal of gastric tube (d), daily volume of gastric drainage (ml), time to removal of abdominal drainage tube (d), daily volume of drainage (ml).

Blood transfusion volume (ml) from the end of surgery to postoperative discharge: a transfusion event is defined as infusion of the red blood cell suspension (ml) or whole blood (ml)

Postoperative hospital stay (days): periods form surgery day to first discharge day

9.5 Follow-Up

9.5.1 Follow-up Period and strategy

Follow-up visits will be completed by special persons for all cases selected in this study .All patients are followed up with every 3 months during the first 2 years and then every 6 months beyond the third year (1、 3、 6、 9、 12、 15、18、21、24、30 and 36 months after the operation). This study suggests that the above examinations should be conducted in the patient's primary surgical research center, but does not exclude outer court review. For Outer Court review, It recommended that visiting the hospital as a three-level hospital, and these information will be recorded by the follow-up specialist. The occurrence of tumor recurrence or metastasis and the survival status of all patients are evaluated and recorded according to the results of the various examinations. Patients who refuse to follow the protocol should be recorded as lost to follow-up, and at the end of the study, these cases should be analyzed together with cases lost to follow-up in line with the criteria of this study.

9.5.2 Assessment items during the follow-up

(1) Systematic physical examination:

The doctor in charge will regularly conduct a systematic physical examination at the time of each follow-up, giving particular attention to superficial lymph nodes, abdomen, and signs of metastases, among others.

(2) Blood test items:

Peripheral blood routine assessment: Hb、RBC、WBC、LYM、NEU、NEU%、PLT、MONO

Biochemistry: Albumin, pre-albumin, total bilirubin, Indirect bilirubin, direct

bilirubin, AST, ALT, creatinine, urea nitrogen, Total cholesterol, triglycerides, fasting blood glucose, potassium, sodium, chlorine, calcium, serum tumor markers: CEA、CA19-9、CA72-4、CA12-5、AFP

(3) Imaging items:

Whole abdomen (including cavity) CT (thickness of 10 mm or less, in case of contrast agent allergy, CT horizontal scanning is only allowable or conversion to MRI). Upper gastrointestinal endoscopy (histopathological biopsy, endoscopic ultrasonography when necessary). Chest X-ray (AP and lateral views): lung field condition. Other means of evaluation: gastrointestinal radiography, ultrasonography of other organs, whole body bone scanning, and PET-CT, among others used at physician's discretion.

9.5.3 Follow-up process

Postoperative	3 months	6 months	9 months	12 months	15 months	18 months	21 months	2 years	2 years and a half	3 years
Date of actual visit										
Physical examination										
Blood Routine										
Blood biochemistry										
Tumor Markers										
Chest slices										
Upper digestive tract endoscopy										
Abdominal CT										
Full abdominal ultrasound										

Other (if necessary)										

9.6 Post-operative adjuvant therapy

9.6.1 Indications for postoperative adjuvant chemotherapy

After completion of the surgical treatment, according to the postoperative pathological results, subjects among the R0 resection cases that are stage II and above are administered postoperative adjuvant chemotherapy according to the provisions of this program.

For cases of non-R0 resection or recurrence after R0 resection, this study does not stipulate the follow-up treatment plan; each research center decides on the action to be taken according to the clinical treatment routine.

9.6.2 Postoperative adjuvant chemotherapy

This study uses a combination of chemotherapy based on 5-FU (5-fluorouracil) with platinum or docetaxel.

The adjuvant chemotherapy cycle is half a year (6 months postoperatively).

In cases of good physical and tolerable conditions, chemotherapy is first started within 8 weeks after surgery and then according to the regularity of the chemotherapy cycle.

During the chemotherapy period, tumor recurrence should be assessed according to the follow-up plan.

When tumor recurrence occurs during chemotherapy, the adjuvant chemotherapy regimen of this study is discontinued. The follow-up treatment is decided by each research center according to the clinical treatment routine. This study does not make regulations, but the cause and follow-up treatment plan should be recorded in the CRF.

If there is no recurrence during chemotherapy, adjuvant chemotherapy is terminated after 6 months, and the follow-up plan continues.

Adjuvant chemotherapy requires written approval from the patient.

Subjects that refuse postoperative adjuvant chemotherapy or do not complete

the adjuvant chemotherapy are not excluded from this study, but the cause is marked and recorded in the CRF.

For elderly patients (70 years and older), considering differences in the physical fitness of the elderly and ensuring the safety of patients, each research center decides according to the clinical treatment routine. This study does not recommend or stipulate any chemotherapy regimen for patients of this age.

Patients who choose adjuvant chemotherapy, irregular chemotherapy, or a nonfirst-line regimen are not excluded from the study, but the Efficacy and Safety Evaluation Committee is obliged to monitor patient safety during follow-up. The patient's chemotherapy medication must be recorded in the CRF.

The principles of processing in terms of the method of administration of adjuvant chemotherapy, toxic reactions, and dose adjustment with intolerance are implemented according to the original literature on drug toxicity and dose adjustment for each chemotherapy regimen. This study does not regulate these principles.

9.6.3 Safety Evaluation Indicators of Postoperative Adjuvant Chemotherapy

The safety evaluation indicators for patients enrolled in the study should be immediately filled out by the investigators before and after each postoperative adjuvant chemotherapy cycle, with specific items including the following:

(1) Performance Status (ECOG)

(2) Subjective and objective status (according to the records of CTCAE v3.0 Short Name)

(3) Blood tests:

Peripheral venous blood assessment: Hb, RBC, WBC, LYM, NEU, NEU%, PLT, MONO.

Blood biochemistry: albumin, prealbumin, total bilirubin, AST, ALT, creatinine, urea nitrogen, fasting blood glucose, serum tumor markers (CEA, CA19-9, CA72-4, CA12-5, AFP)

(4) Safety evaluation items to be implemented during chemotherapy when necessary (refer to CTCAE v3.0):

Neurotoxicity

Cardiovascular system (cardiac toxicity, ischemic heart disease, etc.)

Bone marrow suppression and infections due to immune dysfunction

Others

9.7 Study calendar

Observation Stage		Performance Status	Blood biochemistry	Tumor markers	Electrocardiogram, respiratory function	Upper gastrointestinal endoscopy	Chest X-ray, full abdominal CT Or ultrasound	Eligibility confirmation notice	Preoperative, postoperative complications	Adverse chemotherapy events	CRF- Preoperative	CRF-Intraoperative	CRF- Postoperative	CRF- treatment end	CRF- follow-up observation surgery
Selection Application		○	○	○	○	○	○								
After selection and prior to surgery								○			○				
Intraoperative period									○			○			
Early postoperative period									○				○	○	
Before postoperative first chemotherapy		○	○	○			○								
Regular chemotherapy		○	○	○						○					
Follow-up	At postoperative 1 month	○	○	○			○		○						○

	(±7 days)														
	At postoperative 3 months (±15 days)	○	○	○					○						○
	At postoperative 6 months (±15 days)	○	○	○			○		○						○
	At postoperative 9 months (±15 days)	○	○	○					○						○
	At postoperative 1 year (±15 days)	○	○	○			○		○						○
	At postoperative 15 months (±15 days)	○	○	○					○						○
	At postoperative 18 months (±15 days)	○	○	○			○		○						○
	At postoperative 21 months (±15 days)	○	○	○					○						○

	At postoperative 2 years (±15 days)	○	○	○				○	○						○
	At postoperative 2 years (±15 days)	○	○	○				○	○						○
	At postoperative 3 years (±15 days)	○	○	○				○	○						○

○: must do

9.8 Definitions involved in SOP

9.8.1 ECOG performance status score

According to the simplified performance status score scale developed by the ECOG, the patients' performance status can be classified into 6 levels, namely 0-5, as follows:

0: Fully active, able to carry on all pre-disease performance without restriction

1: Restricted in physically strenuous activity but ambulatory and able to carry out work of a light or sedentary nature, e.g., light housework, office work

2: Ambulatory and capable of all self-care but unable to carry out any work activities. Up and about more than 50% of waking hours

3: Capable of only limited self-care, confined to bed or chair more than 50% of waking hours

4: Completely disabled. Cannot carry on any self-care. In total, confined to bed or chair

5: Dead

Patients at levels 3, 4 and 5 are generally considered to be unsuitable for surgical treatment or chemotherapy.

9.8.2 ASA classification

According to the patients' physical status and surgical risk before anesthesia, the American Society of Anesthesiologists (ASA) has categorized patients into 5 levels (I-V levels):

Class I: Well-developed patients with physical health and normal function of various organs, with a perioperative mortality rate of 0.06% -0.08%.

Class II: Patients with mild complications and good functional compensation in addition to surgical diseases, with a perioperative mortality rate of 0.27% -0.40%.

Class III: Patients with severe complications and restricted physical activity but still capable of coping with day-to-day activities, with a perioperative mortality rate of 1.82% -4.30%.

Class IV: Patients with serious complications who have lost the ability to perform day-to-day activities, often have life-threatening conditions, and a perioperative mortality rate of 7.80% -23.0%.

Class V: Moribund patients either receiving surgery or not, have little chance for survival, and a perioperative mortality rate of 9.40% -50.70%.

Generally, Class I/II patients are considered good for anesthesia and surgical tolerance, with a smooth anesthesia process. Class III patients are exposed to some anesthesia risks; therefore, good preparations should be fully made before anesthesia, and effective measures should be taken to prevent potential complications during anesthesia. Class IV patients are exposed to the most risks, even if good preoperative preparations are made, and have a very high perioperative mortality rate. Class V patients are moribund patients and should not undergo an elective surgery.

9.8.3 Oncology-related definitions

In this study, tumor staging is based on AJCC-8th; surgical treatment follows the Japanese Gastric Cancer Treatment Guidelines, Physicians Edition, 4rd Edition, 2014, and other writing and recording principles follow the Japanese Gastric Cancer Statute 15th.

9.8.3.1 Tumor staging record

9.8.3.1.1 Recording principle

The two staging records for clinical classification and pathological classification involve T (invasion depth), N (regional lymph node) and M (distant metastasis), which are expressed in Arabic numerals and denoted as x if indefinite.

Clinical classification	Pathological classification

Physical examination X-ray, endoscopy, diagnostic imaging<br>laparoscopy, intraoperative observations (laparotomy/laparoscopy), biopsy, cytology, biochemistry, biology examination	Pathological diagnosis of the endoscopic/surgical specimens<br>Intraperitoneal exfoliative cytology

9.8.3.1.2 Records of tumor invasion depth

Tumor invasion depth is defined as follows:

TX: Unknown cancer invasion depth

T0: No cancer found

T1: Cancer invasion is only confined to the mucosa (M) or the submucosal tissue (SM)

- T1a: Cancer invasion is only confined to the mucosa (M)
- T1b: Cancer invasion is confined to the submucosal tissue (SM)

T2: Cancer invasion exceeds the submucosal tissue but is only confined to the inherent muscular layer (MP)

T3: Cancer invasion exceeds the inherent muscular layer (MP) but is only confined to the subserosal tissue (SS)

T4: Cancer invasion involves the serosa (SE) or direct invasion of adjacent structures (SI)

- T4a: Cancer invasion involves only the serosa (SE)
- T4b: Cancer directly invades the adjacent structures (SI)

9.8.3.1.3 Records of tumor metastasis

(1) Lymph node metastasis:

NX: Number of lymph node metastases is unknown

N0: No lymph node metastasis

N1: Lymph node metastasis of 1-2 areas

N2: Lymph node metastasis of 3-6 areas

N3: Lymph node metastasis of 7 and more areas

- N3a: Lymph node metastasis of 7-15 areas
- N3b: Lymph node metastasis of 16 and more areas

Lymph node numbers are defined as follows:

No.	Name	Definition
1	Cardia right	Lymph nodes around the gastric wall first branch (cardia branch) of ascending branches of the left gastric artery and those at the cardia sides
2	Cardia left	Lymph nodes at the left side of the cardia and those along the cardia branch of the lower left diaphragmatic artery esophagus
3a	Lesser gastric curvature (along the left gastric artery)	Lymph nodes at the lesser curvature side along the left gastric artery branch, below the cardia branch
3b	Lesser gastric curvature (along the right gastric artery)	Lymph nodes at the lesser curvature side along the right gastric artery branch, partial left side of the 1st branch in the lesser curvature direction
4sa	Left side of the greater gastric curvature (short gastric artery)	Lymph nodes along the short gastric artery (excluding the root)
4sb	Left side of the greater gastric curvature (along the left gastroepiploic artery)	Lymph nodes along the left gastroepiploic artery and the first branch of the greater curvature (refer to the definition of No. 10)
4d	Right side of the greater gastric curvature	Lymph nodes at the partial left side of the first branch in the greater gastric curvature direction along the right gastroepiploic artery

	(along the right gastroepiploic artery)	
5	Superior pylorus	Lymph nodes along the right gastric artery and around the first branch in the lesser gastric curvature direction
6	Inferior pylorus	Lymph nodes from the root of the right gastroepiploic artery to the first branch in the greater gastric curvature direction and those at the junction of the right gastroepiploic veins and superior anterior pancreaticoduodenal veins (including the junction portion)
7	Left gastric artery trunk	Lymph nodes from the root of the left gastric artery to the branch portion of the ascending branches
8a	Anterior upper part of the common hepatic artery	Lymph nodes at the anterior upper part of the common hepatic artery (from the branch portion of the splenic artery to the branch portion of the gastroduodenal artery)
8p	Posterior part of the common hepatic artery	Lymph nodes at the posterior part of the common hepatic artery (from the branch portion of the splenic artery to the branch portion of the gastroduodenal artery)
9	Surrounding of the celiac artery	Lymph gland that is in the surroundings of the celiac artery or that is a part of each root of the left artery of the stomach, common hepatic artery and splenic artery as well as that related to the celiac artery
10	Splenic hilum	Lymph gland that is in the surroundings of the celiac artery and splenic hilum far away from the end of the pancreas, including the first greater gastric curvature in the root of the short gastric artery and the left gastroepiploic artery
11p	Splenic artery proximal	Lymph gland at the splenic artery proximal (in a location that divides the distance between the root of the splenic artery and the end of the pancreas into two equal parts, including the proximal

		side)
11d	Splenic artery distal	Lymph gland at the splenic artery distal (in a location that divides the distance between the root of the splenic artery and the end of the pancreas into two equal parts, inclining to the end of the pancreas)
12a	Within the hepatoduodenal ligament (along the proper hepatic artery)	Lymph gland that is below a location that divides the height of the confluence portions of the left and right hepatic ducts and the bile duct in the upper margin of the pancreas into two equal parts and is along the proper hepatic artery (as stated in No. 12a2 of the regulations for bile duct carcinoma)
12b	Within the hepatoduodenal ligament (along the bile duct)	Lymph gland that is below a location that divides the height of the confluence portions of the left and right hepatic ducts and the bile duct in the upper margin of the pancreas into two equal parts and is along the proper hepatic artery (as stated in No. 12b2 of the regulations for bile duct carcinoma)
12p	Within the hepatoduodenal ligament (along the portal vein)	Lymph gland that is below a location that divides the height of the confluence portions of the left and right hepatic ducts and the bile duct in the upper margin of the pancreas into two equal parts and is along the proper hepatic artery (as stated in No. 12p2 of the regulations for bile duct carcinoma)
13	Back of the pancreatic head	Lymph gland adjacent to the head of the duodenal papilla at the back of the pancreatic head (No. 12b in the surroundings of the hepatoduodenal ligament)
14v	Along the superior mesenteric vein	Lymph gland that is in the front of the superior mesenteric vein, with the inferior margin of the pancreas on the upper side, the right gastroepiploic vein and confluence portion of the superior pancreaticoduodenal vein to the right, the left margin of the

		mesenteric vein to the left and the branch of the middle colic vein in the lower margin
14a	Along the superior mesenteric artery	Lymph gland along the superior mesenteric artery
15	Surroundings of the colon middle artery	Lymph gland that is in the surroundings of the colon middle artery
16a1	Surroundings of the abdominal aorta a1	Lymph gland that is in the surroundings of the aorta gap (4 to 5 cm wide in the surroundings of the medial crus of the diaphragm)
16a2	Surroundings of the abdominal aorta a2	Lymph gland that is in the surroundings of the aorta from the upper margin of the abdominal artery root to the lower margin of the left renal vein
16b1	Surroundings of the abdominal aorta b1	Lymph gland that is in the surroundings of the aorta from the lower margin of the left renal vein to the upper margin of the inferior mesenteric artery root
16b2	Surroundings of the abdominal aorta b2	Lymph gland that is in the surroundings of the aorta from the upper margin of the inferior mesenteric artery root to the branch of aorta
17	Front of the pancreatic head	Lymph gland that is in the front of the pancreatic head, next to the pancreas and under the pancreatic capsule
18	Below the pancreas	Lymph gland that is in the lower margin of the pancreas
19	Below the diaphragm	Lymph gland that is in the cavity of the diaphragm and along the lower side of the diaphragmatic artery

20	Hiatal part of the gullet	Lymph gland that connects the hiatal part of diaphragm to the gullet
110	Beside the lower gullet	Lymph gland that departs from the diaphragm and is next to the lower gullet
111	Above the diaphragm	Lymph gland that is in the cavity of the diaphragm and departs from the gullet (No. 20 that connects to the diaphragm and gullet)
112	Posterior mediastinum	Lymph gland of the posterior mediastinum departed from the gullet and its hiatal portion

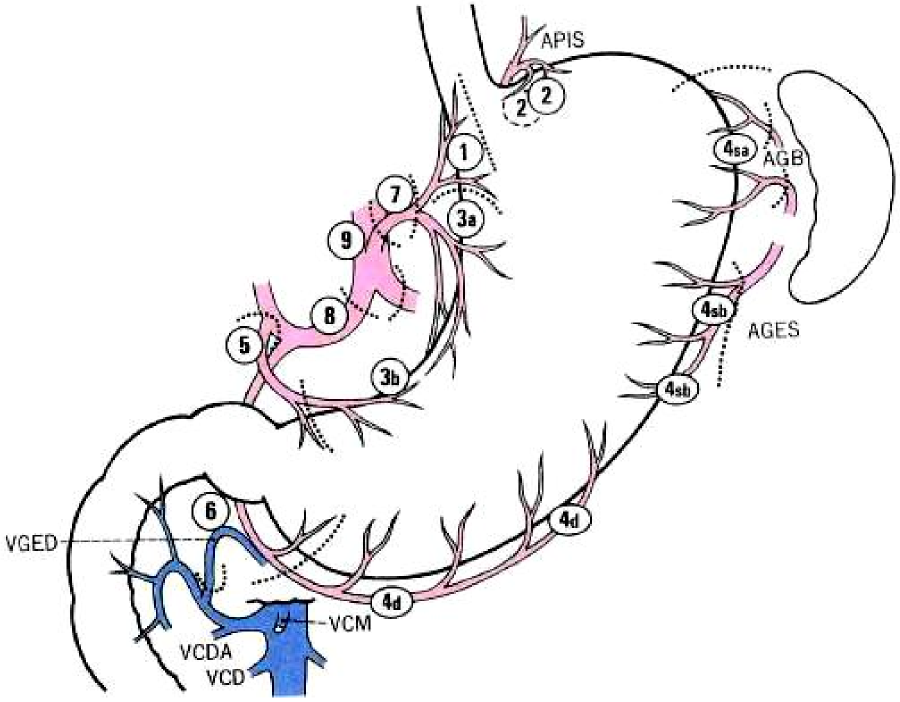

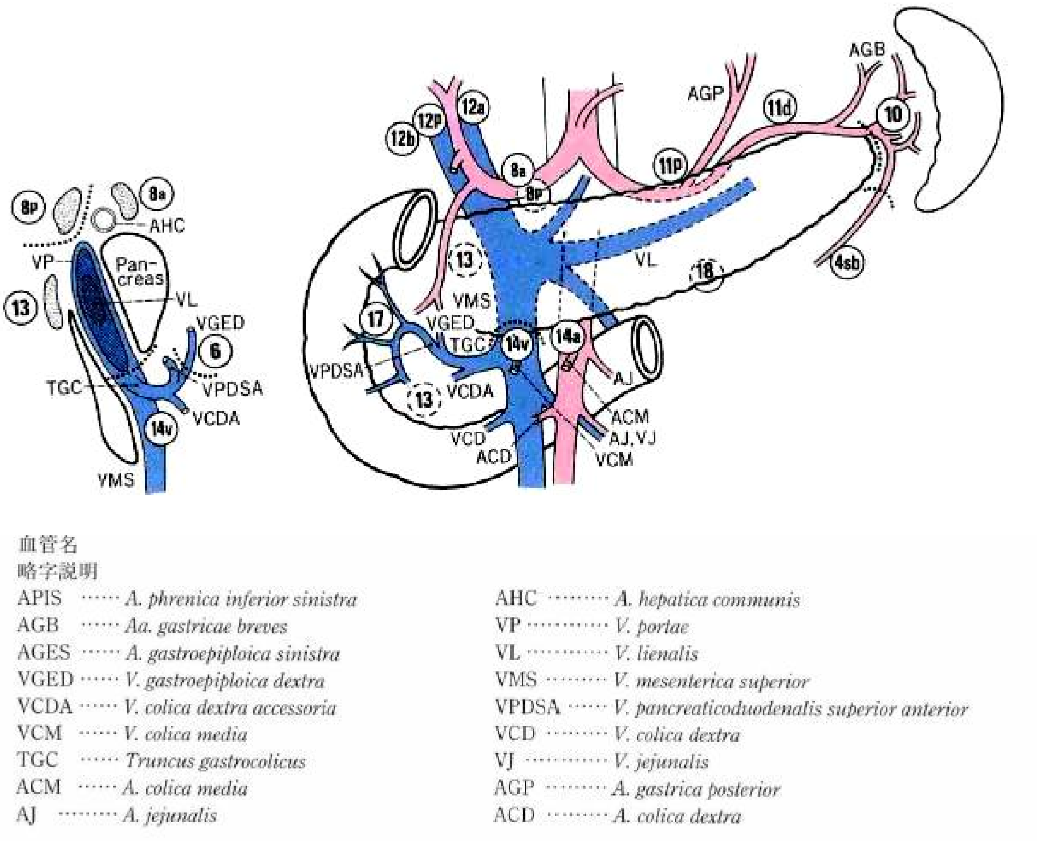

Fig. 4. Lymph node grouping

(2) Distant metastasis

M0: No distant metastasis outside of the regional lymph nodes

M1: Distant metastasis outside of the regional lymph nodes

MX: Presence of distant metastasis is unclear

Record the specific sites under the M1 condition: peritoneum (PER), liver (HEP), lymph node (LYM), skin (SKI), lung (PUL), bone marrow (MAR), bone (OSS), pleura (PLE), brain (BRA) and meninges (MEN), intraperitoneal exfoliated cells (CY), and others (OTH). Note: A positive examination result for intraperitoneal exfoliated cells is recorded as M1.

9.8.3.1.4 Tumor Staging

Pathological (pTNM)					
T/M	N0	N1	N2	N3a	N3b
T1	IA	IB	IIA	IIB	IIIB
T2	IB	IIA	IIB	IIIA	IIIB
T3	IIA	IIB	IIIA	IIIB	IIIC
T4a	IIB	IIIA	IIIA	IIIB	IIIC
T4b	IIIA	IIIB	IIIB	IIIC	IIIC
M1	IV	IV	IV	IV	IV

9.8.3.2 Pathologic types and classifications

9.8.3.2.1 Type

Papillary adenocarcinoma

Tubular adenocarcinoma

Mucinous adenocarcinoma

Signet ring cell carcinoma

Poorly differentiated carcinoma

9.8.3.2.2 Grading

GX classification is not possible to assess

G1 well-differentiated

G2 moderately differentiated

G3 poorly differentiated

G4 undifferentiated

9.8.3.3 Evaluation of Radical Level (Degree)

9.8.3.3.1 Recording the Presence or Absence of Cancer Invasion on the Resection Stump

(1) Proximal incisional margin (PM: proximal margin)

PM (-): No cancer invasion found on the proximal incisional margin

PM (+): Cancer invasion found on the proximal incisional margin

PM X: Unknown cancer invasion on the proximal incisional margin

(2) Distal incisional margin (DM: distal margin)

DM (-): No cancer invasion found on the distal incisional margin

DM (+): Cancer invasion found on the distal incisional margin

DM X: Unknown cancer invasion on the distal incisional margin

9.8.3.3.2 Radical Records

Postoperative residual tumor, denoted with R (residual tumor): R0: curative resection; R1, R2: non-curative resection.

RX: cannot be evaluated

R0: no residual cancer

R1: microscopic residual cancer (positive margins, peritoneal lavage cytology positive)

R2: macroscopic residual cancer

10 Statistical analysis

10.1 Definition of the population

(1) ITTP, intent-to-treat population

(2) MITTP, modified intent-to-treat population

(3) PPP, per-protocol population

(4) SAP, safety analysis population

10.2 Statistical analysis plan

- Statistical software: We will use Epidata3.0 to establish a database and to input data， and we will use SPSS18.0 software to perform statistical analyses.
- Basic principle：The method of differential testing was adopted. The safety population of the study consists of the patients who receive safety evaluation data after the intervention. Descriptive statistics and two-sided tests were conducted for the safety indicators and the incidence of adverse reactions. A p-value <0.05 is considered statistically significant. The confidence interval of the parameters is estimated with a 95% confidence interval.
- Shedding analysis: Total shedding rate of two groups and loss rate due to adverse events will be compared using pearsonχ^2 test
- Statistical analysis of population division: baseline data and effective analysis using MITT analysis. The main therapeutic indicators are analyzed using both MITT and PP analysis. But based on the conclusion of MITT analysis. If MITT analysis and PP analysis of the conclusions are consistent, it can increase the credibility of the conclusion. The data of laboratory examination, adverse events and adverse reactions were analyzed by SAP. The incidence rate of adverse reactions uses SAP as the denominator.

- Method of outlier determination: the observation value is greater than P75 or less than P25, and the exceed value more than 3 times of the quartile spacing (=p75-p25), which will be sentenced to outlier data. During the analysis, the sensitivity analysis is used for outlier data, namely analyzing outcomes including or excluding, outliers data. and if the results are not contradictory, the data is retained; if the contradiction, it depends on the specific circumstances.
- Descriptive statistics: The measurement data gives the mean, the standard deviation and the confidence interval, and the minimum value, the maximum value, the P25, the median and the P75 are given when necessary; matched data also gives the mean and standard deviation of the gap-value, and the median and average rank of the Non-parametric method. The nominal-scale data gives the frequency distribution and the corresponding percentages. The level data gives the frequency distribution and the corresponding percentages, as well as the median and the average rank. Qualitative data give positive rate, positive number, and denominator numbers. The survival data gives the number of events, the number of deletions, the median survival time, and the survival rate.
- Subgroup analysis： Sub-group analysis is to find the factors that may affect prognostic according to the specific circumstances of the data.
- Missing values handling： This study does not fill in missing values
- Effective analysis: Using Log-rank test for single factor analysis of Survival Time Data, using Cox regression model Analysis for multi-factor analysis. Quantitative data using t test or t' Test (variance is not homogeneous), qualitative data using Pearson χ^2 test, grade data using Wilcoxon rank test.
- Safety analysis: counting adverse responds incidence and incidence of adverse events and make a list to describe the adverse events occurring in the study. describe the results of the laboratory tests before and after the normal/abnormal changes and the relationship between the abnormal changes and drugs in the research, and make a list on the "normal/abnormal" changes occurred in the study.. More detailed statistical analysis is shown in the statistical analysis plan.

11 Data management

11.1 Case Report Form (CRF)

11.1.1 CRF Types and Submission Deadline

CRFs used in this study and their submission deadlines are as follows:

(1) Case Screening: 7 days prior to surgery (time frame of 3 days)

(2) Enrolling: submitted to the data center at one day prior to surgery

(3) Surgery: within 1 day after surgery

(4) Postoperative discharge: within 3 days after the first discharge

(5) Follow-up records: 7 days after each specified follow-up time point

11.1.2 Method of transmission of CRF

In this study, the paper CRF form are used for information and data transmittal.

11.1.3 Revision of CRF

After the start of the study, if the CRF is found to lack items that are then deemed pertinent, under the premises of ensuring the amendment of the CRF does not cause medical and economic burden and increased risks to the selected patients, the CRF can be modified after the Research Committee adopt it through discuss at the meeting. If the amendment of the CRF requires no changes to this study protocol, the latter will not be modified.

11.2 Monitoring and Supervising

To assess whether study implementation follows protocol and data are being collected properly, monitoring should be conducted every February during the follow-up period. Monitoring is to complete through visiting a hospital and comparing the original Data.

11.2.1 Monitoring item

- Data Collection Completion Status: By selected registration numbers (cumulative and for each time period)
- Eligibility: Not eligible patients/potentially ineligible patients
- Different end of treatment, the reasons for suspension/end of the study protocol
- Background factors, pre-treatment report factors, post-treatment report factors when selected for registration
- Severe adverse events
- Adverse events/adverse reactions
- Laparoscopic surgery completion percentage

- Proportion of conversion to laparotomy
- Protocol deviation
- Disease-free survival /overall survival (all enrolled Patients)
- Progress and safety of the study, other issues

11.2.2 Acceptable range of adverse events

Treatment-related death and life-threatening complications caused by surgeries occur relatively rarely and partly are dependent on the qualifications of the research participating hospitals and their staff; a rate of over 3% is considered unacceptable. If treatment-related death is suspected or non-hematologic Grade 4 toxicity having a causal relationship with the surgery is determined, adverse events should be reported to the Efficacy and Safety Evaluation Committee. If the number of treatment-related deaths or the number of patients with determined non-hematologic Grade 4 toxicity having a causal relationship with the surgery reached 15, the final incidence proportion of adverse events would be expected to exceed 3%, and therefore the inclusion of patients must be immediately suspended. Whether the study can continue should be determined by the Efficacy and Safety Evaluation Committee.

12 Relevant Provisions on adverse events

12.1 Surgery-related adverse events

See the adverse events mentioned for surgical complications in 8.1 Definition of the study endpoint.

12.2 Various forms of adverse events caused by original incidence

Adverse events relating to various forms of deterioration in primary diseases should be recorded according to Short Name of CTCAEv3.0.

12.3 Evaluation of adverse events

- Evaluation of adverse event/adverse reaction are based on[Accordion Severity Grading System] and [CTCAE v3.0].
- Adverse events will be graded 0 ~ 4 as per definition. For treatment-related death, fatal adverse events are classified as Grade 5 in the original CTCAE
- Toxicity items specified in the [surgery-related adverse events], Grade and the discovery date of Grade should be recorded in the treatment process report. For other toxicity items observed, observed Grade 3 toxicity items are only recorded in the

freedom registration column of the treatment process report, as well as Grade and the discovery date of Grade. Grade recorded in the treatment process report must be recorded in the case report form.

- CTCAE v3.0, the so-called “Adverse Event”, “all observed, unexpected bad signs, symptoms and diseases (abnormal value of clinical examination are also included) in the treatment or disposal, regardless of a causal relationship with the treatment or handling, including determining whether there is a causal relationship or not”.
- Therefore, even if events were “obviously caused by primary disease (cancer)” or caused by supportive therapy or combination therapy rather than the study regimen treatment (protocol treatment), they are “adverse events”.
- For adverse event data collection strategy, the following principles should be complied with in this study:1) Adverse events within 30 days from the last treatment day of the study regimen treatment (protocol treatment), regardless of the presence or absence of a causal relationship should be completely collected. (When adverse events are reported, the causality and classification of adverse events are separately discussed) 2) Adverse events within 30 days from the last treatment day of the study regimen treatment (protocol treatment), regardless of the presence or absence of a causal relationship should be completely collected. (When adverse events are reported, the causality and classification of adverse events are separately discussed)

12.4 Reporting of Adverse Events

- When “severe adverse events” or “unexpected adverse events” occur, the Research Responsible Person of research participating unit should report them to the Research Committee (Chang-Ming Huang).
- Based on the relevant laws and regulations, adverse events should be reported to the province (city) Health Department at the location of each research center. Severe adverse events based on clinical research-related ethical guideline should be reported to the person in overall charge of the medical institution. The appropriate reporting procedures should be completed in accordance with the relevant provisions of all medical institutions at the same time. The person in charge of research of each research participating unit should hold accountability and responsibility for the emergency treatment of patients with any degree of adverse

events to ensure patient safety.

12.4.1 Adverse Events with Reporting Obligations

12.4.1.1 Adverse Events with Emergency Reporting Obligations

Any of the following adverse events should be reported on an emergent basis:

- All patients who die during the course of treatment or within 30 days from the last treatment day, regardless of the presence or absence of a causal relationship with the study regimen treatment. Also, cases of discontinuation of treatment, even if within 30 days from the last treatment day, those patients are also emergent reporting objects. ("30 days" refers to day 0, the final treatment day, 30 days starting from the next day)
- Those patients with unexpected Grade 4 non-hematologic toxicity (CTCAE v3.0 adverse events other than the blood/bone marrow group), having a causality of treatment (any of definite, probable, possible) who emergent reporting objects are.

12.4.1.2 Adverse Events with Regular Reporting Obligations

One of the following adverse events are regular reporting objects:

(1) After 31 days from the last treatment day, deaths for which a causal relationship with treatment cannot be denied, including suspected treatment-related death; death due

to obvious primary disease is included.

(2) Expected Grade 4 non-hematologic toxicity (CTCAE v3.0 adverse events other than the blood/bone marrow group).

(3) Unexpected Grade 3adverse events: Grade 3 adverse events are not recorded in the

12.1 expected adverse events.

(4) Data on COVID-19 diagnoses (suspected and confirmed) will be collected as routine adverse events, for the purpose of identifying cases in the future as needed for ancillary research proposals in development.

(5) Other significant medical events: adverse events that the study group deems cause Important and potentially permanent, significant impact on their offspring (MDS myelodysplastic syndrome, except for secondary cancer) Adverse events among above (2)-(5), determined to have a causal relationship (any of definite, probable, possible)

with the study regimen are regular reporting objects.

12.4.2 Reporting Procedure

12.4.2.1 Emergency Reporting

- In case of any adverse event on emergency study reporting objects, the doctor in charge will quickly report it to the Research Responsible Person of the research participating hospitals. When the Research Responsible Person of the hospital cannot be contacted, the coordinator or the doctor in charge of the hospital must assume the responsibility on behalf of the Research Responsible Person of the hospital.
- First Reporting: Within 72 hours after the occurrence of adverse events, the Research Responsible Person of the hospital should complete the "AE/AR/ADR first emergency report" and send it to the Research Committee by email and telephone.
- Second Reporting: The Research Responsible Person of each research participating hospital completes the "AE/AR/ADR Report" and a more detailed case information report (A4 format), and then faxes the two reports to the Research Committee within 15 days after the occurrence of adverse events. If any autopsy examination, the autopsy result report should be submitted to the Research Committee.

12.4.2.2 General Reports

- The Research Responsible Person of each research participating hospital completes the "AE/AR/ADR report", and then faxes it to the Research Committee within 15 days after the occurrence of adverse events.

12.5 Review of Efficacy and Safety Evaluation Committee

The Efficacy and Safety Evaluation Committee reviews and discusses the report in accordance with the procedures recorded in the *Clinical Safety Information Management Guideline*, and makes recommendations in writing for the Research Responsible Person, including whether to continue to include study objects or to modify the study protocol.

13 Ethical Considerations

13.1 Responsibilities of researchers

The investigators are responsible for the conduction of this study at their centers.

The investigators will ensure the implementation of this study in accordance with the study protocol and in compliance with the Declaration of Helsinki, as well as domestic and international ethical guiding principles and applicable regulatory requirements. It is specially noted that, the investigators must ensure that only subjects providing informed consent can be enrolled in this study.

13.2 Information and Informed Consent of Subjects

An unconditional prerequisite for subjects to participate in this study is his/her written informed consent. The written informed consent of subjects participating in this study must be given before study-related activities are conducted.

Therefore, before obtaining informed consent, the investigators must provide sufficient information to the subjects. In order to obtain the informed consent, the investigators will provide the information page to subjects, and the information required to comply with the applicable regulatory requirements. While providing written information, the investigators will orally inform the subjects of all the relevant circumstances of this study. In this process, the information must be fully and easily understood by non-professionals, so that they can sign the informed consent form according to their own will on the basis of their full understanding of this study.

The informed consent form must be signed and dated personally by the subjects and investigators. All subjects will be asked to sign the informed consent form to prove that they agree to participate in the study. The signed informed consent form should be kept at the research center where the investigator is located and must be properly safe kept for future review at any time during audit and inspection throughout the inspection period. Before participating in the study, the subjects should provide a copy of signed and dated informed consent form.

At any time, if important new information becomes available that may be related to the consent of the subjects, the investigators will revise the information pages and any other written information which must be submitted to the IEC/IRB for review and approval. The revised information approved will be provided to each subject participating the study. The researchers will explain the changes made to the previous version of ICF to the subjects

13.3 Identity and Privacy of Subjects

After obtaining an informed consent form, each selected subject is assigned a subject number (Allocation Number). This number will represent the identity of the subject during the entire study and for the clinical research database of the study. The collected data of subjects in the study will be stored in the ID.

Throughout the entire study, several measures will be taken to minimize any breaches of personal information, including: 1) only the investigators will be able to link to the research data of the subjects to themselves through the identify table kept at the research center after authorization; 2) during onsite auditing of raw data by the supervisors of this study, as well as relevant inspection and inspection visits by the supervision departments, the personnel engaging in the above activities may view the original medical information of subjects that will be kept strictly confidential.

Collection, transmission, handling and storage of data on study subjects will comply with the data protection and privacy regulations. This information will be provided to the study subjects when their informed consent is being obtained for treatment procedures in accordance with national regulations.

13.4 Independent Ethics Committee or Institutional Review Committee

Before beginning the study, the Research Center will be responsible for submitting the study protocol and relevant documents (informed consent form, subject information page, CRF, and other documents that may be required) to the Independent Ethics Committee (IEC)/ Institutional Review Board (IRB) to obtain their favorable opinion/approval. The favorable opinions/approval documents of the IEC/IRB will be archived in the research center folders of the investigators.

Before beginning the study at the center, the investigators must obtain written proof of favorable opinions/approval by the IEC/IRB, and should provide written proof of the date of the favorable opinions/approval meeting, written proof of the members presenting at the meeting and voting members, written proof of recording the reviewed study, protocol version and Informed Consent Form version, and if possible, a copy of the minutes.

In case of major revisions to this study, the amendment of the study protocol will be submitted to the IEC/IRB prior to performing the study. In the course of the study, the relevant safety information will be submitted to the IEC/IRB in accordance with national regulations and requirements.

13.5 Supervising

The research approach of the authorities and any associated files (such as the research protocol, subjects' informed consent) will be in accordance with the requirements of the ethical review board of biomedical research involving humans (Trial) (2007) and the applicable Chinese laws and regulations. Studies should provide the main references or inform the ethics review guidance advisory organization of the provincial health administrative department in the province the research center is in.

14 Organizations and Responsibilities of Study

14.1 Research Committee

- Responsible for developing study protocol, auditing eligibility for inclusion and guiding the interpretation of informed consent; also responsible for the collection of adverse event reports, guiding the clinical diagnosis and treatment of such events and the emergency intervention of serious adverse events.
- Person in Charge of Research Committee: Changming Huang （Department of Gastric Surgery, Fujian Medical University Union Hospital）

  Add: Department of Gastric Surgery, Fujian Medical University Union Hospital, No.29 Xinquan Road, Fuzhou 350001, Fujian Province, China.;Post code:350001;Tel:0591-83357896-8011;Fax:0591-83363366;Mobile:13805069676;

  E-mail：hcmlr2002@163.com
- Chief Statistical Expert of Research Committee: Hu Zhijian （Department of Preventive Medicine statistics, School of Public health, Fujian Medical University）

14.2 Efficacy and Safety Evaluation Committee

Responsible for the supervision/monitoring of treatment safety and efficacy of this study.

Person in Charge of Efficacy and Safety Evaluation Committee: Changming Huang （Department of Gastric Surgery, Fujian Medical University Union Hospital）

14.3 Independent Ethics Committee/Institutional Review Board (IEC/IRB)

Responsible for evaluating this study to determine if risks to which subjects are exposed have been duly minimized and whether these risks are reasonable compared

to expected benefits.

The independent Ethics Committee/Institutional Review Board (IEC/IRB) at the location of each research participating center is responsible for the ethics review of all research participating units.

15 References

[1] Parkin, DM and Muir CS. Cancer Incidence in Five Continents. Comparability and quality of data. IARC Sci Publ, 1992(120): 45-173.

[2] Bozzetti, F., et al., Subtotal versus total gastrectomy for gastric cancer: five-year survival rates in a multicenter randomized Italian trial. Italian Gastrointestinal Tumor Study Group. Ann Surg, 1999. 230(2): p. 170-8.

[3]. Jentschura, D., et al., Quality-of-life after curative surgery for gastric cancer: a comparison between total gastrectomy and subtotal gastric resection. Hepatogastroenterology,
1997. 44(16): p. 1137-42.

[4] Kitano S, Iso Y, Moriyama M, et al. Laparoscopy-assisted Billroth Ⅰ gastrectomy[J]. Surg Laparosc Endosc, 1994,4(2): 146-148.

[5] Kitano S, Shiraishi N, Fujii K, et al. A randomized controlled trial comparing open vs laparoscopy-assisted distal gastrectomy for the treatment of early gastric cancer: an interim report[J]. Surgery, 2002,131(1 Suppl):S306-S311.

[6] Lee JH, Han HS, Lee JH. A prospective randomized study comparing open vs laparoscopy-assisted distal gastrectomy in earlygastriccancer:earlyresults[J]. Surg Endosc, 2005,19(2): 168-173.

[7] Huscher CG, Mingoli A, Sgarzini G, et al. Laparoscopic versus open subtotal gastrectomy for distal gastric cancer: five-year results of a randomized prospective trial[J]. Ann Surg, 2005,241(2):232-237.

[8] Hayashi H, Ochiai T, Shimada H, et al. Prospective randomized study of open versus laparoscopy-assisted distal gastrectomy with extraperigastric lymph node dissection for early gastric cancer[J]. Surg Endosc, 2005,19(9):1172-1176.

[9] Kim YW, Baik YH, Yun YH, et al. Improved quality of life outcomes after laparoscopy-assisted distal gastrectomy for early gastric cancer: results of a prospective randomized clinical trial[J]. Ann Surg, 2008,248(5):721-727.

[10] Kim HH, Hyung WJ, Cho GS, et al. Morbidity and mortality of laparoscopic gastrectomy versus open gastrectomy for gastric cancer: an interim report--a phaseIII multicenter, prospective, randomized Trial(KLASS Trial)[J]. Ann Surg, 2010,251(3):417-420.

[11] Song J, Lee HJ, Cho GS, et al; Korean Laparoscopic Gastrointestinal Surgery Study (KLASS) Group. Recurrence following laparoscopy-assisted gastrectomy for gastric cancer: a multicenter retrospective analysis of 1,417 patients [J]. Ann Surg Oncol, 2010,17(7):1777-1786.

[12] Scatizzi M, Kroning KC, Lenzi E, et al. Laparoscopic versus open distal gastrectomy for locally advanced gastric cancer: a case-control study[J]. 2011,63:17-23. Updates Surg, 2011,63 (1):17-23.

[13] Kojima K, Yamada H, Inokuchi M, et al. Current status and evaluation of laparoscopic surgery for gastric cancer [J]. Nihon Geka Gakkai Zasshi, 2006,107(2):77-80.

[14] Hu Y, Huang C, Sun Y, et al. Morbidity and Mortality of Laparoscopic Versus Open D2 Distal Gastrectomy for Advanced Gastric Cancer: A Randomized Controlled Trial. J Clin Oncol. 2016,34(12):1350-1357.

[15] Cadière GB, Himpens J, Germay O, et al. Feasibility of robotic laparoscopic surgery: 146 cases[J]. World J Surg, 2001,25(11):1467-1477.

[16] Patriti A, Ceccarelli G, Ceribelli C, et al. Robot-assisted laparoscopic management of cardia carcinoma according to Siewert recommendations [J]. Int J Med Robot, 2011,7(2):170-177.

[17] Oleynikov D. Robotic surgery[J]. Surg Clin North Am, 2008,88(5):1121-1130, viii.

[18] Lanfranco AR, Castellanos AE, Desai JP, et al. Robotic surgery: a current perspective[J]. Ann Surg, 2004,239(1):14-21.

[19] Kaushik D, High R, Clark CJ, et al. Malfunction of the Da Vinci robotic system during robot-assisted laparoscopic prostatectomy: an international survey [J]. J Endourol, 2010,24(4):571-575.

[20] Hashizume M, Shimada M, Tomikawa M, et al. Early experiences of endoscopic procedures in general surgery assisted by a computer-enhanced surgical system[J]. Surg Endosc, 2002, 16(8):1187-1191.

[21] Liu Guoxiao, Shen Weisong, Chen Lin, Wei Bo. Robotic versus laparoscopic

gastrectomy for gastric cancer: a meta-analysis. Chin J Gastrointest Surg, 2016,19(3):328-333.

[22] Kim HI, Han SU, Yang HK, et al. Multicenter Prospective Comparative Study of Robotic Versus Laparoscopic Gastrectomy for Gastric Adenocarcinoma. Ann Surg. 2016,263(1):103-109.

16 Annex

16.1 Informed Consent Form

Final protocol

Randomized Controlled Trials on Clinical Outcomes of Robotic versus Laparoscopic Distal Gastrectomy for Gastric Cancer (FUGES-011)

Study protocol

Bidding party: Fujian Medical University Union Hospital

Principle Investigator:

Prof. Chang-Ming Huang, M.D.

Department of Gastric Surgery, Fujian Medical University Union Hospital,

Address: No. 29 Xinquan Road, Fuzhou 350001 Fujian Province, China.

Telephone: +86-591-83363366, Fax: +86-591-83363366

No. of edition: V2.0

The date of the edition: 2023.05

Summary

Scenario Title	**Randomized Controlled Trials on Clinical Outcomes of Robotic versus Laparoscopic Distal Gastrectomy for Gastric Cancer (FUGES-011)**
Scenario Version	V2.0
Sponsor	Chang-Ming Huang
Research Center	Fujian Medical University Union Hospital
Indications	Patients with potentially resectable gastric adenocarcinoma (cT1-4a, N0/+, M0) located in the middle and lower third of the stomach expected to undergo distal gastrectomy.
Purpose of research	To investigate the safety, feasibility and long-term outcome of robotic distal gastrectomy versus laparoscopic distal gastrectomy for gastric cancer
Research design	Single center, prospective, open-label, randomized controlled, non-inferior test
ClinicalTrials .gov Identifier	NCT03313700
Case grouping	Group A (Study Group): Robotic distal gastrectomy Group (RDG group)<br>Group B (Control Group): Laparoscopic distal gastrectomy Group (LDG group)
The basis for determining the sample size	This study is a non-inferior test (bilateral), whose primary outcome measure is 3-year disease free survival. According to the previous study results and related literature reports, the 3-year DFS rate for the LDG group was 82.3%. According to an α of 0.025, a power of 90%, and a margin delta of 16%, we determined that at least 120 patients should be included each group. Considering an expected dropout rate of 20%, it was determined that each group needed at least 150 patients, for a total of 300 cases.

Inclusion criteria	● Age from 18 to 75 years (not including 18 and 75 years old)<br>● Primary gastric adenocarcinoma (papillary, tubular, mucinous, signet ring cell, or poorly differentiated) confirmed pathologically by endoscopic biopsy<br>● Clinical stage tumor T1-4a (cT1-4a), N-/+, M0 at preoperative evaluation according to the American Joint Committee on Cancer (AJCC) Cancer Staging Manual Eighth Edition<br>● Expected to undergo distal gastrectomy with D1+/D2 lymph node dissction to obtain R0 resection sugicall results<br>● Performance status of 0 or 1 on Eastern Cooperative Oncology Group scale (ECOG)<br>● American Society of Anesthesiology score (ASA) class I, II, or III<br>● Written informed consent
Exclusion criteria	● Women during pregnancy or breast-feeding<br>● Severe mental disorder<br>● History of previous upper abdominal surgery (except laparoscopic cholecystectomy)<br>● History of previous gastrectomy, endoscopic mucosal resection or endoscopic submucosal dissection<br>● Multiple primary gastric cancer<br>● Enlarged or bulky regional lymph node diameter over 3cm by preoperative imaging<br>● History of other malignant disease within past five years<br>● History of previous neoadjuvant chemotherapy or radiotherapy<br>● History of unstable angina or myocardial infarction within past six months<br>● History of cerebrovascular accident within past six months<br>● History of continuous systematic administration of corticosteroids within one month<br>● Requirement of simultaneous surgery for other disease<br>● Emergency surgery due to complication (bleeding, obstruction or

	perforation) caused by gastric cancer<br>● FEV1 < 50% of predicted values
Rejection criteria	● M1 tumor confirmed intraoperatively or postoperatively: distant metastasis only found by intraoperative exploration or postoperative pathological biopsy or a positive postoperative peritoneal lavage cytology examination<br>● Patients intraoperatively/postoperatively confirmed as T4b<br>● Patients intraoperatively confirmed as unable to complete D2 lymph node dissection/R0 resection due to tumor: unable to complete R0 resection due to regional lymph node integration into a mass or surrounded with important blood vessels, which cannot be resected;<br>● Patients converted to total gastrectomy intraoperatively;<br>● Patients requiring simultaneous surgical treatment of other diseases;<br>● Sudden severe complications during the perioperative period (intolerable surgery or anesthesia), which renders it unsuitable or unfeasible to implement the study treatment protocol as scheduled;<br>● Patients confirmed to require emergency surgery by attending physicians due to changes in the patient's condition after inclusion in this study;<br>● Patients who voluntarily quit or discontinue treatment for personal reasons at any stage after inclusion in this study;<br>● Treatment implemented is proven to violate study protocol.
Intervention	● Implement robotic (group A) or laparoscopic (group B) distal gastrectomy with D1+/D2 lymphadenectomy according to the Japanese gastric caner treatment guidelines 2014 (4th Edition)
Outcome Measures	**Primary Outcome Measures**：<br>● 3-year disease free survival rate<br>**Secondary Outcome Measures**：<br>● 3-year overall survival rate<br>● 3-year recurrence pattern

	● Overall postoperative morbidity rates<br>● Intraoperative morbidity rates<br>● Overall postoperative serious morbidity rates<br>● Number of retrieved lymph nodes<br>● Noncompliance rate of lymphadenectomy<br>● Time to first ambulation<br>● Time to first flatus<br>● Time to first liquid diet<br>● Time to first soft diet<br>● Duration of postoperative hospital stay<br>● The variation of weight<br>● The variation of cholesterol<br>● The variation of album<br>● The variation of white blood cell count<br>● The variation of hemoglobin<br>● Hospitalization expenses<br>● Operation time
Statistical consideratio ns	All data analyses will be performed using the SAS statistical package (version 9.2, SAS Institute, Cary, North Carolina, USA).<br>The noninferiority analysis for the primary endpoint of 3-year disease-free survival will be conducted, while the test method of difference for other outcomes. All the statistical tests were tested by two sides. A p-value <0.05 is considered statistically significant. The confidence interval of the parameters is estimated with a 95% confidence interval. Baseline data and validity analyses will be conducted on a modified intent-to-treat (MITT) basis, and the primary endpoint will also be analyzed on a per-protocol (PP) basis, with the MITT analysis results prevailing. SAP analysis is used for safety assessment, and this study does not fill in missing values. Normally distributed continuous variables will be presented as mean and standard deviation and compared using the t-test if normally distributed, or as median and interquartile range and compared using the Wilcoxon rank-sum test if non-normally distributed; while categorical data will be

	presented as number and percentages and compared using the Pearson χ2 test or the Fisher exact test, as appropriate. Survival data will be analyzed using the Kaplan-Meier method and Cox's proportional hazards model. Sensitivity analysis is used for extreme outlier data. The central effect analysis and subgroup analysis are conducted according to the specific situation. Interim analysis will not be conducted in this study.

4. Research background

In the worldwide, the incidence of gastric cancer is the fourth most common malignant tumor, and the second leading cause of cancer-related death. Although the incidence of gastric cancer has a downward trend in western countries, it still maintains a high level in East Asia. Radical gastrectomy is the only way to cure gastric cancer. In China, Japan, Korea and other East Asian countries, the primary lesions of gastric cancer are mostly located in the middle and lower third of the stomach [1]. Previous studies have shown that if the proximal resection margin is far enough, the long-term oncological effect of total gastrectomy and distal gastrectomy is equivalent. However, the quality of life of patients after distal gastrectomy is higher than those after total gastrectomy [2,3]. Therefore, distal gastrectomy is the most widely used surgical approach of gastrectomy.

Since the first laparoscopic gastrectomy was reported by Kitano et al [4] in 1994, it has been widely recognized internationally during the recent 20 years. A mount of randomize controlled trials have confirmed that laparoscopic gastrectomy has the advantages of fast recovery and less complications when compare with open gastrectomy. Moreover, the long-term survival of laparoscopic radical gastrectomy was comparable with laparotomy [11-13]. In early gastric cancer, laparoscopic distal gastrectomy has become a standard surgical approach. In addition, it has also been reported that laparoscopic radical gastrectomy is also feasible in advanced gastric cancer [14]. Development has taken place in the field of laparoscopic gastrectomy these decades, however, the traditional laparoscopic surgery has some limitations in fine steps, visual field and so on.

Because of the limited motion of laparoscopic instruments, poor visual field and, two-dimensional plane without spatial sense, it may cause vascular bleeding when perforoming lymph node dissection, due to the complex anatomical structure and compact proximity of blood vessels around the stomach. Additionally, as reported, both operation time and the learning curve are long. Especially in the patients with obesity, large anterior and posterior diameter and small costal arch angle, the difficulty will be more protruding and the laparoscopic operation will be seriously affected in the deep and narrow abdominal space.

In order to overcome the limitations of laparoscopic maneuvers, Da Vinci robotic system emerged. As an advanced laparoscopic system, robot solves many shortcomings of conventional laparoscopy with its unique advantages which are mainly reflected as following: (1) High-definition three-dimensional magnification imaging can better display small anatomical structures. It is easier to expose perigastric vessels and reduce the difficulty of lymph node dissection and the amount of intraoperative blood loss. (2) The simulated "wrist" with 7 degrees of freedom greatly improves the flexibility, especially in the difficult suture operation.

In 1997, Cadiere successfully completed the robot-assisted cholecystectomy firstly [15]. Nowadays, robotic surgery system has been widely used in the fields of urology, hepatobiliary and cardiovascular surgery and gynecology [16-19]. In the field of gastrectomy, Hashizume et al. [20] reported robotic gastrectomy for the first time in 2002. Since then, more and more reports about the safety and feasibility of robotic surgery system in the treatment of gastric cancer, especially in Asia. Liu et al. [21] conducted a meta analysis combined the results of 16 studies showing that compared with laparoscopic surgery, robotic surgery can achieve radical resection of gastric cancer, and has the advantages of less blood loss and more lymph nodes retrieved. Most studies, however, are still retrospective, and no prospective randomized controlled trial on robotic gastrectomy was reported so far. In addition, due to the high cost of the robotic surgery system, robotic gastrectomy can only be performed in high-volume hospitals, and the benefit for patients is still controversial. Kim et al. [22] reported a prospective non-randomized controlled study in 11 centers in 2015. The results showed that although robotic gastrectomy reduce the intraoperative blood loss, it takes longer operation time with higher cost and the short-term outcomes of robotic gastrectomy are not superior to traditional laparoscopic gastrectomy. In addition, that study is a non-randomized controlled trial, and there is a deviation in the baseline of patients in both arms. For example, patients in the laparoscopic group were less likely to undergo D2 lymph node dissection with earlier tumor stage. And most of the participating surgeons were experts in laparoscopic gastrectomy but were less experienced in robotic gastrectomy (the median number of robotic gastrectomy performed by the participating surgeons annually was only 5).

Therefore, based on the mature technology of traditional laparoscopic and

robotic gastrectomy, this prospective randomized controlled trial conducted at a simultaneous, large-scale center focused on patients with potentially resectable gastric adenocarcinoma (cT1-4a, N0/+, M0) located in the middle and lower third of the stomach to evaluate the short- and long-term effect of robotic distal gastrectomy.

5. Objective

The purpose of the randomized controlled trial is to investigate the safety, feasibility and long-term outcome of robotic distal gastrectomy versus laparoscopic distal gastrectomy for gastric cancer.

6. Research design

Single center, prospective, open-label, parallel assignment, randomized controlled.

3.1 Single center

Department of gastric surgery in Fujian Medical University Union Hospital

3.2 Case group

Group A (Study Group): Robotic distal gastrectomy Group (RDG group)

Group B (Control Group): Laparoscopic distal gastrectomy Group (LDG group)

3.3 Estimate Sample Size

This study is a non-inferior test (bilateral), whose primary outcome measure is 3-year disease free survival. According to the previous study results and related literature reports, the projected 3-year DFS rate for the LDG group was 82.3%. Based on an α of 0.025, a power of 90%, and a margin delta of 16%, we determined that at least 120 patients should be included each group. Considering an expected dropout rate of 20%, a total of 300 patients were needed. For both drop-ins and drop-outs, observation time will be censored at the time of drop-in or drop-out.

3.4 Blind method： This research adopts an open design

3.5 Research cycle

Estimated enrollment cycle: complete enrollment within 2 years

Follow-up period: begin at the enrollment of the first case and end 3 years after the enrollment of the last case.

Estimated time: 2017.09-2019.09 （to complete enrollment） - 2022.09 （to complete follow-up）

Actually time: 2017.09-2020.01（to complete enrollment）- 2023.01（to complete follow-up）. Follow-up period changed to 3 years after the final participant's randomization date.

3.6 Randomization

SAS 9.2 program was used to generate serial numbers from 001 to 300 that corresponds to the intervention assignment. Before the surgery, the data manager extracted the numbers and then randomly assigned patients in a 1:1 ratio to either the LDG group or the RDG group. Written informed consent was obtained from patients.

5. Study objects

All patients who meet the inclusion criteria and not conform to the exclusion criteria are qualified for this study.

4.1 Inclusion criteria

(1) Age from 18 to 75 years (not including 18 and 75 years old)

(2) Primary gastric adenocarcinoma (papillary, tubular, mucinous, signet ring cell, or poorly differentiated) confirmed pathologically by endoscopic biopsy

(3) Clinical stage tumor T1-4a (cT1-4a), N-/+, M0 at preoperative evaluation according to the American Joint Committee on Cancer (AJCC) Cancer Staging Manual Eighth Edition

(4) Expected to undergo distal gastrectomy and D1+/D2 lymph node dissection to obtain R0 surgical results.

(5) Performance status of 0 or 1 on the ECOG (Eastern Cooperative Oncology Group) scale

(6) ASA class I to III

(7) Written informed consent

4.2Exclusion criteria

(1) Women during pregnancy or breast-feeding

(2) Severe mental disorder

(3) History of previous upper abdominal surgery (except for laparoscopic cholecystectomy)

(4) History of previous gastric surgery (including ESD/EMR for gastric cancer)

(5) Multiple primary gastric cancer

(6) Enlarged or bulky regional lymph node diameter over 3cm by preoperative imaging

(7) History of other malignant disease within past five years

(8) History of previous neoadjuvant chemotherapy or radiotherapy

(9) History of unstable angina or myocardial infarction within the past six months

(10) History of cerebrovascular accident within past six months

(11) History of continuous systematic administration of corticosteroids within one month

(12) Requirement of simultaneous surgery for another disease

(13) Emergency surgery due to complications (bleeding, obstruction or perforation) caused by gastric cancer

(14) FEV1 < 50% of the predicted values

4.3 Rejection criteria

(1) M1 tumor confirmed intraoperatively or postoperatively: distant metastasis only found by intraoperative exploration or postoperative pathological biopsy or a positive postoperative peritoneal lavage cytology examination

(2) Patients intraoperatively/postoperatively confirmed as T4b

(3) Patients intraoperatively confirmed as unable to complete D2 lymph node dissection/R0 resection due to tumor: unable to complete R0 resection due to regional lymph node integration into a mass or surrounded with important blood vessels, which cannot be resected;

(4) Patients converted to total gastrectomy intraoperatively;

(5) Patients requiring simultaneous surgical treatment of other diseases;

(6) Sudden severe complications during the perioperative period (intolerable surgery or anesthesia), which renders it unsuitable or unfeasible to implement the study treatment protocol as scheduled;

(7) Patients confirmed to require emergency surgery by attending physicians due to changes in the patient's condition after inclusion in this study;

(8) Patients who voluntarily quit or discontinue treatment for personal reasons at any stage after inclusion in this study;

(9) Treatment implemented is proven to violate study protocol.

4.4 Case screening

(1) When Patients admitted to hospital should meet the following criteria: age between 18 and 75 years old; performance status of 0 or 1 on the ECOG scale; none-pregnant or no lactating women; not suffering from a severe mental disorder; no history of previous upper abdominal surgery (except for laparoscopic cholecystectomy); no history of previous gastric surgery (including ESD/EMR for gastric cancer); no history of other malignant disease within the past five years; no history of unstable angina or myocardial infarction within the past six months; no history of continuous systematic administration of corticosteroids within one month; no requirement of simultaneous surgery for another disease; FEV1≥50% of the predicted values; no history of a cerebrovascular accident within the past six months.

(2) Endoscopic examination of the primary lesion in the patient (recommended endoscopic ultrasound endoscopy, EUS) and histopathological biopsy showed gastric adenocarcinoma (papillary adenocarcinoma [pap], tubular adenocarcinoma [tub], mucinous adenocarcinoma [muc], signet ring cell carcinoma [sig], and poorly differentiated adenocarcinoma [por]). Total abdominal CT was performed on the patient, and no enlarged lymph nodes (maximum diameter ≥ 3 cm) were found in the periplasmic area, including significant enlargement or merging of the No. 10 lymph nodes into a group or local invasion/distance metastasis. No obvious tumor infiltration was found in the spleen and spleen vessels.

(3) Patient is explicitly diagnosed with middle and/or lower third gastric cancer, has a preoperative staging assessment of T1-4a, N0-3, M0 and is expected to undergo distal gastrectomy with D1+/D2 lymph node dissection to obtain R0 surgical results.

(4) Patients do not require neoadjuvant chemoradiotherapy or chemotherapy and the attending doctor does not recommend that they receive neoadjuvant chemoradiotherapy or chemotherapy.

(7) ASA class I to III.

(8) No requirement for emergency surgery.

(7) At this point the patient becomes a potential selected case and enters the 9.1

case selection procedure.

5. Outcome Measures

5.1 Primary Outcome Measures

- 3-year disease free survival rate

5.2 Secondary Outcome Measures

- 3-year overall survival rate
- 3-year recurrence pattern
- overall postoperative morbidity rates
- intraoperative morbidity rates
- overall postoperative serious morbidity rates
- number of retrieved lymph nodes
- the noncompliance rate of lymphadenectomy
- time to first ambulation
- time to first flatus
- time to first liquid diet
- time to first soft diet
- duration of postoperative hospital stay
- the variation of weight
- the variation of cholesterol
- the variation of album
- the variation of white blood cell count
- the variation of hemoglobin
- hospitalization expenses
- operation time

6．Diagnostic criteria for this study

(1) The AJCC-8th TNM tumor staging system will be used for this study.

(2) Diagnostic criteria and classification of gastric cancer: According to the histopathological international diagnostic criteria, classification will be divided into papillary adenocarcinoma (pap), tubular adenocarcinoma (tub), mucinous

adenocarcinoma (muc), signet ring cell carcinoma (sig), and poorly differentiated adenocarcinoma (por).

- The definition of middle and lower third gastric cancer:

Acoording to Japanese classification of gastric carcinoma (4rd English edition), the stomach is anatomically divided into three portions, the upper (U), middle (M), and lower (L) parts, by the lines connecting the trisected points on the lesser and greater curvatures (Fig. 1). Middle and lower third gastric cancer is described as the center of tumor located in the middle and lower third part of stomach, including M, L, ML.

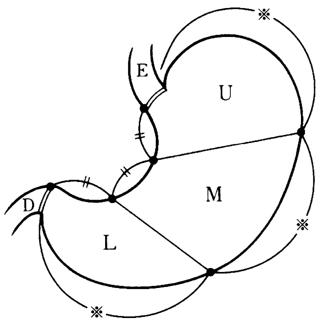

Fig. 1. The three portions of the stomach. *U* upper third, *M* middle third, *L* lower third, *E* esophagus, *D* duodenum

7. Qualifications of the participated Surgeons

7.1 Basic principle

All candidate surgeons in our study met the following criteria:

Performed at least 300 laparoscopic radical gastrectomies and at least 50 robotic radical gastrectomies.

Pass the blind surgical video examination.

7.2 Checklist for determination of success about D2 lymphadenectomy

Scoring Method for D2 Lymph Node Dissection	Complete Incomplete None

	10	5	0
1. Properly full omentectomy	□	□	□
2. Ligation of left gastroepiploic artery at origin	□	□	□
3. Ligation of right gastroepiploic artery at origin	□	□	□
4. Full exposure of common hepatic artery	□	□	□
5. Ligation of right gastric artery at origin	□	□	□
6. Exposure of portal vein	□	□	□
7. Exposure of splenic artery to branch of posterior gastric artery	□	□	□
8. Identification of splenic vein	□	□	□
9. Ligation of left gastric artery at origin	□	□	□
10. Exposure of gastroesophageal junction	□	□	□

1. Properly full omentectomy

   a. Omentectomy was performed close to transverse colon

   b. Omentectomy was performed from hepatic flexure to splenic flexure

   c. Anterior layer of transverse colonic mesentery and pancreatic anterior peritoneum was dissected.

2. Ligation of left gastroepiploic artery at origin

3. Ligation of right gastroepiploic artery at origin

4. Full exposure of common hepatic artery

   a. More than half of anterior part in the common hepatic artery were exposed.

5. Ligation of right gastric artery at origin

6. Exposure of portal vein

7. Exposure of splenic artery to branch of posterior gastric artery

a. More than half of anterior part in splenic artery was exposed.

b. Splenic artery was exposed from celiac trunk to posterior gastric artery

8. Identification of splenic vein

9. Ligation of left gastric artery at origin

10. Exposure of gastroesophageal junction

a. Anterior and right side of the abdominal esophagus were exposed.

- D2 lymphadenectomy was accepted if all randomly assigned three investigators rated 85 points and more regarding checklists in unedited video review.

8. End point and definition of related result determination

8.1 Disease-free survival

Disease-free survival is calculated from the day of surgery to the day of recurrence or death (when the specific date of recurrence of the tumor is unknown, the ending point is the date of death due to tumor causes）. In the event that neither death nor recurrence of the tumor are observed, the end point is the final date that a patient is confirmed as relapse-free.（The final date of DFS: the last date of the outpatient visit day or the date of acceptance of the examination）.（Follow-up cycle and required examinations are shown in the follow-up process 9.5.3）

8.2 Overall survival time

The overall survival is calculated from the day of surgery until death or until the final follow-up date, whichever occurs first. For survival cases, the end point is the last date that survival was confirmed. If loss to follow-up occurred, the end point is the final date that survival could be confirmed.

8.3 Definition of recurrence and recurrence date

The following situations are regarded as "recurrence" and should be recorded as the evidence of "recurrence" in the CRF.

(3) Recurrence identified by any one image examination (X-ray, ultrasound, CT, MRI, PET-CT, endoscope, etc.) and, if there are a variety of imaging examinations, results without contradiction determined "recurrence". The earliest date that the recurrence is found is defined as the "recurrence date".

(4) For cases that lack the use of imaging or a pathological diagnosis, the date we diagnose the occurrence of clinical recurrence based on clinical history and physical examination is defined as the “recurrence date”.

(3) For cases without imaging or clinical diagnosis but with a cytology or tissue biopsy pathological diagnosis of recurrence, the earliest date confirmed by cytology or biopsy pathology is considered the "recurrence date".

(4) A rise in CEA or other associated tumor markers alone could not be diagnosed as a relapse.

8.4 Incidence of surgical complications

8.4.1 Incidence of intraoperative complications

The number of all patients treated with surgery as the denominator and the number of the patients with any intraoperative complications as the numerator are used to calculate the proportions. The criteria for the intraoperative complications refer to the descriptions of intraoperative complications in the observation project (in 9.3.3).

8.4.2 Incidence of postoperative complications

The number of all patients treated with surgery as the denominator and the number of the patients with any postoperative complications as the numerator are used to calculate the proportions.

Incidence of overall postoperative complications: The postoperative complication criteria refer to short-term complications after surgery in the postoperative observation project (see 9.4.5). The time is defined as within 30th after surgery, or the first discharge time if the days of hospital stay more than 30 days.

Incidence of postoperative major complications: The standard for postoperative major complications refers to the short-term complications in the postoperative

observation project (see 9.4.5). According to the Clavien–dindo grade, IIIA level and above for serious complications, and when multiple complications occur simultaneously, the highest ranked complication is the subject.

8.4.3 Mortality

- The number of all the patients receiving surgery as the denominator and the number of the patients in any of the following situations as the numerator are used to calculate proportions. This proportion indicated the operative mortality ratio.
- Situations: patients whose death was identified according to documented intraoperative observation items, including patients who die within 30 days after the surgery (including 30 days) regardless of the causality between the death and the surgery, and patients who die more than 30 days after the surgery (whose death is proved to have a direct causal relationship with the first operation).

8.5 Number of lymph node dissection

The sum of retrieved lymph nodes in each station.

8.6 Determination of surgical outcomes

8.6.1 Operative time: from skin incision to the skin being sutured

8.6.2 Postoperative recovery indexes

8.6.2.1 Time to ambulation, flatus, recovery of liquid diet and semi-liquid diet.

- During the day of surgery to the first discharge, the initial time to ambulation, flatus, liquid diet and semi-liquid diet during the postoperative hospitalization is recorded by hour.
- Flatus on the operation day should be excluded.
- If flatus or resumption of liquid and semi-liquid diet does not occur before hospital discharge, the discharge time should be recorded as the corresponding time.
- The initial time to ambulation, flatus, liquid diet and semi-liquid diet should be recorded according to patients' reports.

8.6.2.2 The maximum temperature

The highest value of body temperature measured at least 3 times a day from the first day to the eighth day after operation is documented.

8.6.3 Laparoscopic / Robotic surgery completion ratio

The number of all patients treated with laparoscopic/robotic surgery as the

denominator and the number of the patients without conversion to laparotomy as the numerator are used to calculate the ratio.

8.6.4 Percentage of conversion to laparotomy

Among all the patients who underwent surgery, the number of patients planning to receive a laparoscopic surgery per protocol is used as the denominator, while the number of the patients who receive a conversion to open surgery is considered the numerator. The proportion calculated is regarded as the rate of transfer laparotomies. In this study, if the length of the auxiliary incision is more than 10 cm, it is considered a conversion to open surgery.

9. Standard operating procedures (SOP)

9.1 Case selection

9.1.1 Selection assessment items

Clinical examination data of patients conducted from hospital admission to enrollment into this study (time period is usually 2 weeks) will be considered baseline data, and must include:

(1) Systemic status: ECOG score, height, weight

(2) Peripheral venous blood: Hb、RBC、WBC、LYM、NEU、NEU%、PLT、MONO

(3) Blood biochemistry: albumin, prealbumin, total bilirubin, indirect bilirubin, direct bilirubin, AST, ALT, creatinine, urea nitrogen, Total cholesterol, triglycerides, fasting glucose, potassium, sodium, chlorine, calcium

(4) Serum tumor markers: CEA、CA19-9、CA72-4、CA12-5、AFP

(5) Full abdominal (slice thickness of 10mm or less, in case of allergy to the contrast agent, CT horizontal scanning is allowed only)

(6) Upper gastrointestinal endoscopic ultrasonography (EUS) and biopsy, if no EUS, select ordinary upper gastrointestinal endoscopy and biopsy instead

(7) Chest X-ray (AP and lateral views): cardiopulmonary conditions

(8) Resting 12-lead ECG

(9) Respiratory function tests: FEV1, FVC

9.1.2 Selection application

For cases that meet all inclusion criteria and none of the exclusion criteria, talk to

patients and their families and sign informed consent. Application and confirmation of eligibility should be completed preoperatively; postoperative applications will not be accepted.

9.2 Preoperative management

After the eligibility is obtained, surgery should be performed within two weeks (including the 14th day)

- In case of any deterioration of the clinical conditions from the selection time to the expected day of surgery, whether to undergo an elective surgery as planned should be decided in accordance with the judgment of the doctor in charge; if an emergency surgery is required, the case should be withdrawn from PP set according to 4.3 Withdrawal Criteria;
- For patients with nutritional risks, preoperative enteral/parenteral nutritional support is allowed
- For elderly, smokers, high-risk patients with diabetes, obesity and chronic cardiovascular/cerebrovascular or thromboembolic past history, among others, perioperative low-molecular-weight heparin prophylaxis, lower-limb antithrombotic massage, active lower limb massage, training in respiratory function and other preventive measures are recommended. For other potentially high-risk complications not specified in this study protocol, the doctor in charge of each research participating center can decide on the most appropriate approach according to clinical practice and specific needs of each center and should record it in the CRF.
- For the operative approach of the surgeries in this study is distal gastrectomy and D1+/D2 lymphadenectomy according to the Japanese gastric cancer treatment guidelines 2014 (4th Edition), while reconstruction method should be selected by the doctor in charge according to his/her experience and the specific intraoperative circumstances.
- Preoperative fasting and water deprivation and other before-anesthesia requirements on patients should follow the conventional anesthesia program of each research participating center, which is not specified in this study.
- For prophylactic antibiotics, the first intravenous infusion should begin 30 minutes prior to surgery. It is recommended to select a second-generation

cephalosporin (there are no provisions on specific brands in this study); the preparation, concentration and infusion rate should comply with routine practice; and prophylaxis should not exceed postoperative three days at a frequency of one infusion every 12 hours. If patient is allergic to cephalosporins (including history of allergy or allergy after cephalosporin administration), other types of antibiotics are allowed according to the specific clinical situation and when used over the same time period mentioned.

- Patient data to be collected during the preoperative period also includes CRP
- Informed consent was given to eligible patients 2 days before surgery, and patients were performed randomization.

9.3 Standardization of surgical practice

9.3.1 Handling practices followed by both groups

9.3.1.1 Anesthesia

The operation is to be carried out with endotracheal intubation under general anesthesia; whether epidural assisted anesthesia is applied or not is left at the discretion of the anesthetist and is not specified in this study protocol.

9.3.1.2 Regulations on obtaining sample of the peritoneal lavage

After entering the abdominal cavity, take peritoneal lavage cytology specimens for postoperative examination immediately. More specifically, if ascites is found, sampling the ascites directly. When there is no ascites, 100ml of physiological saline is slowly injected into the abdominal cavity, and then collect samples from Douglas fossa for inspection.

9.3.1.3 Intraoperative exploration

Explore the abdominal cavity for any hepatic, peritoneal, mesenteric, or pelvic metastases and gastric serosal invasion

9.3.1.4 Regulations on the extent of the gastrectomy

Distal gastrectomy was performed on the premise that oncological principles first can be satisfied.

9.3.1.5 Regulations on digestive tract reconstruction

The digestive tract reconstruction method is to be determined by the surgeon

according to his/her own experience and the intraoperative situation. If instrumental anastomosis is used, whether the manual reinforced stitching is to be performed or not on anastomotic stoma is determined by the surgeon and not specified in this study protocol.

9.3.1.6 Regulations on lymph node dissection

Performing D1+/D2 lymphadenectomy according to Japanese gastric cancer guidelines 2014(4th Edition).

9.3.1.7 Regulations on Omentum resection

According to surgeon’s experience and actual needs and are not specified in this study protocol

9.3.1.8 Regulations on surgery-related equipment and instruments

Energy equipment, vascular ligation method, digestive tract cutting closure, and digestive tract reconstruction instruments are determined by the surgeon in charge of the operation according to his/her own experience and actual needs and are not specified in this study protocol.

9.3.1.9 Regulations on gastric canal and peritoneal drainage tube

Whether an indwelling gastric canal or peritoneal drainage tube is left or not after operation is determined by the surgeon in charge of the research participating center according to his/her own experience and actual needs and are not specified in this study protocol.

9.3.1.10 Regulations on simultaneous surgery for other disease

If any other system/organ disease is found during surgery, the responsible surgeon and the consultants of relevant departments should jointly determine performance of a concurrent operation if there is such necessity. The priority of operations is determined according to clinical routine; the patients meeting Exclusion Criteria will be excluded from the PP Set.

9.3.1.11 Regulations on handling of excluded patients as identified intraoperatively

If the surgeon in charge judges and determines that the patient undergoing surgery belongs to the exclusion case group, then the research approach is suspended and the surgeon will follow routine clinical practice of the research participating center

to decide subsequent treatment (therapeutic decisions as to whether to excise gastric primary focus and metastases are made by the surgeon in charge); The excluded cases still need to complete data collection and follow-up and included in the analysis study (ITTP population).

9.3.1.12Regulations on imagery/photographing

A digital camera (8 million pixels at least) will be used to take pictures which shall contain the following contents (see the example below):

(2) Field of lymph node dissection (5 pictures)

Inferior pylorus region (1 picture); the right gastroepiploic arteriovenous cut site should be included.

Right-side area of the superior margin of the pancreas (1 picture); the front top of the entire common hepatic artery, the half front of the inferior proper hepatic artery and the cut site of the right gastric artery should be included.

Left-side region of the superior margin of the pancreas (1 picture); the left gastric arteriovenous cut position, celiac arterial trunk and proximal splenic artery should be included.

Right side of the cardia and lesser gastric curvature side (1 picture).

Left gastroepiploic vessel dividing position (1 picture); the cut site of the left gastroepiploic artery and vein should be included.

(2) After the skin incision is closed (1 picture, measuring scale serving as a reference object).

(3) Postoperative fresh specimens (4 pictures, measuring scale serving as a reference object); 1 picture before and 3 pictures after dissection (mark focus size; 1 picture each of distal and proximal incisional margins). After the specimen is cut open along the greater gastric curvature, a measuring scale is placed as a reference object before taking pictures to record the following items: the distance between the tumor edge and the proximal incisional margin (1 picture), the distance between the tumor edge and the distal incisional margin (1 picture), and the focus size and appearance of the mucosal face after the specimen is unfolded (1 picture).

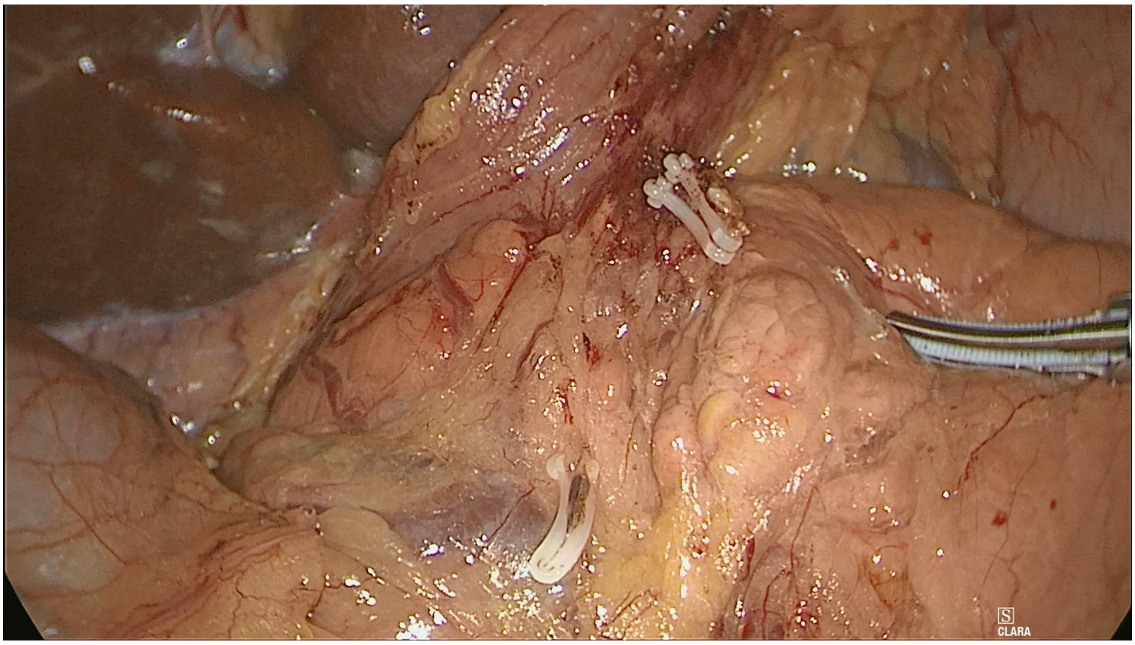

Fig. 2-1A Inferior pylorus area for laparoscopic surgery (no. 6 lymph nodes)

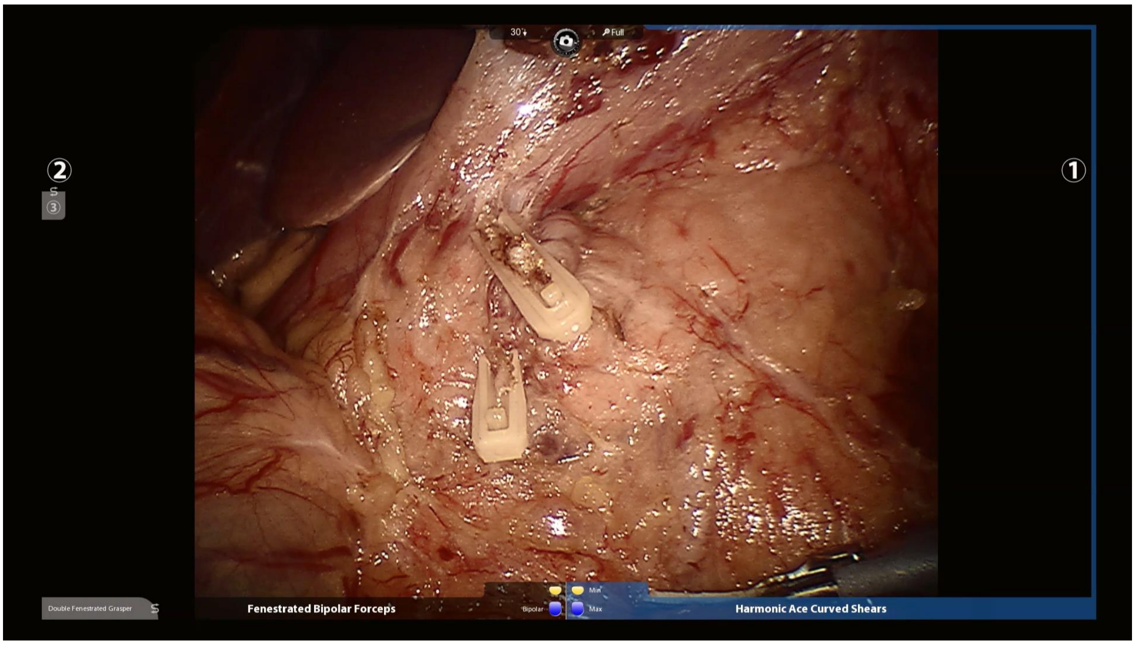

Fig. 2-1B Inferior pylorus area for robotic surgery (no. 6 lymph nodes)

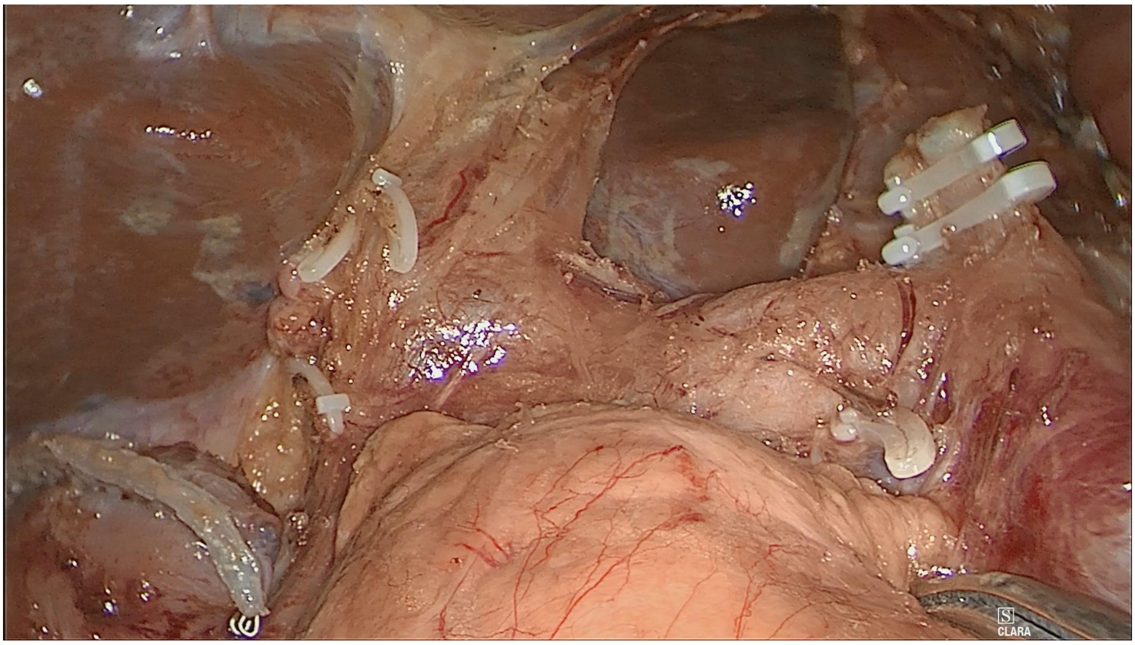

Fig. 2-2A Right-side area of the superior margin of the pancreas for laparoscopic surgery (no. 5, no. 8a and no. 12a lymph nodes)

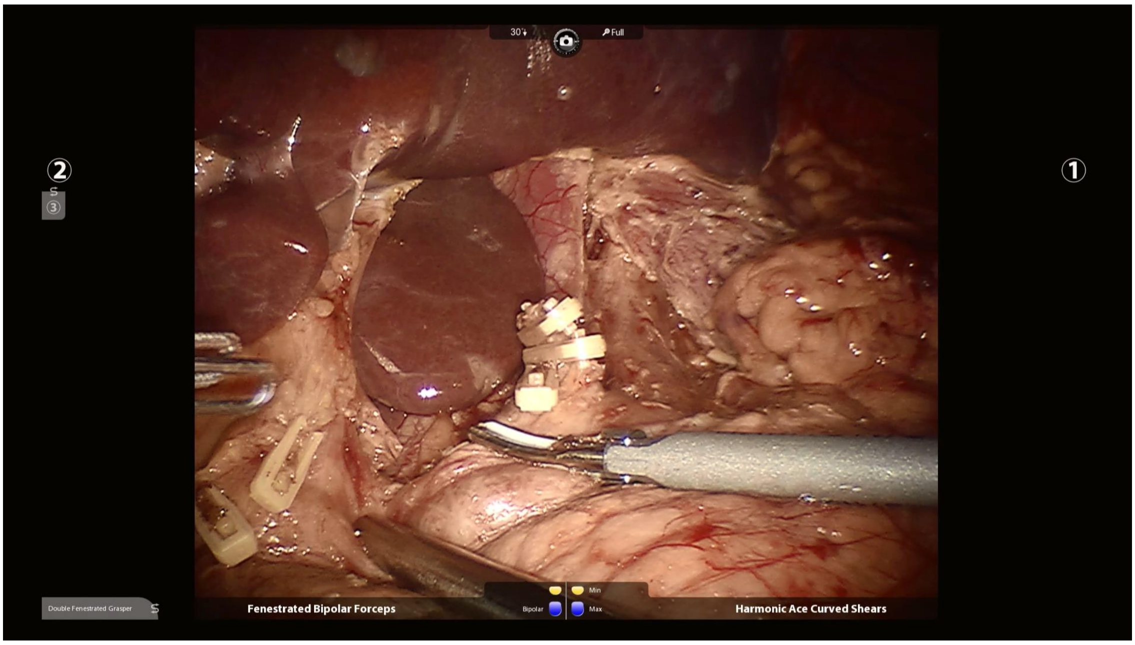

Fig. 2-2B Right-side area of the superior margin of the pancreas for robotic surgery (no. 5, no. 8a and no. 12a lymph nodes)

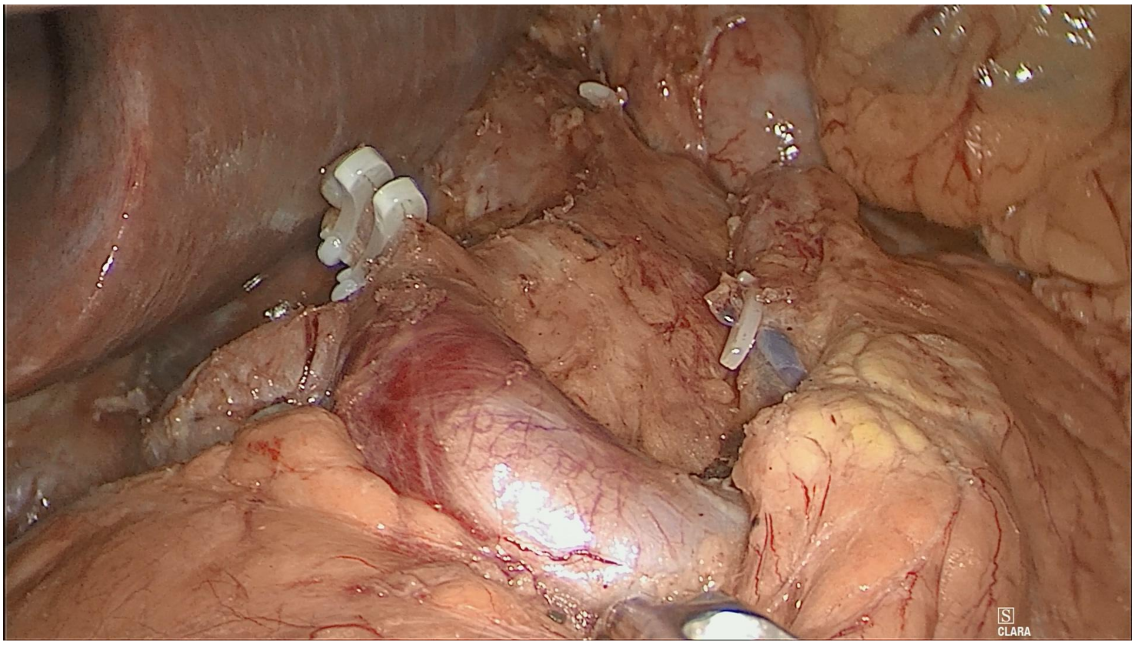

Fig. 2-3A Left-side area of the superior margin of the pancreas for laparoscopic surgery (no. 7, no. 9 and no. 11p lymph nodes)

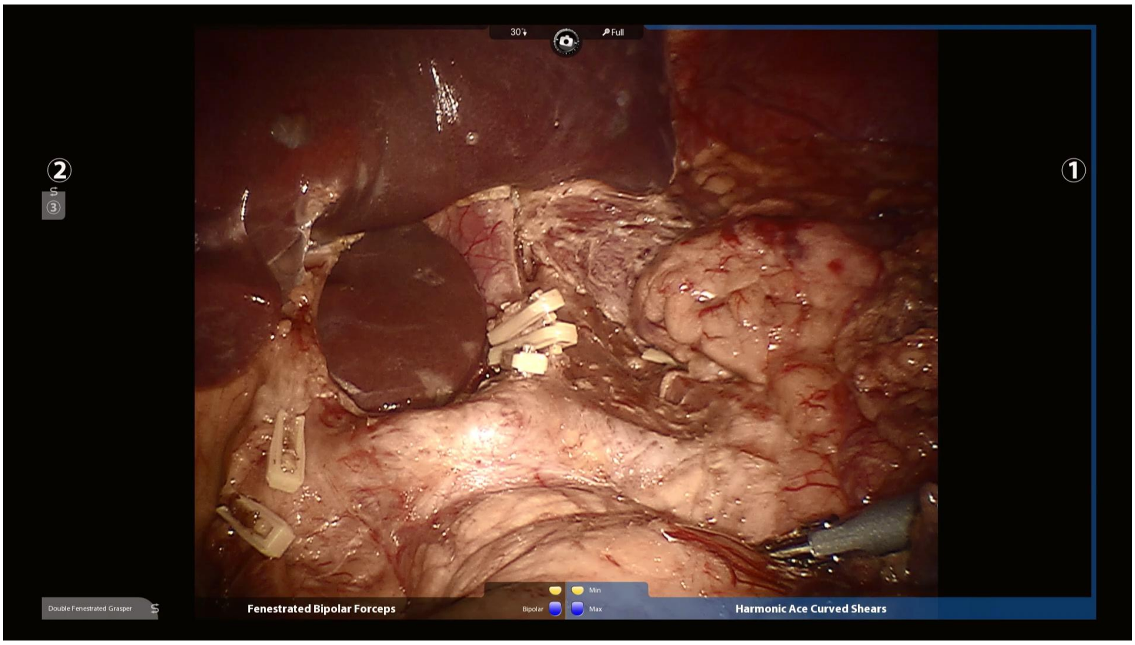

Fig. 2-3B Left-side area of the superior margin of the pancreas for robotic surgery (no. 7, no. 9 and no. 11p lymph nodes)

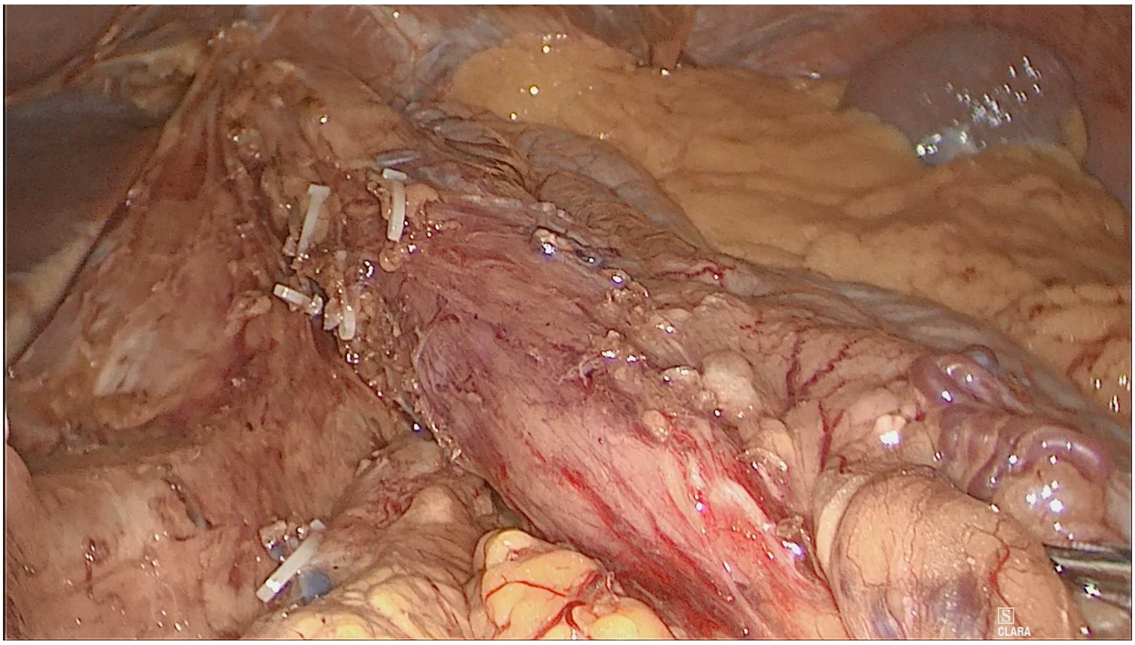

Fig. 2-4A Right side of the cardia and lesser gastric curvature side for laparoscopic surgery (the no. 1 and no. 3 lymph nodes)

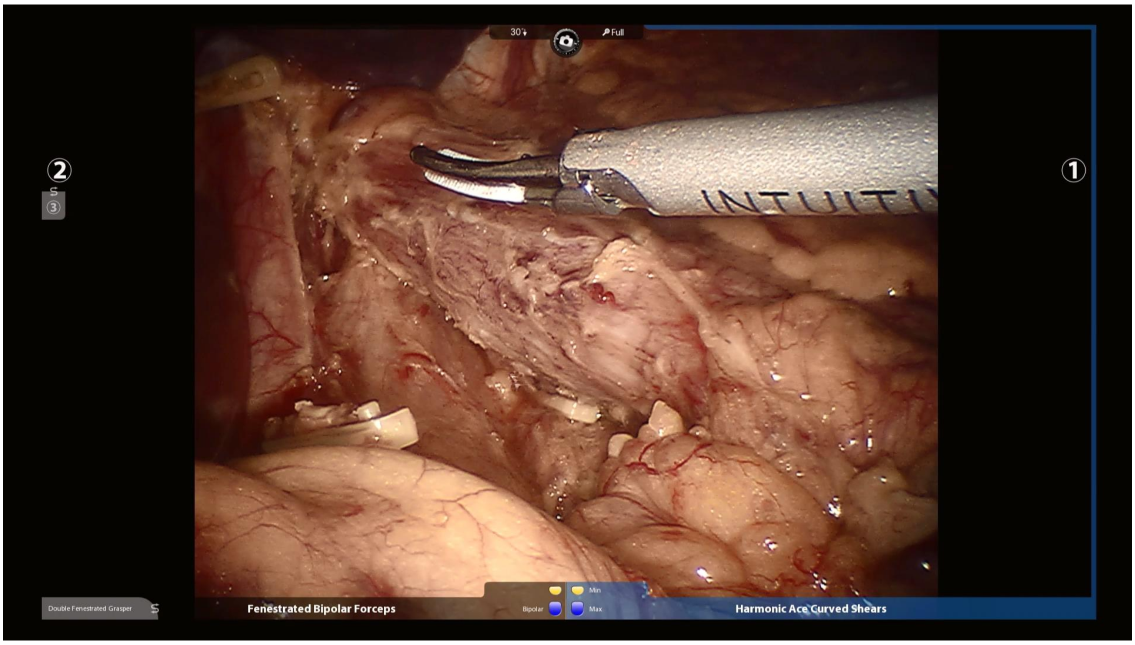

Fig. 2-4B Right side of the cardia and lesser gastric curvature side for robotic surgery (the no. 1 and no. 3 lymph nodes)

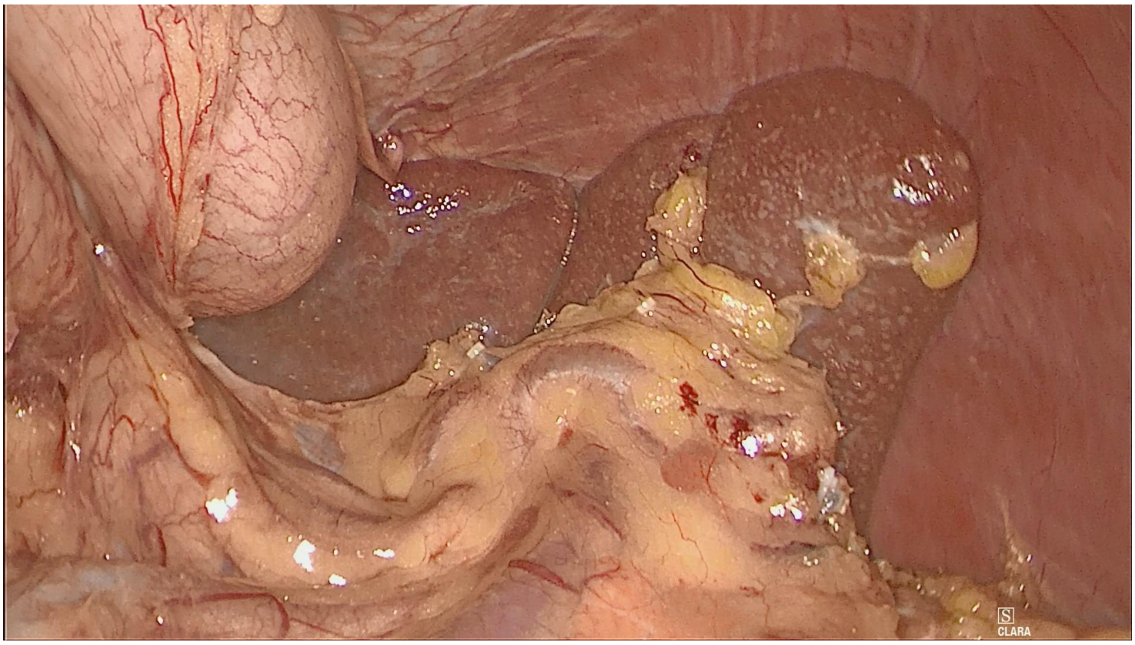

Fig. 2-5A Cut site of the left gastroepiploic vessel for laparoscopic surgery (no. 4 sb lymph nodes)

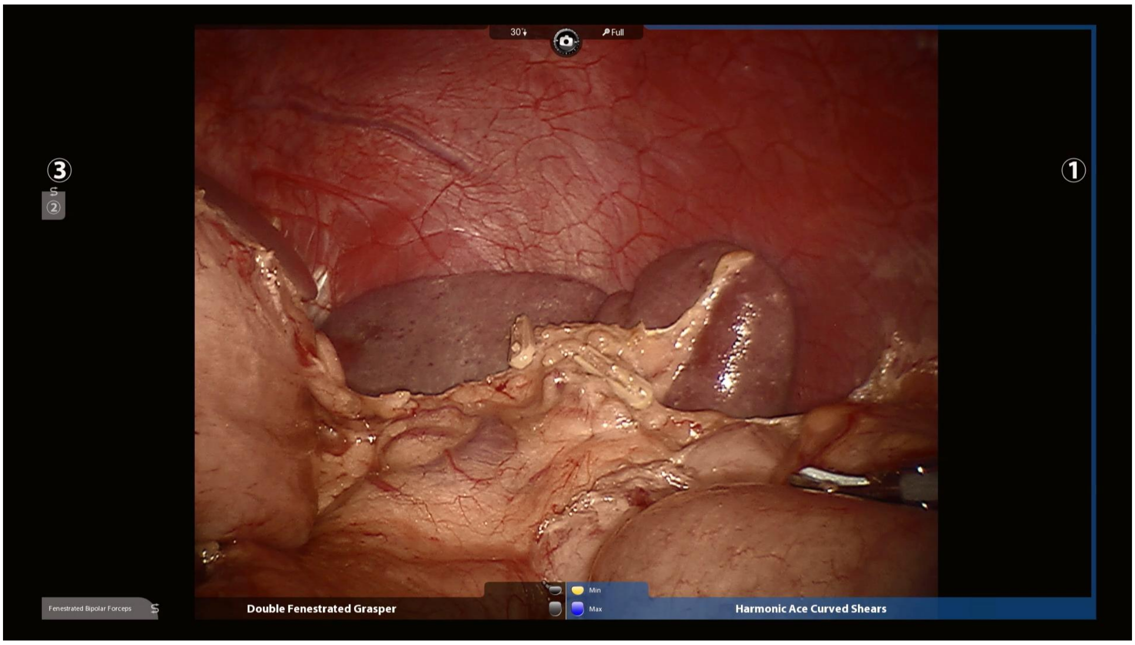

Fig. 2-5B Cut site of the left gastroepiploic vessel for laparoscopic surgery (no. 4 sb lymph nodes)

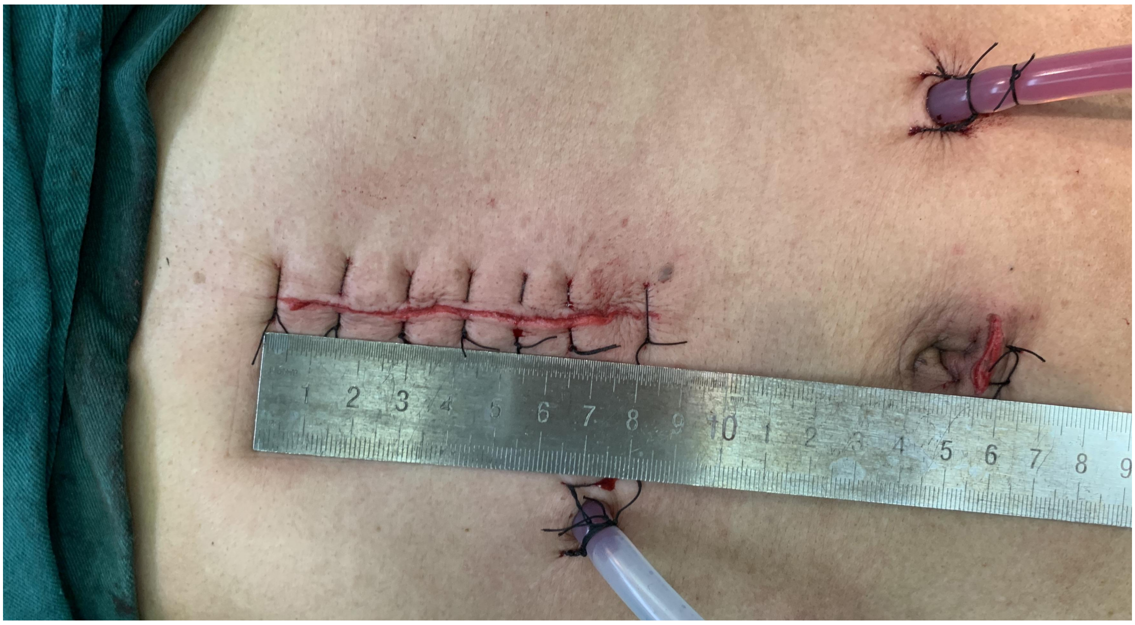

Fig. 2-6 Incision appearance (mark the incision length)

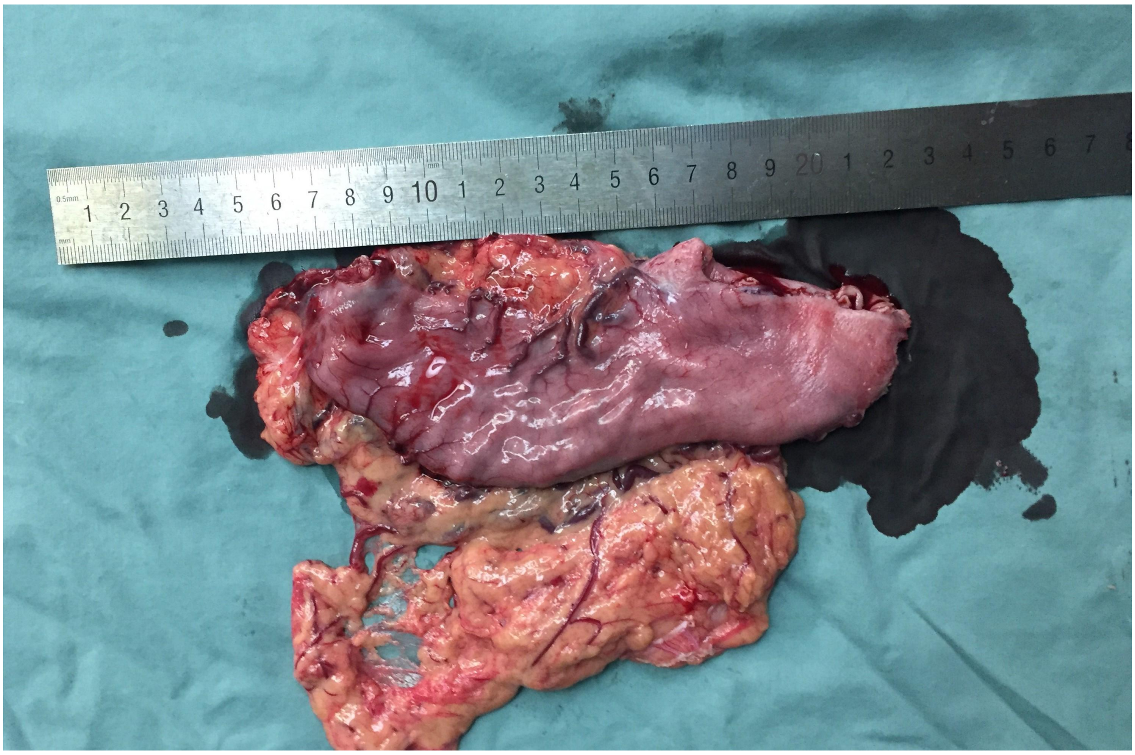

Fig. 2-7 Specimen observation (before dissection)

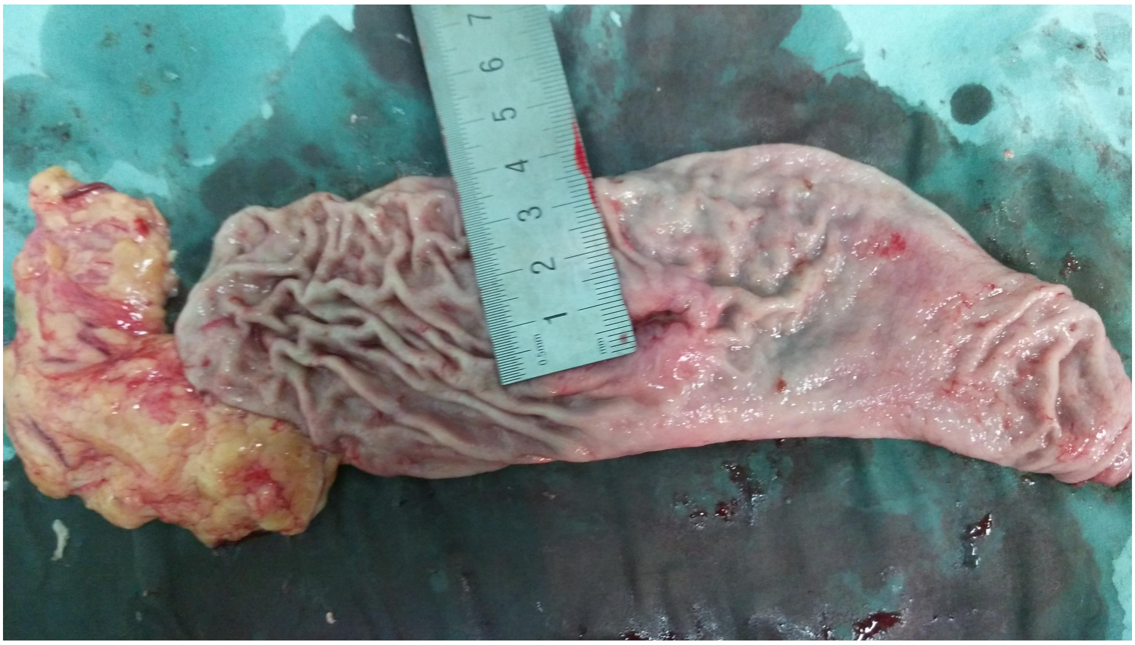

Fig. 2-8 Specimen observation (focus size; the dissection is made along the greater gastric curvature, and the focus and incisional margin on the mucosal face are observed; if the tumor is located at the greater gastric curvature, then the dissection is made along the lesser curvature)

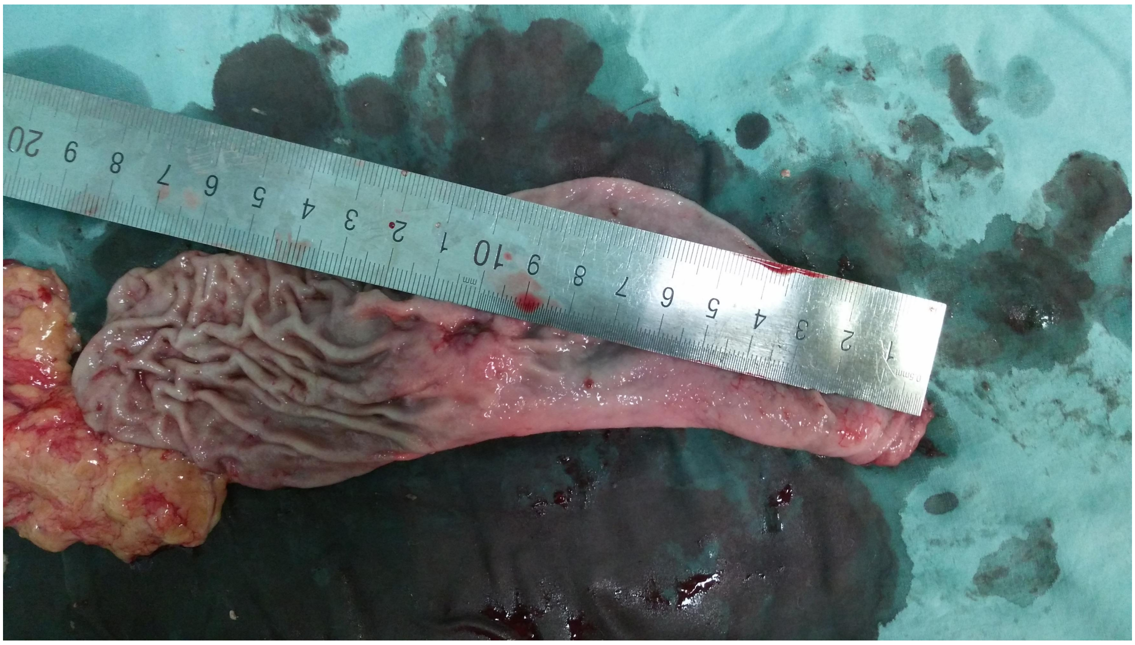

Fig. 2-9 Specimen observation (the distance between the tumor edge and the proximal incisional margin)

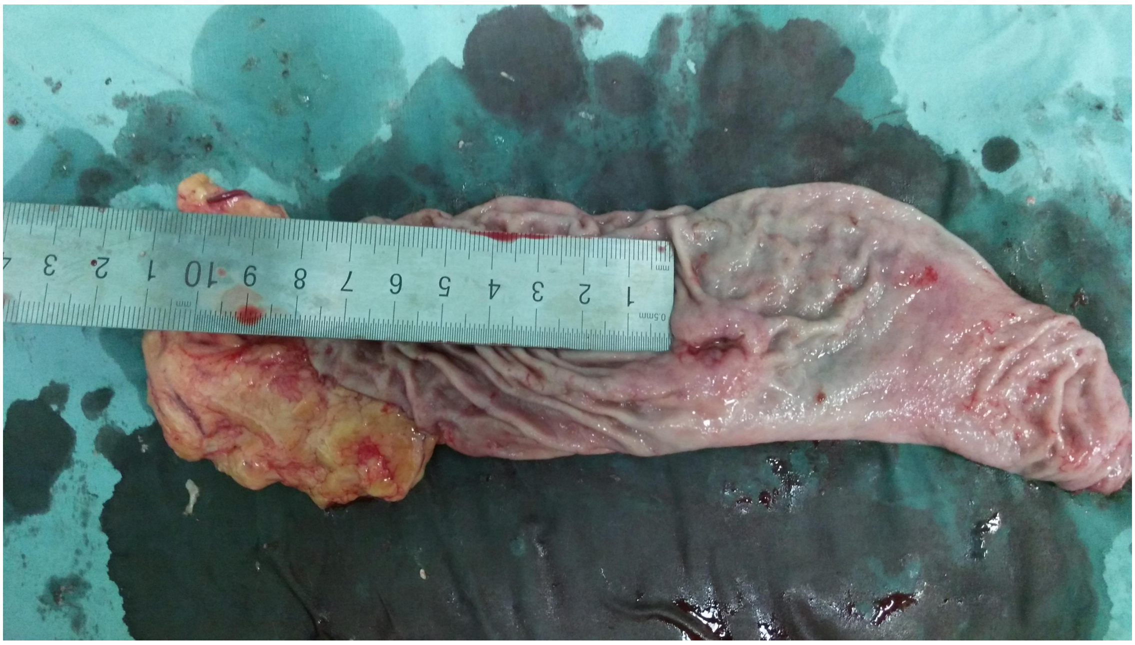

Fig. 2-10 Specimen observation (the distance between the tumor edge and the distal incisional margin)

9.3.1.13 Regulations on the photo/ image privacy protection and naming

No image data shall disclose the personal information of patients.

When the photos/images are viewed or reviewed, the personal information must be processed with mosaics or be covered.

The photographed parts should be marked with unified Chinese name: inferior pylorus area; left gastroepiploic vessel cut site; right-side area of superior margin of the pancreas; left-side area of superior margin of the pancreas; right side of the cardia and lesser gastric curvature side; incision appearance; specimen observation (before dissection); specimen observation (focus size); specimen observation (the distance between the tumor edge and the proximal incisional margin); and specimen observation (the distance between the tumor edge and the distal incisional margin).

For example:

Photo Name: [Robot-subject's random number - Inferior pylorus area]/ [Lap-ICG-subject's random number - Inferior pylorus area]

Folder name: [Robot-subject's random number]/ [LAP-ICG-subject's random number]

9.3.1.14 Criteria for confirming operation quality

To confirm the appropriateness of the surgical procedure, surgery quality, (auxiliary) incision length and specimen integrity will be assessed in the photographs saved (as stated above) The whole laparoscopic surgery procedure will be videotaped, and the unclipped image files will be saved.

9.3.1.15 Saving of imaging data

All photographs and data will be saved in the hard disk or portable digital carrier in digital form, and the surgical video required a specific hard drive to be saved for at least 3 years.

If failure to provide the complete photo according to "Regulations on imagery/photographing" is confirmed, the Research Committee will judge and record the surgery quality as unqualified; however, the case will remain in the PP set data of this study.

9.3.2Regulations on laparoscopic/robotic surgery

9.3.2.1 Regulations on pneumoperitoneum

Carbon dioxide pneumoperitoneum will be used to maintain the pressure at 12-13 mmHg.

9.3.2.2 Regulations on punctures and auxiliary incision

The positions of punctures and auxiliary small incision are not specified; the number of punctures should not exceed 5. There should be only one auxiliary small incision whose length shall not exceed the maximum tumor diameter and necessarily will be less than 10 cm in normal cases. If the auxiliary small incision needs to be longer than 10 cm, the surgeon in charge should make a decision and record the reasons in the CRF.

9.3.2.3 Definition of laparoscopic/robotic approach

The operations within the abdominal cavity must be performed using laparoscopic/robotic instruments with the support of a camera/Da Vinci system. Perigastric disassociation, greater omentum excision, omental bursa excision, lymph node dissection, and blood vessel handling are completed under laparoscopic/robotic guidance. For gastrectomy and digestive tract reconstruction use of auxiliary small incisions is allowed and can be completed with an opened abdomen.

9.3.2.4 Regulations on conversion to laparotomy

When intra-abdominal hemorrhage, organ damage and other serious/life-threatening complications which are difficult to control occur during laparoscopic surgery, it is necessary to actively convert to laparotomy. If the anesthesiologist and surgeon consider that intraoperative complications caused by carbon dioxide pneumoperitoneum may threaten the patient’s life, it is necessary to actively convert to open. The surgeon in charge can decide to convert to laparotomy driven by other technical or equipment reasons and will record said reasons. The reasons for the conversion to open must be clearly recorded in the CRF. The incision length of > 10 cm is defined as a case of conversion to open surgery in this study.

9.3.2.5 Subsequent treatment of excluded patients from the laparoscopic group

Whether the patients continue to undergo surgery under laparoscopy or converted to open surgery is at surgeon’s discretion according to clinical experience.

9.3.3 Operative parameters (same for both groups)

Completed by the research assistant on the day of the operation. specific projects include:

(1) Name of responsible surgeons

(2) Operation time (min)

(3) Type of operation, digestive tract reconstruction, intraoperative damage and whether the tumor was ruptured during surgery (intact rupture of the capsule)

(4) Length of incision (cm)

(5) Conversion to open surgery or not and the reasons for this decision

(6) Intraoperative estimated blood loss (ml; from skin cutting to stitching, intraoperative blood loss = (postoperative gauze weight, grams - preoperative gauze weight, grams) *1ml/g+ suction fluid, ml)

(7) Blood transfusion (ml): in this study, the blood transfusion event is defined as transfusion of red cell suspension (ml) or whole blood (ml)

(8) Tumor location

(9) Tumor size (maximum tumor diameter, mm)

(10) Distant metastasis (location)

(11) Proximal resected margin (mm), distal resected margin (mm), radicality（R0/R1/R2）

(12) Intraoperative complications (occurring from skin incision to skin closure)

including:

surgery-related complications: intraoperative hemorrhage and injury: A. Vascular injury: A vascular injury is defined as a blood vessel with either a blood vessel clamp or a titanium clamp closure and an intra-cavity suture or any other method to control the bleeding. B. Organ damage: maybe including diaphragmatic injury, esophageal injury, duodenal injury, colon injury, small intestine injury, spleen injury (excluding <1/3 spleen ischemia), liver injury, pancreatic injury, gallbladder injury, kidney damage etc.C. Tumor rupture: tumor envelope Integrity damage air abdominal-related complications: high-blood carbonate, mediastinal emphysema, subcutaneous emphysema, air embolism, respiratory circulation instability caused by abdominal pressure.

Anesthesia-related complications: Allergic reactions.

(14) Intraoperative death (occurring during the time period from skin cutting to skin stitching completion) regardless of reason.

9.4 Postoperative management (same for both groups**)**

9.4.1 The use of prophylactic analgesics

Continuous postoperative prophylactic intravenous analgesia is allowable but not mandatory within postoperative 48 hours; its dose, type and rate of infusion should be determined by the anesthesiologist according to clinical practices and specific patient conditions. The repeated use of prophylactic analgesics is not allowed beyond 48 hours after the end of surgery, unless it is judged necessary

9.4.2 Fluid replacement and nutritional support

Postoperative fluid infusion (including glucose, insulin, electrolytes, vitamins, etc.) or nutritional support (enteral/parenteral) will be performed based on doctor's experience and routine clinical practices and is not specified in this study. After oral feeding, it is allowable to stop or gradually reduce fluid infusion/nutritional support.

9.4.3 Post-operative rehabilitation management

Management methods of incision, stomach and abdominal drainage tube: Follow regular diagnosis and treatment approaches. Eating recovery time, diet transition strategies: Follow regular diagnosis and treatment approaches.

9.4.4 Discharge standard

Patients needed to meet the following criteria for discharge: 1) satisfactory intake

of a soft diet. 2) move around of their bed. and 3) absence of complications by routine clinical examinations. This information will be recorded in the CRF.

9.4.5 Postoperative observation items

Definition of “postoperative day n”: One day from 0:00 to up to 24:00. Up to 24:00 on the day of surgery is “postoperative day 0;” the next day from 0:00 to up to 24:00 is “postoperative day 1;” and so on. From the first postoperative day until hospital discharge, the research assistant should timely fill in the following items and specific observation items including:

(1) Pathologic results：

Original lesion tissue typing, Distant metastasis, and parts, NIH Hazard grading, Radical surgery degree (R0/R1/R2)

(2) Postoperative complications：

Postoperative complications are divided into and short-term complications after surgery and long-term complications after surgery. Short-term is defined as within 30 days of surgery or the first discharge if the hospital days > 30 days. Long-term is defined as the period from 30 days or more after the operation, or the first discharge (the hospital days after surgery >30 days) to 3 years after the operation.

Classification and name of complication	Diagnostic criteria
Abdominal bleeding	Intra-abdominal hemorrhage requires blood transfusion, emergency endoscopy or surgical intervention to eliminate anastomotic bleeding
Anastomotic bleeding	The postoperative gastrointestinal decompression tube continued to have fresh red blood outflow; the hemoglobin drops more than 1g/dL
Gastrointestinal anastomotic stoma Fistula	Using gastrointestinal angiography to see contrast agent leak out from the anastomosis, or the blue drainage outflow through tube after oral Methylene blue to eliminate the possibility duodenal stump fistula and intestinal fistula
Duodenal Stump Fistula	Using gastrointestinal angiography to see contrast agent leak out from the duodenal stump to eliminate the anastomotic fistula or intestinal fistula
Intestinal fistula	Using gastrointestinal angiography to see the blue drainage outflow

	through tube after oral Methylene blue to eliminate anastomotic fistula and duodenal stump fistula
Stenosis of Anastomosis	Endoscopic examination with a 9.2-mm endoscopy not passing through the anastomosis to eliminate recurrence of tumors
Input jejunal loop obstruction	Abdominal pain, abdominal distension, vomiting and other symptoms. Abdominal flat to see the right upper abdomen expansion of the intestinal loop, and there is a liquid plane, or a visible input loop jejunum giant expansion by barium meal examination.
Intestinal obstruction after operation	Abdominal X-ray shows a plurality of liquid planes and the phenomenon of intestinal effusion with visible isolated, fixed, swelling of the intestinal loop. Total Abdominal CT showed edema, thickening, adhesion of intestinal wall, accumulation of gas in intestinal cavity, uniform expansion of bowel and intra-abdominal exudation.
Early dumping syndrome	Combined the symptoms of sweating, heat, weakness, dizziness, palpitations, heart swelling feeling, vomiting, abdominal colic or diarrhea with the signs of tachycardia, blood pressure micro-rise, breathing a little faster sign after meal 15-30 minutes, and solid phase radionuclide gastric emptying scanning tips stomach quickly emptying.
Late dumping syndrome	Feeling hungry, flustered, out of sweating 2-3 hours after the meal . Blood sugar is less than 2.9mmol/L, excluding other diseases that cause hypoglycemia
Intestinal ischemia and necrosis	Under the digestive endoscopy, the intestinal mucosa congestion, edema, bruising, mucosal hemorrhage, the mucous membrane being dark red, the vascular network disappearing, can have part mucosal necrosis, following with mucosal shedding, ulcer formation with annular, longitudinal, snake and scattered in the ulcer erosion.
Internal hernia	Postoperative CT findings of cystic or cystic and solid mass, and intestinal aggregation, stretching, translocation, abnormal mesenteric movement, and thickening of the blood vessel.
Alkaline reflux esophagitis	1. Endoscopic examination and biopsy of the upper gastrointestinal tract showed evidence of inflammation of the mucous membranes and gastrointestinal metaplasia; 2. CT scan and gastrointestinal barium meal examination showed no expansion or obstruction of the input loop.

Incision splitting	Including partial dehiscence of the incision and full-layer dehiscence
Incisional hernia of abdominal wall	The swelling tumor showing in the surgical scar area or abdominal wall swelling when standing or force. CT shows ventral wall continuity interruption and hernia content extravasation
Incision infection	Thickening of the soft tissue at the incision, in or below the incision of gas, exudation, swelling of the incision or pus from the incision extrusion, or secretion culture of pathogenic bacteria.
Lymphatic leakage	A chyle test when abdominal drainage fluid exceeded 300 ml/day for 5 consecutive days after postoperative day 3.
Pneumonia	Complies with one of the following two diagnostic Criteria: 1. Auscultation/percussion voiced + one of the following: fresh sputum or sputum character changes; blood culture (+); bronchoalveolar lavage fluid, anti-pollution sample brush, biopsy specimens cultured pathogenic bacteria. 2. Chest film hints of new or progressive infiltration + one of the following: fresh sputum or sputum character changes, blood culture (+), bronchoalveolar lavage fluid, anti-pollution sample brush, biopsy specimens cultured pathogenic bacteria; isolate virus or detect IgM, IgG (+) of respiratory viral
Acute pancreatitis	Irritability, abdominal pain, anti-jumping pain, fever, leukocyte increase and blood amylase increased occuring and diagnosed by ultrasound or CT within 3 days after surgery.
Acute cholecystitis	Serum bilirubin exceeding 85μmol/l and ultrsound examination shows gallbladder enlargement, wall thickness, signal and sound shadow of gallbladder stone, bile internal sediment, gallbladder contraction bad etc.
Pleural effusion/infection	CT scan showed the localized fluid low density area of thoracic cavity, which could accompany with gas, and culture pathogenic bacteria in thoracic endocrine.
Abdominal infection	There is at least one of the following evidences in abdominal cavity within 30 days after operation: 1. discharge of pus, with/without microbiological examination; 2. bacterial culture positive; 3. diagnosed by detection, pathology, imaging findings.
Pelvic infection	Symptoms of systemic infection or rectal irritation, combined with a rectal finger examination and touching tenderness, or a married

	woman with a posterior vault to extract pus-based fluid
Sepsis	The following two conditions are available: 1. There is evidence of active bacterial infection, but the blood culture does not necessarily appear pathogenic bacteria; 2. meeting two of the following four items at the same time: (1). body temperature >39. 0℃ or < 35.5 ℃ for 3 consecutive days, (2). heart rate > 120 times/min; (3). total white blood cells >12. $0*10^9$/L or <$4.0*10^9$/l, wherein neutrophils >0. 80, or naïve granular cells >0. 10; (4).Respiratory frequency > 28 times/min
Urinary system infection	Symptoms of urine frequency, urgency and urine pain etc. and urine bacteria culture colony count 1000~10 million/ml in the absence of antibiotics; No symptoms of urine frequency, urgency and urine pain etc, urine bacterial culture colony count ≥ 100,000/ml
Pancreatic fistula	The level of amylase in the drainage fluid is three times than normal level.
Bile fistula	Symptoms of abdominal distension, Abdominal pain, tenderness, anti-jumping pain, muscle tension, abdominal puncture or drainage fluid for bile
Celiac fistula	The drainage fluid is milky white, and more than 200ml/d and and does not decrease for 48 hour, the celiac qualitative test is positive, and the level of triglyceride >110 mg/dL at the same time.
Nutritional disorder after gastrectomy	In the presence of weight loss, anemia, malnutrition bone disease, vitamin A deficiency and other symptoms, laboratory tests suggest that the intestinal absorption function test is abnormal, excluding other causes of nutritional disorders
Bone disease after gastrectomy	Lumbar back pain, length shortening, kyphosis, bone fractures and other symptoms. Bone density decreased combining with elevated alkaline phosphatase and serum calcium reduction, the concentration of serum 25-(O1) D3 and 1,25-(O1) 2D3 increasing and the serum parathyroid hormone increasing. Exclusion of bone disease caused by other causes.
Subcutaneous emphysema	visible the irregular speckle shadow under the skin in the horizontal flat sheet.
Mediastinal emphysema	In the posterior and anterior flat fame, a long narrow gas shadow rises

	to the neck soft tissue along the mediastinal side, forming a thin-line dense shadow. In the lateral flat there was a visible and clear band between the heart and the sternum. The CT examination, if necessary, shows gas density line-like shadow around the mediastinal and mediastinal pleura closing to the direction of the lung field.
Postoperative hemorrhage	An amount of hemorrhage exceeding 300 ml.
Postoperative cardiac dysfunction	The symptom of snus tachycardia, sinus bradycardia, supraventricular tachycardia, ventricular tachycardia, and other arrhythmias, or heart failure preoperatively none-existing and postoperatively appearing,.and other causes of the above-mentioned manifestations are excluded.
Hepatic dysfunction	Bilirubin increasing and the levels of AST and ALT >5 times after operation and these symptoms no existing before sugery,
Kidney function failure	Postoperative continuing renal function insufficiency, blood creatinine rising 2mg/dl, or acute renal failure needing dialysis treatment.
Cerebral embolism	Acute onset, hemiplegia, aphasia and other focal neurological function deficits. Embolism site has low-density infarction, of which border is not clear and no obstructional performance within 24-48 hours after the onset.
Pulmonary embolism	Characteristics of dyspnea, chest pain, syncope, shortness of breath, right ventricular insufficiency and hypotension, pulmonary angiography revealed a filling defect.
Venous thrombosis of lower extremities	Local tenderness, swelling, purple skin color, combined with intravenous angiography to show the filling defect
Mesenteric arterial embolization	Patients with acute abdominal pain, vomiting, diarrhea, abdominal x-ray of intestinal tract filling with gas or existing liquid level, abdominal angiography revealed a filling defect.
DIC	1.There are basic diseases easily leading to DIC, 2. There are more than two clinical performances: (1) severe or multiple bleeding tendencies; (2) Microcirculation disorder or shock cannot be explained by the original disease. (3) Extensive skin mucosal embolism, focal ischemic necrosis, shedding and ulcer formation, or unexplained lung, kidney, brain and another organ failure. (4) anticoagulant treatment.is effective. 3. The laboratory meets the following

	conditions: (1) there are 3 or more experimental abnormalities: platelet count, prothrombin time, activated partial coagulation enzyme time, thrombin time, fibrinogen level, D-two poly, and (2) difficult or special cases for special examination.
Other	Complications other than the above complications, which do not exist before surgery but appear after surgery

Severity of complication is graded according to Clavien–dindo complication scoring system, [31] IIIA level and above are serious complication

Ⅰ: Any deviation from the normal postoperative course without the need for pharmacologic treatment or surgical, endoscopic, and radiologic interventions. Allowed therapeutic regimens are drugs as antiemetics, antipyretics, analgesics, and diuretics, and electrolytes and physiotherapy. This grade also includes wound infections opened at the bedside.

Ⅱ： Requiring pharmacologic treatment with drugs other than such allowed for

grade I complications. Blood transfusions and total parenteral nutrition are also included.

Ⅲ： Requiring surgical, endoscopic, or radiologic intervention

Ⅲa： Intervention not under general anesthesia

Ⅲb： Intervention under general anesthesia

Ⅳ： Life-threatening complication (including CNS complications) requiring IC(intermediate care)/ICU(intensive care unit)
management

Ⅳa： Single organ dysfunction (including dialysis)

Ⅳb： Multiple organ dysfunction

Ⅴ： Death as a result of complications

(3) Blood test items (At postoperative day 1, 5)

Peripheral blood routine assessment: Hb, RBC, WBC, LYM, NEU, NEU%, and PLT、MONO；

Blood biochemistry: Albumin, prealbumin, total bilirubin, AST, ALT, creatinine, urea nitrogen, fasting blood glucose, potassium, sodium, chlorine, calcium and CRP.

(5) Postoperative rehabilitation evaluation：

Time to first ambulation (hours), time to first flatus (hour), time to liquid diet, time to semi-liquid diet (hour), daily body temperature maximum from surgery to out-patient (℃), time to removal of gastric tube (d), daily volume of gastric drainage (ml), time to removal of abdominal drainage tube (d), daily volume of drainage (ml).

Blood transfusion volume (ml) from the end of surgery to postoperative discharge: a transfusion event is defined as infusion of the red blood cell suspension (ml) or whole blood (ml)

Postoperative hospital stay (days): periods form surgery day to first discharge day

9.5 Follow-Up

9.5.1 Follow-up Period and strategy

Follow-up visits will be completed by special persons for all cases selected in this study .All patients are followed up with every 3 months during the first 2 years and then every 6 months beyond the third year (1、 3、 6、 9、 12、 15、18、21、24、 30 and 36 months after the operation). This study suggests that the above examinations should be conducted in the patient's primary surgical research center, but does not exclude outer court review. For Outer Court review, It recommended that visiting the hospital as a three-level hospital, and these information will be recorded by the follow-up specialist. The occurrence of tumor recurrence or metastasis and the survival status of all patients are evaluated and recorded according to the results of the various examinations. Patients who refuse to follow the protocol should be recorded as lost to follow-up, and at the end of the study, these cases should be analyzed together with cases lost to follow-up in line with the criteria of this study.

9.5.2 Assessment items during the follow-up

(1) Systematic physical examination:

The doctor in charge will regularly conduct a systematic physical examination at the time of each follow-up, giving particular attention to superficial lymph nodes, abdomen, and signs of metastases, among others.

(2) Blood test items:

Peripheral blood routine assessment: Hb、RBC、WBC、LYM、NEU、NEU%、PLT、MONO

Biochemistry: Albumin, pre-albumin, total bilirubin, Indirect bilirubin, direct

bilirubin, AST, ALT, creatinine, urea nitrogen, Total cholesterol, triglycerides, fasting blood glucose, potassium, sodium, chlorine, calcium, serum tumor markers: CEA、CA19-9、CA72-4、CA12-5、AFP

(3) Imaging items:

Whole abdomen (including cavity) CT (thickness of 10 mm or less, in case of contrast agent allergy, CT horizontal scanning is only allowable or conversion to MRI). Upper gastrointestinal endoscopy (histopathological biopsy, endoscopic ultrasonography when necessary). Chest X-ray (AP and lateral views): lung field condition. Other means of evaluation: gastrointestinal radiography, ultrasonography of other organs, whole body bone scanning, and PET-CT, among others used at physician's discretion.

9.5.3 Follow-up process

Postoperative	3 months	6 months	9 months	12 months	15 months	18 months	21 months	2 years	2 years and a half	3 years
Date of actual visit										
Physical examination										
Blood Routine										
Blood biochemistry										
Tumor Markers										
Chest slices										
Upper digestive tract endoscopy	/	/	/		/	/	/		/	
Abdominal CT	/		/		/		/			
Full abdominal ultrasound		/		/		/		/	/	/

Other (if necessary)										

9.5.4 Other items on follow-up process

- Requirement for the retention follow-up call was recommended, to contact patients for consultation information
- Telephone follow-up procedures were added to the protocol for visits unable to be conducted due to COVID-19. Missed clinic visits were to be reported as such and considered protocol deviations.

9.6 Post-operative adjuvant therapy

9.6.1 Indications for postoperative adjuvant chemotherapy

After completion of the surgical treatment, according to the postoperative pathological results, subjects among the R0 resection cases that are stage II and above are administered postoperative adjuvant chemotherapy according to the provisions of this program.

For cases of non-R0 resection or recurrence after R0 resection, this study does not stipulate the follow-up treatment plan; each research center decides on the action to be taken according to the clinical treatment routine.

9.6.2 Postoperative adjuvant chemotherapy

The chemotherapy treating oncologists were unaware of the intervention received by the patients.

This study uses a combination of chemotherapy based on 5-FU (5-fluorouracil) with platinum or docetaxel.

The adjuvant chemotherapy cycle is half a year (6 months postoperatively).

In cases of good physical and tolerable conditions, chemotherapy is first started within 8 weeks after surgery and then according to the regularity of the chemotherapy cycle.

During the chemotherapy period, tumor recurrence should be assessed according to the follow-up plan.

When tumor recurrence occurs during chemotherapy, the adjuvant

chemotherapy regimen of this study is discontinued. The follow-up treatment is decided by each research center according to the clinical treatment routine. This study does not make regulations, but the cause and follow-up treatment plan should be recorded in the CRF.

If there is no recurrence during chemotherapy, adjuvant chemotherapy is terminated after 6 months, and the follow-up plan continues.

Adjuvant chemotherapy requires written approval from the patient.

Subjects that refuse postoperative adjuvant chemotherapy or do not complete the adjuvant chemotherapy are not excluded from this study, but the cause is marked and recorded in the CRF.

For elderly patients (70 years and older), considering differences in the physical fitness of the elderly and ensuring the safety of patients, each research center decides according to the clinical treatment routine. This study does not recommend or stipulate any chemotherapy regimen for patients of this age.

Patients who choose adjuvant chemotherapy, irregular chemotherapy, or a nonfirst-line regimen are not excluded from the study, but the Efficacy and Safety Evaluation Committee is obliged to monitor patient safety during follow-up. The patient's chemotherapy medication must be recorded in the CRF.

The principles of processing in terms of the method of administration of adjuvant chemotherapy, toxic reactions, and dose adjustment with intolerance are implemented according to the original literature on drug toxicity and dose adjustment for each chemotherapy regimen. This study does not regulate these principles.

9.6.3 Safety Evaluation Indicators of Postoperative Adjuvant Chemotherapy

The safety evaluation indicators for patients enrolled in the study should be immediately filled out by the investigators before and after each postoperative adjuvant chemotherapy cycle, with specific items including the following:

(1) Performance Status (ECOG)

(2) Subjective and objective status (according to the records of CTCAE v3.0 Short Name)

(3) Blood tests:

Peripheral venous blood assessment: Hb, RBC, WBC, LYM, NEU, NEU%, PLT, MONO.

Blood biochemistry: albumin, prealbumin, total bilirubin, AST, ALT, creatinine, urea nitrogen, fasting blood glucose, serum tumor markers (CEA, CA19-9, CA72-4, CA12-5, AFP)

(4) Safety evaluation items to be implemented during chemotherapy when necessary (refer to CTCAE v3.0):

Neurotoxicity

Cardiovascular system (cardiac toxicity, ischemic heart disease, etc.)

Bone marrow suppression and infections due to immune dysfunction

Others

9.7 Study calendar

Observation Stage	Performance Status	Blood biochemistry	Tumor markers	Electrocardiogram, respiratory function	Upper gastrointestinal endoscopy	Chest X-ray, full abdominal CT Or ultrasound	Eligibility confirmation notice	Preoperative, postoperative complications	Adverse chemotherapy events	CRF- Preoperative	CRF-Intraoperative	CRF- Postoperative	CRF- treatment end	CRF- follow-up observation surgery
Selection Application	○	○	○	○	○	○								
After selection and prior to surgery							○			○				
Intraoperative period								○			○			
Early postoperative period								○				○	○	

Before postoperative first chemotherapy		○	○	○			○								
Regular chemotherapy		○	○	○						○					
Follow-up period Postoperative advanced stage	At postoperative 1 month (±7 days)	○	○	○			○		○						○
	At postoperative 3 months (±15 days)	○	○	○					○						○
	At postoperative 6 months (±15 days)	○	○	○			○		○						○
	At postoperative 9 months (±15 days)	○	○	○					○						○
	At postoperative 1 year (±15 days)	○	○	○			○		○						○
	At postoperative 15 months (±15 days)	○	○	○					○						○

	At postoperative 18 months (±15 days)	○	○	○			○		○						○
	At postoperative 21 months (±15 days)	○	○	○					○						○
	At postoperative 2 years (±15 days)	○	○	○				○	○						○
	At postoperative 2 years (±15 days)	○	○	○				○	○						○
	At postoperative 3 years (±15 days)	○	○	○				○	○						○

○: must do

#: Telephone follow-up procedures were added to the protocol for visits unable to be conducted due to COVID-19.

9.8 Definitions involved in SOP

9.8.1 ECOG performance status score

According to the simplified performance status score scale developed by the ECOG, the patients' performance status can be classified into 6 levels, namely 0-5, as follows:

0: Fully active, able to carry on all pre-disease performance without restriction

1: Restricted in physically strenuous activity but ambulatory and able to carry out work of

a light or sedentary nature, e.g., light housework, office work

2: Ambulatory and capable of all self-care but unable to carry out any work activities. Up and about more than 50% of waking hours

3: Capable of only limited self-care, confined to bed or chair more than 50% of waking hours

4: Completely disabled. Cannot carry on any self-care. In total, confined to bed or chair

5: Dead

Patients at levels 3, 4 and 5 are generally considered to be unsuitable for surgical treatment or chemotherapy.

9.8.2 ASA classification

According to the patients' physical status and surgical risk before anesthesia, the American Society of Anesthesiologists (ASA) has categorized patients into 5 levels (I-V levels):

Class I: Well-developed patients with physical health and normal function of various organs, with a perioperative mortality rate of 0.06% -0.08%.

Class II: Patients with mild complications and good functional compensation in addition to surgical diseases, with a perioperative mortality rate of 0.27% -0.40%.

Class III: Patients with severe complications and restricted physical activity but still capable of coping with day-to-day activities, with a perioperative mortality rate of 1.82% -4.30%.

Class IV: Patients with serious complications who have lost the ability to perform day-to-day activities, often have life-threatening conditions, and a perioperative mortality rate of 7.80% -23.0%.

Class V: Moribund patients either receiving surgery or not, have little chance for survival, and a perioperative mortality rate of 9.40% -50.70%.

Generally, Class I/II patients are considered good for anesthesia and surgical tolerance, with a smooth anesthesia process. Class III patients are exposed to some anesthesia risks; therefore, good preparations should be fully made before anesthesia, and effective measures should be taken to prevent potential complications during anesthesia. Class IV patients are exposed to the most risks, even if good preoperative preparations are made, and have a very high perioperative mortality rate. Class V patients are moribund patients and should not undergo an elective surgery.

9.8.3 Oncology-related definitions

In this study, tumor staging is based on AJCC-8th; surgical treatment follows the Japanese Gastric Cancer Treatment Guidelines, Physicians Edition, 4rd Edition, 2014, and other writing and recording principles follow the Japanese Gastric Cancer Statute 15th.

9.8.3.1 Tumor staging record

9.8.3.1.1 Recording principle

The two staging records for clinical classification and pathological classification involve T (invasion depth), N (regional lymph node) and M (distant metastasis), which are expressed in Arabic numerals and denoted as x if indefinite.

Clinical classification	Pathological classification
Physical examination X-ray, endoscopy, diagnostic imaging<br>laparoscopy, intraoperative observations (laparotomy/laparoscopy), biopsy, cytology, biochemistry, biology examination	Pathological diagnosis of the endoscopic/surgical specimens<br>Intraperitoneal exfoliative cytology

9.8.3.1.2 Records of tumor invasion depth

Tumor invasion depth is defined as follows:

TX: Unknown cancer invasion depth

T0: No cancer found

T1: Cancer invasion is only confined to the mucosa (M) or the submucosal tissue (SM)

- T1a: Cancer invasion is only confined to the mucosa (M)
- T1b: Cancer invasion is confined to the submucosal tissue (SM)

T2: Cancer invasion exceeds the submucosal tissue but is only confined to the inherent muscular layer (MP)

T3: Cancer invasion exceeds the inherent muscular layer (MP) but is only confined to the subserosal tissue (SS)

T4: Cancer invasion involves the serosa (SE) or direct invasion of adjacent structures (SI)

- T4a: Cancer invasion involves only the serosa (SE)
- T4b: Cancer directly invades the adjacent structures (SI)

9.8.3.1.3 Records of tumor metastasis

(1) Lymph node metastasis:

NX: Number of lymph node metastases is unknown

N0: No lymph node metastasis

N1: Lymph node metastasis of 1-2 areas

N2: Lymph node metastasis of 3-6 areas

N3: Lymph node metastasis of 7 and more areas

- N3a: Lymph node metastasis of 7-15 areas
- N3b: Lymph node metastasis of 16 and more areas

Lymph node numbers are defined as follows:

No.	Name	Definition
1	Cardia right	Lymph nodes around the gastric wall first branch (cardia branch) of ascending branches of the left gastric artery and those at the cardia sides
2	Cardia left	Lymph nodes at the left side of the cardia and those along the cardia branch of the lower left diaphragmatic artery esophagus
3a	Lesser gastric curvature (along the left gastric artery)	Lymph nodes at the lesser curvature side along the left gastric artery branch, below the cardia branch
3b	Lesser gastric curvature (along the right gastric artery)	Lymph nodes at the lesser curvature side along the right gastric artery branch, partial left side of the 1st branch in the lesser curvature direction
4sa	Left side of the greater gastric curvature (short gastric artery)	Lymph nodes along the short gastric artery (excluding the root)
4sb	Left side of the	Lymph nodes along the left gastroepiploic artery and the first

	greater gastric curvature (along the left gastroepiploic artery)	branch of the greater curvature (refer to the definition of No. 10)
4d	Right side of the greater gastric curvature (along the right gastroepiploic artery)	Lymph nodes at the partial left side of the first branch in the greater gastric curvature direction along the right gastroepiploic artery
5	Superior pylorus	Lymph nodes along the right gastric artery and around the first branch in the lesser gastric curvature direction
6	Inferior pylorus	Lymph nodes from the root of the right gastroepiploic artery to the first branch in the greater gastric curvature direction and those at the junction of the right gastroepiploic veins and superior anterior pancreaticoduodenal veins (including the junction portion)
7	Left gastric artery trunk	Lymph nodes from the root of the left gastric artery to the branch portion of the ascending branches
8a	Anterior upper part of the common hepatic artery	Lymph nodes at the anterior upper part of the common hepatic artery (from the branch portion of the splenic artery to the branch portion of the gastroduodenal artery)
8p	Posterior part of the common hepatic artery	Lymph nodes at the posterior part of the common hepatic artery (from the branch portion of the splenic artery to the branch portion of the gastroduodenal artery)
9	Surrounding of the celiac	Lymph gland that is in the surroundings of the celiac artery or that is a part of each root of the left artery of the stomach, common

	artery	hepatic artery and splenic artery as well as that related to the celiac artery
10	Splenic hilum	Lymph gland that is in the surroundings of the celiac artery and splenic hilum far away from the end of the pancreas, including the first greater gastric curvature in the root of the short gastric artery and the left gastroepiploic artery
11p	Splenic artery proximal	Lymph gland at the splenic artery proximal (in a location that divides the distance between the root of the splenic artery and the end of the pancreas into two equal parts, including the proximal side)
11d	Splenic artery distal	Lymph gland at the splenic artery distal (in a location that divides the distance between the root of the splenic artery and the end of the pancreas into two equal parts, inclining to the end of the pancreas)
12a	Within the hepatoduodena l ligament (along the proper hepatic artery)	Lymph gland that is below a location that divides the height of the confluence portions of the left and right hepatic ducts and the bile duct in the upper margin of the pancreas into two equal parts and is along the proper hepatic artery (as stated in No. 12a2 of the regulations for bile duct carcinoma)
12b	Within the hepatoduodena l ligament (along the bile duct)	Lymph gland that is below a location that divides the height of the confluence portions of the left and right hepatic ducts and the bile duct in the upper margin of the pancreas into two equal parts and is along the proper hepatic artery (as stated in No. 12b2 of the regulations for bile duct carcinoma)
12p	Within the hepatoduodena l ligament	Lymph gland that is below a location that divides the height of the confluence portions of the left and right hepatic ducts and the bile duct in the upper margin of the pancreas into two equal parts and

	(along the portal vein)	is along the proper hepatic artery (as stated in No. 12p2 of the regulations for bile duct carcinoma)
13	Back of the pancreatic head	Lymph gland adjacent to the head of the duodenal papilla at the back of the pancreatic head (No. 12b in the surroundings of the hepatoduodenal ligament)
14v	Along the superior mesenteric vein	Lymph gland that is in the front of the superior mesenteric vein, with the inferior margin of the pancreas on the upper side, the right gastroepiploic vein and confluence portion of the superior pancreaticoduodenal vein to the right, the left margin of the mesenteric vein to the left and the branch of the middle colic vein in the lower margin
14a	Along the superior mesenteric artery	Lymph gland along the superior mesenteric artery
15	Surroundings of the colon middle artery	Lymph gland that is in the surroundings of the colon middle artery
16a1	Surroundings of the abdominal aorta a1	Lymph gland that is in the surroundings of the aorta gap (4 to 5 cm wide in the surroundings of the medial crus of the diaphragm)
16a2	Surroundings of the abdominal aorta a2	Lymph gland that is in the surroundings of the aorta from the upper margin of the abdominal artery root to the lower margin of the left renal vein
16b1	Surroundings of the abdominal aorta b1	Lymph gland that is in the surroundings of the aorta from the lower margin of the left renal vein to the upper margin of the inferior mesenteric artery root
16b2	Surroundings of the	Lymph gland that is in the surroundings of the aorta from the upper margin of the inferior mesenteric artery root to the branch of aorta

	abdominal aorta b2	
17	Front of the pancreatic head	Lymph gland that is in the front of the pancreatic head, next to the pancreas and under the pancreatic capsule
18	Below the pancreas	Lymph gland that is in the lower margin of the pancreas
19	Below the diaphragm	Lymph gland that is in the cavity of the diaphragm and along the lower side of the diaphragmatic artery
20	Hiatal part of the gullet	Lymph gland that connects the hiatal part of diaphragm to the gullet
110	Beside the lower gullet	Lymph gland that departs from the diaphragm and is next to the lower gullet
111	Above the diaphragm	Lymph gland that is in the cavity of the diaphragm and departs from the gullet (No. 20 that connects to the diaphragm and gullet)
112	Posterior mediastinum	Lymph gland of the posterior mediastinum departed from the gullet and its hiatal portion

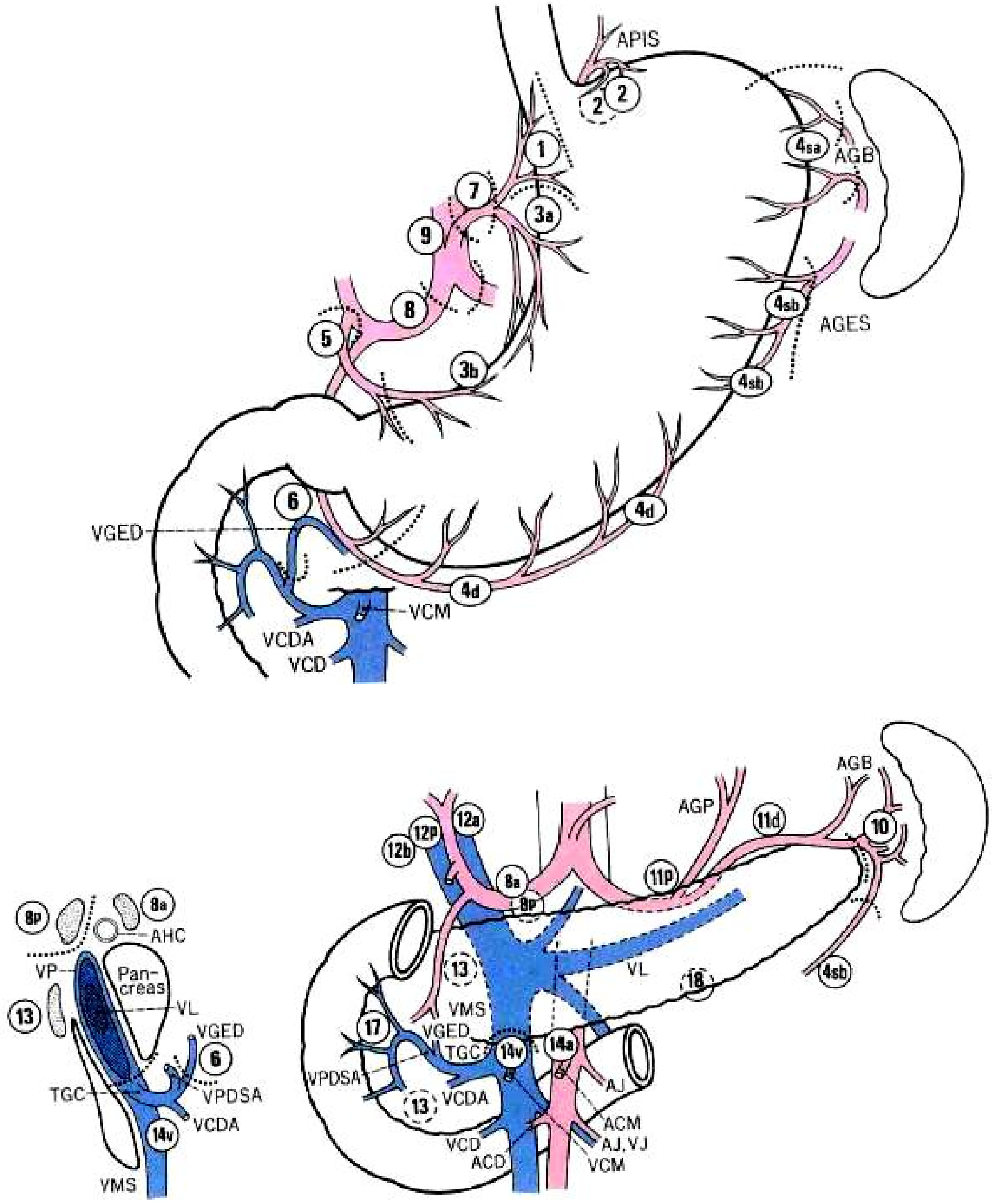

血管名
略字説明

APIS	…… *A. phrenica inferior sinistra*	AHC	……… *A. hepatica communis*
AGB	…… *Aa. gastricae breves*	VP	………… *V. portae*
AGES	…… *A. gastroepiploica sinistra*	VL	………… *V. lienalis*
VGED	…… *V. gastroepiploica dextra*	VMS	……… *V. mesenterica superior*
VCDA	…… *V. colica dextra accessoria*	VPDSA	…… *V. pancreaticoduodenalis superior anterior*
VCM	…… *V. colica media*	VCD	……… *V. colica dextra*
TGC	…… *Truncus gastrocolicus*	VJ	………… *V. jejunalis*
ACM	…… *A. colica media*	AGP	……… *A. gastrica posterior*
AJ	……… *A. jejunalis*	ACD	……… *A. colica dextra*

Fig. 4. Lymph node grouping

(2) Distant metastasis

M0: No distant metastasis outside of the regional lymph nodes

M1: Distant metastasis outside of the regional lymph nodes

MX: Presence of distant metastasis is unclear

Record the specific sites under the M1 condition: peritoneum (PER), liver (HEP), lymph node (LYM), skin (SKI), lung (PUL), bone marrow (MAR), bone (OSS), pleura (PLE), brain (BRA) and meninges (MEN), intraperitoneal exfoliated cells (CY), and others (OTH). Note: A positive examination result for intraperitoneal exfoliated cells is recorded as M1.

9.8.3.1.4 Tumor Staging

Pathological (pTNM)					
T/M	N0	N1	N2	N3a	N3b
T1	IA	IB	IIA	IIB	IIIB
T2	IB	IIA	IIB	IIIA	IIIB
T3	IIA	IIB	IIIA	IIIB	IIIC
T4a	IIB	IIIA	IIIA	IIIB	IIIC
T4b	IIIA	IIIB	IIIB	IIIC	IIIC
M1	IV	IV	IV	IV	IV

9.8.3.2 Pathologic types and classifications

9.8.3.2.1 Type

Papillary adenocarcinoma

Tubular adenocarcinoma

Mucinous adenocarcinoma

Signet ring cell carcinoma

Poorly differentiated carcinoma

9.8.3.2.2 Grading

GX classification is not possible to assess

G1 well-differentiated

G2 moderately differentiated

G3 poorly differentiated

G4 undifferentiated

9.8.3.3 Evaluation of Radical Level (Degree)

9.8.3.3.1 Recording the Presence or Absence of Cancer Invasion on the Resection Stump

(1) Proximal incisional margin (PM: proximal margin)

PM (-): No cancer invasion found on the proximal incisional margin

PM (+): Cancer invasion found on the proximal incisional margin

PM X: Unknown cancer invasion on the proximal incisional margin

(2) Distal incisional margin (DM: distal margin)

DM (-): No cancer invasion found on the distal incisional margin

DM (+): Cancer invasion found on the distal incisional margin

DM X: Unknown cancer invasion on the distal incisional margin

9.8.3.3.2 Radical Records

Postoperative residual tumor, denoted with R (residual tumor): R0: curative resection; R1, R2: non-curative resection.

RX: cannot be evaluated

R0: no residual cancer

R1: microscopic residual cancer (positive margins, peritoneal lavage cytology positive)

R2: macroscopic residual cancer

10 Statistical analysis

10.1 Definition of the population

(1) ITTP, intent-to-treat population

(2) MITTP, modified intent-to-treat population

(3) PPP, per-protocol population

(4) SAP, safety analysis population

10.2 Statistical analysis plan

- Statistical software: We will use Epidata3.0 to establish a database and to input data， and we will use SPSS18.0 software to perform statistical analyses.
- Basic principle：The method of differential testing was adopted. The safety population of the study consists of the patients who receive safety evaluation data after the intervention. Descriptive statistics and two-sided tests were conducted for the safety indicators and the incidence of adverse reactions. A p-value <0.05 is considered statistically significant. The confidence interval of the parameters is estimated with a 95% confidence interval.
- Shedding analysis: Total shedding rate of two groups and loss rate due to adverse

events will be compared using pearsonχ^2 test

- Statistical analysis of population division: baseline data and effective analysis using MITT analysis. The main therapeutic indicators are analyzed using both MITT and PP analysis. But based on the conclusion of MITT analysis. If MITT analysis and PP analysis of the conclusions are consistent, it can increase the credibility of the conclusion. The data of laboratory examination, adverse events and adverse reactions were analyzed by SAP. The incidence rate of adverse reactions uses SAP as the denominator. The long-term outcomes are analyzed using PP analysis.
- Method of outlier determination: the observation value is greater than P75 or less than P25, and the exceed value more than 3 times of the quartile spacing (=p75-p25), which will be sentenced to outlier data. During the analysis, the sensitivity analysis is used for outlier data, namely analyzing outcomes including or excluding, outliers data. and if the results are not contradictory, the data is retained; if the contradiction, it depends on the specific circumstances.
- Descriptive statistics: The measurement data gives the mean, the standard deviation and the confidence interval, and the minimum value, the maximum value, the P25, the median and the P75 are given when necessary; matched data also gives the mean and standard deviation of the gap-value, and the median and average rank of the Non-parametric method. The nominal-scale data gives the frequency distribution and the corresponding percentages. The level data gives the frequency distribution and the corresponding percentages, as well as the median and the average rank. Qualitative data give positive rate, positive number, and denominator numbers. The survival data gives the number of events, the number of deletions, the median survival time, and the survival rate.
- Subgroup analysis： Sub-group analysis is to find the factors that may affect prognostic according to the specific circumstances of the data. For example, subgroup analyses, using log-rank tests, were conducted for disease-free and overall survival stratified by pathologic T stage (ie, pT1, pT2-4) and the LN status (ie, pN0, pN+).
- Missing values handling：This study does not fill in missing values
- Effective analysis: Using Log-rank test for single factor analysis of Survival Time

Data, using Cox regression model Analysis for multi-factor analysis. Quantitative data using t test or t' Test (variance is not homogeneous), qualitative data using Pearson χ^2 test, grade data using Wilcoxon rank test.

- Safety analysis: counting adverse responds incidence and incidence of adverse events and make a list to describe the adverse events occurring in the study. describe the results of the laboratory tests before and after the normal/abnormal changes and the relationship between the abnormal changes and drugs in the research, and make a list on the "normal/abnormal" changes occurred in the study.. More detailed statistical analysis is shown in the statistical analysis plan.

11 Data management

11.1 Case Report Form (CRF)

11.1.1 CRF Types and Submission Deadline

CRFs used in this study and their submission deadlines are as follows:

(1) Case Screening: 7 days prior to surgery (time frame of 3 days)

(2) Enrolling: submitted to the data center at one day prior to surgery

(3) Surgery: within 1 day after surgery

(4) Postoperative discharge: within 3 days after the first discharge

(5) Follow-up records: 7 days after each specified follow-up time point

11.1.2 Method of transmission of CRF

In this study, the paper CRF form are used for information and data transmittal.

11.1.3 Revision of CRF

After the start of the study, if the CRF is found to lack items that are then deemed pertinent, under the premises of ensuring the amendment of the CRF does not cause medical and economic burden and increased risks to the selected patients, the CRF can be modified after the Research Committee adopt it through discuss at the meeting. If the amendment of the CRF requires no changes to this study protocol, the latter will not be modified.

11.2 Monitoring and Supervising

To assess whether study implementation follows protocol and data are being collected properly, monitoring should be conducted every February during the follow-up period. Monitoring is to complete through visiting a hospital and comparing

the original Data. Data Safety Monitoring Board (DSMB) was responsible by Mi Lin who was medical doctor (M.D.) from Fujian Medical University Union Hospital. The DSMB will meet at least annually after study initiation to assess enrollment, retention (drop-out and drop-in rates), and safety data, and may meet more frequently if needed.

11.2.1 Monitoring item

- Data Collection Completion Status: By selected registration numbers (cumulative and for each time period)
- Eligibility: Not eligible patients/potentially ineligible patients
- Different end of treatment, the reasons for suspension/end of the study protocol
- Background factors, pre-treatment report factors, post-treatment report factors when selected for registration
- Severe adverse events
- Adverse events/adverse reactions
- Laparoscopic surgery completion percentage
- Proportion of conversion to laparotomy
- Protocol deviation
- Disease-free survival /overall survival (all enrolled Patients)
- Progress and safety of the study, other issues

11.2.2 Acceptable range of adverse events

Treatment-related death and life-threatening complications caused by surgeries occur relatively rarely and partly are dependent on the qualifications of the research participating hospitals and their staff; a rate of over 3% is considered unacceptable. If treatment-related death is suspected or non-hematologic Grade 4 toxicity having a causal relationship with the surgery is determined, adverse events should be reported to the Efficacy and Safety Evaluation Committee. If the number of treatment-related deaths or the number of patients with determined non-hematologic Grade 4 toxicity having a causal relationship with the surgery reached 15, the final incidence proportion of adverse events would be expected to exceed 3%, and therefore the inclusion of patients must be immediately suspended. Whether the study can continue should be determined by the Efficacy and Safety Evaluation Committee.

12 Relevant Provisions on adverse events

12.1 Surgery-related adverse events

See the adverse events mentioned for surgical complications in 8.1 Definition of the study endpoint.

12.2 Various forms of adverse events caused by original incidence

Adverse events relating to various forms of deterioration in primary diseases should be recorded according to Short Name of CTCAEv3.0.

12.3 Evaluation of adverse events

- Evaluation of adverse event/adverse reaction are based on[Accordion Severity Grading System] and [CTCAE v3.0].
- Adverse events will be graded 0 ~ 4 as per definition. For treatment-related death, fatal adverse events are classified as Grade 5 in the original CTCAE
- Toxicity items specified in the [surgery-related adverse events], Grade and the discovery date of Grade should be recorded in the treatment process report. For other toxicity items observed, observed Grade 3 toxicity items are only recorded in the freedom registration column of the treatment process report, as well as Grade and the discovery date of Grade. Grade recorded in the treatment process report must be recorded in the case report form.
- CTCAE v3.0, the so-called “Adverse Event”, “all observed, unexpected bad signs, symptoms and diseases (abnormal value of clinical examination are also included) in the treatment or disposal, regardless of a causal relationship with the treatment or handling, including determining whether there is a causal relationship or not”.
- Therefore, even if events were “obviously caused by primary disease (cancer)” or caused by supportive therapy or combination therapy rather than the study regimen treatment (protocol treatment), they are “adverse events”.
- For adverse event data collection strategy, the following principles should be complied with in this study:1) Adverse events within 30 days from the last treatment day of the study regimen treatment (protocol treatment), regardless of the presence or absence of a causal relationship should be completely collected. (When adverse events are reported, the causality and classification of adverse events are separately

discussed) 2) Adverse events within 30 days from the last treatment day of the study regimen treatment (protocol treatment), regardless of the presence or absence of a causal relationship should be completely collected. (When adverse events are reported, the causality and classification of adverse events are separately discussed)

12.4 Reporting of Adverse Events

- When “severe adverse events” or “unexpected adverse events” occur, the Research Responsible Person of research participating unit should report them to the Research Committee (Chang-Ming Huang).
- Based on the relevant laws and regulations, adverse events should be reported to the province (city) Health Department at the location of each research center. Severe adverse events based on clinical research-related ethical guideline should be reported to the person in overall charge of the medical institution. The appropriate reporting procedures should be completed in accordance with the relevant provisions of all medical institutions at the same time. The person in charge of research of each research participating unit should hold accountability and responsibility for the emergency treatment of patients with any degree of adverse events to ensure patient safety.

12.4.1 Adverse Events with Reporting Obligations

12.4.1.1 Adverse Events with Emergency Reporting Obligations

Any of the following adverse events should be reported on an emergent basis:

- All patients who die during the course of treatment or within 30 days from the last treatment day, regardless of the presence or absence of a causal relationship with the study regimen treatment. Also, cases of discontinuation of treatment, even if within 30 days from the last treatment day, those patients are also emergent reporting objects. (“30 days” refers to day 0, the final treatment day, 30 days starting from the next day)
- Those patients with unexpected Grade 4 non-hematologic toxicity (CTCAE v3.0 adverse events other than the blood/bone marrow group), having a causality of treatment (any of definite, probable, possible) who emergent reporting objects are.

12.4.1.2 Adverse Events with Regular Reporting Obligations

One of the following adverse events are regular reporting objects:

(1) After 31 days from the last treatment day, deaths for which a causal relationship with treatment cannot be denied, including suspected treatment-related death; death due
to obvious primary disease is included.
(2) Expected Grade 4 non-hematologic toxicity (CTCAE v3.0 adverse events other than the blood/bone marrow group).
(3) Unexpected Grade 3adverse events: Grade 3 adverse events are not recorded in the
12.1 expected adverse events.
(4) Other significant medical events: adverse events that the study group deems cause Important and potentially permanent, significant impact on their offspring (MDS myelodysplastic syndrome, except for secondary cancer) Adverse events among above (2)-(4), determined to have a causal relationship (any of definite, probable, possible) with the study regimen are regular reporting objects.

12.4.2 Reporting Procedure

12.4.2.1 Emergency Reporting

- In case of any adverse event on emergency study reporting objects, the doctor in charge will quickly report it to the Research Responsible Person of the research participating hospitals. When the Research Responsible Person of the hospital cannot be contacted, the coordinator or the doctor in charge of the hospital must assume the responsibility on behalf of the Research Responsible Person of the hospital.
- First Reporting: Within 72 hours after the occurrence of adverse events, the Research Responsible Person of the hospital should complete the "AE/AR/ADR first emergency report" and send it to the Research Committee by email and telephone.
- Second Reporting: The Research Responsible Person of each research participating hospital completes the "AE/AR/ADR Report" and a more detailed case information report (A4 format), and then faxes the two reports to the Research Committee within 15 days after the occurrence of adverse events. If any autopsy examination, the autopsy result report should be submitted to the Research Committee.

12.4.2.2 General Reports

- The Research Responsible Person of each research participating hospital completes the "AE/AR/ADR report", and then faxes it to the Research Committee within 15 days after the occurrence of adverse events.

12.5 Review of Efficacy and Safety Evaluation Committee

The Efficacy and Safety Evaluation Committee reviews and discusses the report in accordance with the procedures recorded in the *Clinical Safety Information Management Guideline*, and makes recommendations in writing for the Research Responsible Person, including whether to continue to include study objects or to modify the study protocol.

13 Ethical Considerations

13.1 Responsibilities of researchers

The investigators are responsible for the conduction of this study at their centers. The investigators will ensure the implementation of this study in accordance with the study protocol and in compliance with the Declaration of Helsinki, as well as domestic and international ethical guiding principles and applicable regulatory requirements. It is specially noted that, the investigators must ensure that only subjects providing informed consent can be enrolled in this study.

13.2 Information and Informed Consent of Subjects

An unconditional prerequisite for subjects to participate in this study is his/her written informed consent. The written informed consent of subjects participating in this study must be given before study-related activities are conducted.

Therefore, before obtaining informed consent, the investigators must provide sufficient information to the subjects. In order to obtain the informed consent, the investigators will provide the information page to subjects, and the information required to comply with the applicable regulatory requirements. While providing written information, the investigators will orally inform the subjects of all the relevant circumstances of this study. In this process, the information must be fully and easily understood by non-professionals, so that they can sign the informed consent form according to their own will on the basis of their full understanding of this study.

The informed consent form must be signed and dated personally by the subjects and investigators. All subjects will be asked to sign the informed consent form to prove

that they agree to participate in the study. The signed informed consent form should be kept at the research center where the investigator is located and must be properly safe kept for future review at any time during audit and inspection throughout the inspection period. Before participating in the study, the subjects should provide a copy of signed and dated informed consent form.

At any time, if important new information becomes available that may be related to the consent of the subjects, the investigators will revise the information pages and any other written information which must be submitted to the IEC/IRB for review and approval. The revised information approved will be provided to each subject participating the study. The researchers will explain the changes made to the previous version of ICF to the subjects

13.3 Identity and Privacy of Subjects

After obtaining an informed consent form, each selected subject is assigned a subject number (Allocation Number). This number will represent the identity of the subject during the entire study and for the clinical research database of the study. The collected data of subjects in the study will be stored in the ID.

Throughout the entire study, several measures will be taken to minimize any breaches of personal information, including: 1) only the investigators will be able to link to the research data of the subjects to themselves through the identify table kept at the research center after authorization; 2) during onsite auditing of raw data by the supervisors of this study, as well as relevant inspection and inspection visits by the supervision departments, the personnel engaging in the above activities may view the original medical information of subjects that will be kept strictly confidential.

Collection, transmission, handling and storage of data on study subjects will comply with the data protection and privacy regulations. This information will be provided to the study subjects when their informed consent is being obtained for treatment procedures in accordance with national regulations.

13.4 Independent Ethics Committee or Institutional Review Committee

Before beginning the study, the Research Center will be responsible for submitting the study protocol and relevant documents (informed consent form, subject information page, CRF, and other documents that may be required) to the Independent Ethics Committee (IEC)/ Institutional Review Board (IRB) to obtain their

favorable opinion/approval. The favorable opinions/approval documents of the IEC/IRB will be archived in the research center folders of the investigators.

Before beginning the study at the center, the investigators must obtain written proof of favorable opinions/approval by the IEC/IRB, and should provide written proof of the date of the favorable opinions/approval meeting, written proof of the members presenting at the meeting and voting members, written proof of recording the reviewed study, protocol version and Informed Consent Form version, and if possible, a copy of the minutes.

In case of major revisions to this study, the amendment of the study protocol will be submitted to the IEC/IRB prior to performing the study. In the course of the study, the relevant safety information will be submitted to the IEC/IRB in accordance with national regulations and requirements.

13.5 Supervising

The research approach of the authorities and any associated files (such as the research protocol, subjects' informed consent) will be in accordance with the requirements of the ethical review board of biomedical research involving humans (Trial) (2007) and the applicable Chinese laws and regulations. Studies should provide the main references or inform the ethics review guidance advisory organization of the provincial health administrative department in the province the research center is in.

14 Organizations and Responsibilities of Study

14.1 Research Committee

- Responsible for developing study protocol, auditing eligibility for inclusion and guiding the interpretation of informed consent; also responsible for the collection of adverse event reports, guiding the clinical diagnosis and treatment of such events and the emergency intervention of serious adverse events.
- Person in Charge of Research Committee: Changming Huang （Department of Gastric Surgery, Fujian Medical University Union Hospital）

  Add: Department of Gastric Surgery, Fujian Medical University Union Hospital, No.29 Xinquan Road, Fuzhou 350001, Fujian Province, China.;Post code:350001;Tel:0591-83357896-8011;Fax:0591-83363366;Mobile:13805069676;

E-mail： hcmlr2002@163.com

- Chief Statistical Expert of Research Committee: Hu Zhijian （Department of Preventive Medicine statistics, School of Public health, Fujian Medical University）

14.2 Efficacy and Safety Evaluation Committee

Responsible for the supervision/monitoring of treatment safety and efficacy of this study.

Person in Charge of Efficacy and Safety Evaluation Committee: Changming Huang （Department of Gastric Surgery, Fujian Medical University Union Hospital）

14.3 Independent Ethics Committee/Institutional Review Board (IEC/IRB)

Responsible for evaluating this study to determine if risks to which subjects are exposed have been duly minimized and whether these risks are reasonable compared to expected benefits.

The independent Ethics Committee/Institutional Review Board (IEC/IRB) at the location of each research participating center is responsible for the ethics review of all research participating units.

15 References

[1] Parkin, DM and Muir CS. Cancer Incidence in Five Continents. Comparability and quality of data. IARC Sci Publ, 1992(120): 45-173.

[2] Bozzetti, F., et al., Subtotal versus total gastrectomy for gastric cancer: five-year survival rates in a multicenter randomized Italian trial. Italian Gastrointestinal Tumor Study Group. Ann Surg, 1999. 230(2): p. 170-8.

[3]. Jentschura, D., et al., Quality-of-life after curative surgery for gastric cancer: a comparison between total gastrectomy and subtotal gastric resection. Hepatogastroenterology,
1997. 44(16): p. 1137-42.

[4] Kitano S, Iso Y, Moriyama M, et al. Laparoscopy-assisted Billroth Ⅰ gastrectomy[J]. Surg Laparosc Endosc, 1994,4(2): 146-148.

[5] Kitano S, Shiraishi N, Fujii K, et al. A randomized controlled trial comparing open vs laparoscopy-assisted distal gastrectomy for the treatment of early gastric cancer: an

interim report[J]. Surgery, 2002,131(1 Suppl):S306-S311.
[6] Lee JH, Han HS, Lee JH. A prospective randomized study comparing open vs laparoscopy-assisted distal gastrectomy in earlygastriccancer:earlyresults[J]. Surg Endosc, 2005,19(2): 168-173.
[7] Huscher CG, Mingoli A, Sgarzini G, et al. Laparoscopic versus open subtotal gastrectomy for distal gastric cancer: five-year results of a randomized prospective trial[J]. Ann Surg, 2005,241(2):232-237.
[8] Hayashi H, Ochiai T, Shimada H, et al. Prospective randomized study of open versus laparoscopy-assisted distal gastrectomy with extraperigastric lymph node dissection for early gastric cancer[J]. Surg Endosc, 2005,19(9):1172-1176.
[9] Kim YW, Baik YH, Yun YH, et al. Improved quality of life outcomes after laparoscopy-assisted distal gastrectomy for early gastric cancer: results of a prospective randomized clinical trial[J]. Ann Surg, 2008,248(5):721-727.
[10] Kim HH, Hyung WJ, Cho GS, et al. Morbidity and mortality of laparoscopic gastrectomy versus open gastrectomy for gastric cancer: an interim report--a phaseIII multicenter, prospective, randomized Trial(KLASS Trial)[J]. Ann Surg, 2010,251(3):417-420.
[11] Song J, Lee HJ, Cho GS, et al; Korean Laparoscopic Gastrointestinal Surgery Study (KLASS) Group. Recurrence following laparoscopy-assisted gastrectomy for gastric cancer: a multicenter retrospective analysis of 1,417 patients [J]. Ann Surg Oncol, 2010,17(7):1777-1786.
[12] Scatizzi M, Kroning KC, Lenzi E, et al. Laparoscopic versus open distal gastrectomy for locally advanced gastric cancer: a case-control study[J]. 2011,63:17-23. Updates Surg, 2011,63 (1):17-23.
[13] Kojima K, Yamada H, Inokuchi M, et al. Current status and evaluation of laparoscopic surgery for gastric cancer [J]. Nihon Geka Gakkai Zasshi, 2006,107(2):77-80.
[14] Hu Y, Huang C, Sun Y, et al. Morbidity and Mortality of Laparoscopic Versus Open D2 Distal Gastrectomy for Advanced Gastric Cancer: A Randomized Controlled Trial. J Clin Oncol. 2016,34(12):1350-1357.
[15] Cadière GB, Himpens J, Germay O, et al. Feasibility of robotic laparoscopic surgery: 146 cases[J]. World J Surg, 2001,25(11):1467-1477.

[16] Patriti A, Ceccarelli G, Ceribelli C, et al. Robot-assisted laparoscopic management of cardia carcinoma according to Siewert recommendations [J]. Int J Med Robot, 2011,7(2):170-177.
[17] Oleynikov D. Robotic surgery[J]. Surg Clin North Am, 2008,88(5):1121-1130, viii.
[18] Lanfranco AR, Castellanos AE, Desai JP, et al. Robotic surgery: a current perspective[J]. Ann Surg, 2004,239(1):14-21.
[19] Kaushik D, High R, Clark CJ, et al. Malfunction of the Da Vinci robotic system during robot-assisted laparoscopic prostatectomy: an international survey [J]. J Endourol, 2010,24(4):571-575.
[20] Hashizume M, Shimada M, Tomikawa M, et al. Early experiences of endoscopic procedures in general surgery assisted by a computer-enhanced surgical system[J]. Surg Endosc, 2002, 16(8):1187-1191.
[21] Liu Guoxiao, Shen Weisong, Chen Lin, Wei Bo. Robotic versus laparoscopic gastrectomy for gastric cancer: a meta-analysis. Chin J Gastrointest Surg, 2016,19(3):328-333.
[22] Kim HI, Han SU, Yang HK, et al. Multicenter Prospective Comparative Study of Robotic Versus Laparoscopic Gastrectomy for Gastric Adenocarcinoma. Ann Surg. 2016,263(1):103-109.

16 Annex

16.1 Informed Consent Form

Summary of changes to the protocol approved by the IRB

All procedure changes were adjudicated with the IRBs and added in the initial approval process to start the trial before any enrollment.

1. The Clinicaltrials.gov number and the IRB approval numbers were added.
2. The DSMB was named.
3. Time point of randomization were added.
4. The information of postoperative adjuvant chemotherapy was updated.
5. The information of statistical analysis of population division was updated.
6. A version number was added.
7. Added drop-in / drop-out definitions, and follow-up requirement for participants who cannot attend visits.
8. Actual follow-up time was further clarified.
9. The contents of requirement for the retention follow up call was recommended.
10. Data on COVID-19 diagnoses (suspected and confirmed) will be collected as routine adverse events, for the purpose of identifying cases in the future as needed for ancillary research proposals in development.
11. Telephone follow-up procedures were added to the protocol for visits unable to be conducted due to COVID-19. Missed clinic visits were to be reported as such and considered protocol deviations.
12. Outcome analysis were updated, including the results of subgroup analysis for patients with different pathologic T stage (ie, pT1, pT2-4) and the LN status (ie, pN0, pN+).

Original Statistical Analysis Plan

Randomized Controlled Trials on Clinical Outcomes of Robotic versus Laparoscopic Distal Gastrectomy for Gastric Cancer (FUGES-011)

Chang-Ming Huang, M.D., FACS

Department of Gastric Surgery, Fujian Medical University Union Hospital

Study Objective

To investigate the safety, feasibility and long-term outcome of robotic distal gastrectomy versus laparoscopic distal gastrectomy for gastric cancer

Primary Outcome Measures：

- 3-year disease free survival rate

Secondary Outcome Measures：

- 3-year overall survival rate
- 3-year recurrence pattern
- Overall postoperative morbidity rates
- Intraoperative morbidity rates
- Overall postoperative serious morbidity rates
- Number of retrieved lymph nodes
- Noncompliance rate of lymphadenectomy
- Time to first ambulation
- Time to first flatus
- Time to first liquid diet
- Time to first soft diet
- Duration of postoperative hospital stay
- The variation of weight
- The variation of cholesterol
- The variation of album
- The variation of white blood cell count
- The variation of hemoglobin
- Hospitalization expenses

- Operation time

Randomization

Eligible patients were randomly assigned by a 1:1 ratio to either the RDG or LDG group. The data manager, who was not involved in the eligibility assessment and recruitment of patients, performed randomization with a list of randomly ordered treatment identifiers generated by a permuted block design using SAS (version 9.2; SAS Institute Inc.). The allocation sequence was concealed from the surgeons who enrolled the patients until they were formally randomized to their groups. However, it was not feasible to blind the surgeons and participants owing to the nature of the surgical clinical trial.

Data Management

In this study, the paper CRF form are used for information and data transmittal. After the start of the study, if the CRF is found to lack items that are then deemed pertinent, under the premises of ensuring the amendment of the CRF does not cause medical and economic burden and increased risks to the selected patients, the CRF can be modified after the Research Committee adopt it through discuss at the meeting. If the amendment of the CRF requires no changes to this study protocol, the latter will not be modified. To assess whether study implementation follows protocol and data are being collected properly, monitoring should be conducted every February during the follow-up period. Monitoring is to complete through visiting a hospital and comparing the original Data.

Sample size

This study is a non-inferior test (bilateral), whose primary outcome measure is 3-year disease free survival. According to the previous study results and related literature reports, the projected 3-year DFS rate for the LDG group was 82.3%. Based on an α of 0.025, a power of 90%, and a margin delta of 16%, we determined that at least 120 patients should be included each group. Considering an expected dropout rate of 20%, a total of 300 patients were needed.

Statistical Analysis

- Statistical software: We will use Epidata 3.0 to establish a database and to input data， and we will use SPSS 18.0 software to perform statistical analyses.
- Basic principle: The method of differential testing was adopted. The safety population of the study consists of the patients who receive safety evaluation data after the intervention. Descriptive statistics and two-sided tests were conducted for the safety indicators and the incidence of adverse reactions. A *P*-value <0.05 is considered statistically significant. The confidence interval of the parameters is estimated with a 95% confidence interval.
- Shedding analysis: Total shedding rate of two groups and loss rate due to adverse events will be compared using χ^2 test
- Statistical analysis of population division: baseline data and effective analysis using MITT analysis. The main therapeutic indicators are analyzed using both MITT and PP analysis. But based on the conclusion of PP analysis. If MITT analysis and PP analysis of the conclusions are consistent, it can increase the credibility of the conclusion. The data of laboratory examination, adverse events and adverse reactions were analyzed by SAP. The incidence rate of adverse reactions uses SAP as the denominator.
- Method of outlier determination: the observation value is greater than P75 or less than P25, and the exceed value more than 3 times of the quartile spacing (=P75-P25), which will be sentenced to outlier data. During the analysis, the sensitivity analysis is used for outlier data, namely analyzing outcomes including or excluding, outliers data. and if the results are not contradictory, the data is retained; if the contradiction, it depends on the specific circumstances.
- Descriptive statistics: The measurement data gives the mean, the standard deviation and the confidence interval, and the minimum value, the maximum value, the P25, the median and the P75 are given when necessary; matched data also gives the mean and standard deviation of the gap-value, and the median and average rank of the non-parametric method. The nominal-scale data gives the frequency distribution and the corresponding percentages. The level data gives the frequency distribution and the corresponding percentages, as well as the median and the average rank. Qualitative data give positive rate, positive number,

and denominator numbers. The survival data gives the number of events, the number of deletions, the median survival time, and the survival rate.

- Subgroup analysis: Sub-group analysis is to find the factors that may affect prognostic according to the specific circumstances of the data.
- Missing values handling: This study does not fill in missing values
- Effective analysis: Using Log-rank test for single factor analysis of survival time data, using Cox regression model analysis for multi-factor analysis. Quantitative data using t test or t' test (variance is not homogeneous), qualitative data using Pearson χ^2 test, grade data using Wilcoxon rank test.
- Safety analysis: counting adverse responds incidence and incidence of adverse events and make a list to describe the adverse events occurring in the study. describe the results of the laboratory tests before and after the normal/abnormal changes and the relationship between the abnormal changes and drugs in the research, and make a list on the "normal/abnormal" changes occurred in the study. More detailed statistical analysis is shown in the statistical analysis plan.

Final Statistical Analysis Plan

Statistical Analysis Plan for

Randomized Controlled Trials on Clinical Outcomes of Robotic versus Laparoscopic Distal Gastrectomy for Gastric Cancer (FUGES-011)

Study protocol

Overall Principal Investigator (PI), Fujian Medical University PI:
Chang-Ming Huang, M.D.
Department of Gastric Surgery
Fujian Medical University Union Hospital

Site PIs: Chang-Ming Huang, M.D.

Trial Sponsor: Chang-Ming Huang, M.D.

Protocol Signatures:
Chang-Ming Huang

Data Safety Monitoring Board (DSMB): Mi Lin, M.D. Department of Gastric Surgery
Fujian Medical University Union Hospital

Short title: Robotic Distal Gastrectomy Trial

Study Objective

The objective of this study is to investigate the safety, efficacy, and feasibility of ICG near-infrared imaging tracing in guiding laparoscopic D2 lymph node (LN) dissection for gastric cancer.

Inclusion Criteria

(1) Age from 18 to 75 years (not including 18 and 75 years old)

(2) Primary gastric adenocarcinoma (papillary, tubular, mucinous, signet ring cell, or poorly differentiated) confirmed pathologically by endoscopic biopsy

(3) Clinical stage tumor T1-4a (cT1-4a), N-/+, M0 at preoperative evaluation according to the American Joint Committee on Cancer (AJCC) Cancer Staging Manual Eighth Edition

(4) Expected to undergo distal gastrectomy and D1+/D2 lymph node dissection to obtain R0 surgical results.

(5) Performance status of 0 or 1 on the ECOG (Eastern Cooperative Oncology Group) scale

(6) ASA class I to III

(7) Written informed consent

Exclusion criteria

(1) Women during pregnancy or breast-feeding

(2) Severe mental disorder

(3) History of previous upper abdominal surgery (except for laparoscopic cholecystectomy)

(4) History of previous gastric surgery (including ESD/EMR for gastric cancer)

(5) Multiple primary gastric cancer

(6) Enlarged or bulky regional lymph node diameter over 3cm by preoperative imaging

(7) History of other malignant disease within past five years

(8) History of previous neoadjuvant chemotherapy or radiotherapy

(9) History of unstable angina or myocardial infarction within the past six months

(10) History of cerebrovascular accident within past six months

(11) History of continuous systematic administration of corticosteroids within one month

(12) Requirement of simultaneous surgery for another disease

(13) Emergency surgery due to complications (bleeding, obstruction or perforation) caused by gastric cancer

(14) FEV1 < 50% of the predicted values

Consent

An unconditional prerequisite for subjects to participate in this study is his/her written informed consent. The written informed consent of subjects participating in this study must be given before study-related activities are conducted.

Therefore, before obtaining informed consent, the investigators must provide sufficient information to the subjects. In order to obtain the informed consent, the investigators will provide the information page to subjects, and the information required to comply with the applicable regulatory requirements. While providing written information, the investigators will orally inform the subjects of all the relevant circumstances of this study. In this process, the information must be fully and easily understood by non-professionals, so that they can sign the informed consent form according to their own will on the basis of their full understanding of this study.

The informed consent form must be signed and dated personally by the subjects and investigators. All subjects will be asked to sign the informed consent form to prove that they agree to participate in the study. The signed informed consent form should be kept at the research center where the investigator is located and must be properly safe kept for future review at any time during audit and inspection throughout the inspection period. Before participating in the study, the subjects should provide a copy of signed and dated informed consent form.

At any time, if important new information becomes available that may be related to the consent of the subjects, the investigators will revise the information pages and any other

written information which must be submitted to the IEC/IRB for review and approval. The revised information approved will be provided to each subject participating the study. The researchers will explain the changes made to the previous version of ICF to the subjects

Primary Outcome Measures：

- 3-year disease free survival rate

Secondary Outcome Measures：

- 3-year overall survival rate
- 3-year recurrence pattern
- Overall postoperative morbidity rates
- Intraoperative morbidity rates
- Overall postoperative serious morbidity rates
- Number of retrieved lymph nodes
- Noncompliance rate of lymphadenectomy
- Time to first ambulation
- Time to first flatus
- Time to first liquid diet
- Time to first soft diet
- Duration of postoperative hospital stay
- The variation of weight
- The variation of cholesterol
- The variation of album
- The variation of white blood cell count
- The variation of hemoglobin
- Hospitalization expenses
- Operation time

Randomization

Eligible patients were randomly assigned by a 1:1 ratio to either the RDG or LDG group. The data

manager, who was not involved in the eligibility assessment and recruitment of patients, performed randomization with a list of randomly ordered treatment identifiers generated by a permuted block design using SAS (version 9.2; SAS Institute Inc.). The allocation sequence was concealed from the surgeons who enrolled the patients until they were formally randomized to their groups. However, it was not feasible to blind the surgeons and participants owing to the nature of the surgical clinical trial.

Data Management

In this study, the paper CRF form are used for information and data transmittal. After the start of the study, if the CRF is found to lack items that are then deemed pertinent, under the premises of ensuring the amendment of the CRF does not cause medical and economic burden and increased risks to the selected patients, the CRF can be modified after the Research Committee adopt it through discuss at the meeting. If the amendment of the CRF requires no changes to this study protocol, the latter will not be modified. To assess whether study implementation follows protocol and data are being collected properly, monitoring should be conducted every February during the follow-up period. Monitoring is to complete through visiting a hospital and comparing the original Data.

Sample size

This study is a non-inferior test (bilateral), whose primary outcome measure is 3-year disease free survival. According to the previous study results and related literature reports, the projected 3-year DFS rate for the LDG group was 82.3%. Based on an α of 0.025, a power of 90%, and a margin delta of 16%, we determined that at least 120 patients should be included each group. Considering an expected dropout rate of 20%, a total of 300 patients were needed.

Statistical Analysis

- Statistical software: We will use Epidata 3.0 to establish a database and to input data， and we will use SPSS 22.0 software to perform statistical analyses.
- Basic principle: The method of differential testing was adopted. The safety population of

the study consists of the patients who receive safety evaluation data after the intervention. Descriptive statistics and two-sided tests were conducted for the safety indicators and the incidence of adverse reactions. A *P*-value <0.05 is considered statistically significant. The confidence interval of the parameters is estimated with a 95% confidence interval.

- Shedding analysis: Total shedding rate of two groups and loss rate due to adverse events will be compared using χ^2 test
- Statistical analysis of population division: baseline data and effective analysis using MITT analysis. The main therapeutic indicators are analyzed using both MITT and PP analysis. But the conclusion based on the result of PP analysis. If MITT analysis and PP analysis of the conclusions are consistent, it can increase the credibility of the conclusion. The data of laboratory examination, adverse events and adverse reactions were analyzed by SAP. The incidence rate of adverse reactions uses SAP as the denominator. The long-term outcomes are analyzed using PP analysis.
- Method of outlier determination: the observation value is greater than P75 or less than P25, and the exceed value more than 3 times of the quartile spacing (=P75-P25), which will be sentenced to outlier data. During the analysis, the sensitivity analysis is used for outlier data, namely analyzing outcomes including or excluding, outliers data. and if the results are not contradictory, the data is retained; if the contradiction, it depends on the specific circumstances.
- Descriptive statistics: The measurement data gives the mean, the standard deviation and the confidence interval, and the minimum value, the maximum value, the P25, the median and the P75 are given when necessary; matched data also gives the mean and standard deviation of the gap-value, and the median and average rank of the non-parametric method. The nominal-scale data gives the frequency distribution and the corresponding percentages. The level data gives the frequency distribution and the corresponding percentages, as well as the median and the average rank. Qualitative data give positive rate, positive number, and denominator numbers. The survival data gives the number of events, the number of deletions, the median survival time, and the survival rate.
- Frequencies of causes of first recurrence and death within 3 years after surgery in RDG and

LDG groups were compared with Pearson χ^2 test, then *P* for chi-square was calculated.

- Subgroup analysis: Sub-group analysis is to find the factors that may affect prognostic according to the specific circumstances of the data. For example, subgroup analyses, using log-rank tests, were conducted for disease-free and overall survival stratified by pathologic T stage (ie, pT1, pT2-4) and the LN status (ie, pN0, pN+).
- Missing values handling: This study does not fill in missing values.
- Effective analysis: Using Log-rank test for single factor analysis of survival time data, using Cox regression model analysis for multi-factor analysis. Quantitative data using t test or t' test (variance is not homogeneous), qualitative data using Pearson χ^2 test, grade data using Wilcoxon rank test.
- Safety analysis: counting adverse responds incidence and incidence of adverse events and make a list to describe the adverse events occurring in the study. describe the results of the laboratory tests before and after the normal/abnormal changes and the relationship between the abnormal changes and drugs in the research, and make a list on the "normal/abnormal" changes occurred in the study. More detailed statistical analysis is shown in the statistical analysis plan.
- Continuous variables are expressed as mean (standard deviation (SD)), and categorical variables are expressed as numbers. The differences between the groups were assessed using the t-test or χ^2 test, as appropriate. All tests were two-sided, with a significance level set at P<0.05.
- The 3-year disease-free survival and overall survival rates were calculated using the Kaplan-Meier method, and the log-rank test was used to determine significance. The hazard ratios (HRs) comparing the RDG and LDG groups were estimated using Cox regression after confirmation of the proportional hazards assumption. Multivariate Cox regression analyses were performed to evaluate the effect of surgery type on survival, after adjustment for clinicopathologic covariates that were significantly associated with the outcome in univariate analyses. All-cause mortality was treated as a competing event for recurrence. The cumulative incidence in the presence of competing risks was calculated, and competing-risk survival regression was used as an alternative to Cox regression.

- Landmark analyses were conducted to evaluate the outcomes at 1 year and subsequent follow-up.

Tables and figures

I. Patient demographics and clinical characteristics

Figure 1. Study Flowchart

Table 1. Baseline and Postoperative Characteristics of the ICG Group and Non-ICG Group

- **Age at baseline**
- **BMI**
- **Size**
- **Sex**: male/female
- **ASA score:** 1/2/3
- **ECOG PS**: 0 / 1
- **CEA:** ＜5ng/ml/≥5ng/ml
- **CA19-9:** ＜37U/ml/≥37U/ml
- **Histology:** Differentiated/Undifferentiated
- **Histology:** Differentiated/Undifferentiated
- **cT stage:** cT1-cT3/cT4
- **cN stage:** cN0/cN+
- **pT stage:** pT1-pT3/pT4
- **pN stage:** pN0/pN1/ pN2/pN3a/pN3b
- **AJCC8th staging: I/II/III**

II. Outcome analysis

Table 2. Univariate and Multivariate Cox Regression Analyses of Risk Factors for Disease-free Survival

- The hazard ratios (HRs) comparing the RDL and LDG groups were estimated using Cox regression after confirmation of the proportional hazards assumption. Multivariate Cox regression analyses were performed to evaluate the effect of surgery type on survival, after adjustment for clinicopathologic covariates that were significantly associated with the outcome in univariate analyses.

Table 3. Frequencies of Causes of First Recurrence and Death Within 3 Years After Surgery in RDG and LDG Groups

- Except for all-cause death, the risk difference was calculated by subtracting the cumulative incidence in the first 3 years of the LDG group from that of the RDG group, in presence of competing events; for

all-cause death, the risk difference was calculated by subtracting the 3-year overall survival rate of the LDG group from that of the RDG group.

- Except for all-cause death, competing-risks survival regression was used to derive the hazard ratio, 95% CI, and P value. For total recurrence, all-cause death was the competing event; for the specific types of recurrence, other types of recurrence and death were the competing events; for gastric cancer cause of death, other causes of death were the competing events, and vice versa. Univariate Cox regression was used for all-cause death.
- *P* value for the hazard ratios.
- *P* value for chi-square test was calculated by Pearson χ^2 test.

Figure 2. Kaplan-Meier Curves Comparing Disease-free Survival Between the RDG Group and LDG Group

Fig. S4. Kaplan-Meier Curves Comparing Overall Survival Between the RDG Group and LDG Group

- The 3-year disease-free survival and overall survival rates were calculated using the Kaplan-Meier method, and the log-rank test was used to determine significance.

Fig. S1. Kaplan-Meier Curves Comparing Disease-free Survival Between the RDG Group and LDG Group by Different Pathologic T stage and N stage. (A) patients with pT1 stage; (B) patients with pT2-4 stage; (C) patients with pN0 stage; (D) patients with pN+ stage.

Fig. S5. Kaplan-Meier Curves Comparing Overall Survival Between the RDG Group and LDG Group by Different Pathologic T stage and N stage. (A) patients with pT1 stage; (B) patients with pT2-4 stage; (C) patients with pN0 stage; (D) patients with pN+ stage.

- The 3-year disease-free survival and overall survival rates were calculated using the Kaplan-Meier method, and the log-rank test was used to determine significance.

Fig. S3. Kaplan-Meier Curves Using for Landmark analysis discriminating between events occurring before and after 1 year of follow-up.

- The disease-free survival rates were calculated using the Kaplan-Meier method, and the log-rank test was used to determine significance.

Plan for missing data

For time-to-event outcomes, subjects who withdraw, die, are lost to follow-up or finish the study will be included as censored subjects. Missing data for demographic and clinical variables are not expected.

Summary of Changes to the Statistical Analysis Plan

1. A version number was added.
2. Statistics software version number was updated.
3. Statistics methods were added.
4. The information of statistical analysis of population division was updated
5. Outcome analysis were updated, including the results of subgroup analysis for patients with different pathologic T stage (ie, pT1, pT2-4) and the LN status (ie, pN0, pN+).
